# Fluorination of amino-quinones enhances anti-*Trypanosoma cruzi* activity

**DOI:** 10.1039/d6md00631k

**Published:** 2026-10-05

**Authors:** Zhenhao Zhong, Renata G. Almeida, Victor F. S. Ramos, Andrew J. Lacey, Rubem F. S. Menna-Barreto, Guilherme A. M. Jardim, Eufrânio N. da Silva Júnior, Alastair J. J. Lennox

**Affiliations:** a School of Chemistry, University of Bristol Cantock's Close Bristol BS8 1TS UK a.lennox@bristol.ac.uk; b Instituto de Ciências Exatas, Departamento de Química, Universidade Federal de Minas Gerais CEP 31270-901 Belo Horizonte MG Brazil eufranio@ufmg.br; c Laboratório de Biologia Celular, IOC, FIOCRUZ Rio de Janeiro RJ 21045-900 Brazil

## Abstract

Quinones are well-established antiparasitic agents. Despite the widespread use of fluorine to modulate biological activity and physicochemical properties in drug design, its systematic incorporation into quinoidal frameworks remains underexplored. Here, we report a mild and operationally simple direct electrophilic fluorination of a wide scope of amino-substituted 1,4-naphthoquinones. The fluorinated quinones were evaluated *in vitro* against *Trypanosoma cruzi* (*T. cruzi*), revealing a consistent and pronounced enhancement in trypanocidal activity upon fluorine incorporation compared with their non-fluorinated aminoquinone precursors. Notably, several fluorinated aminoquinone exhibited higher trypanocidal activity than benznidazole, the drug currently used in the clinical treatment of Chagas disease. This work highlights the potential of fluorine to beneficially influence the properties and biological activity of quinoidal systems.

## Abstract 2

Quinones are well-established antiparasitic agents. Here we report a mild and direct method for the fluorination of amino quinones, which show enhanced *in vitro* activity against *Trypanosoma cruzi*.

## Graphical abstract

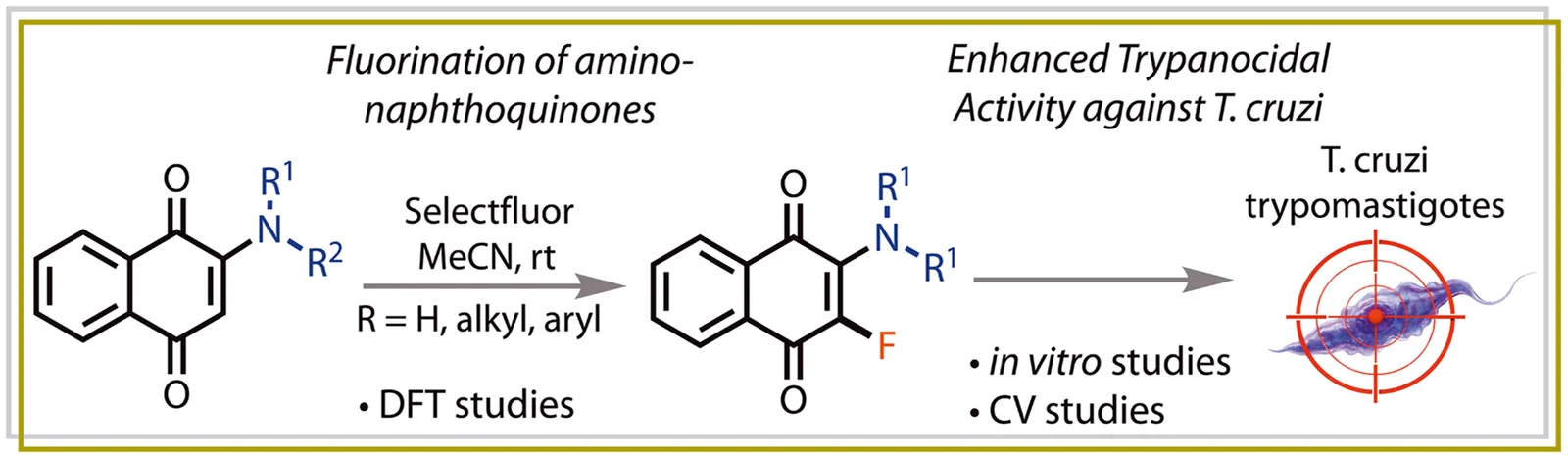

Quinones are a privileged class of redox-active compounds that are important in both biology and technology.^1^ In nature, quinoidal motifs are integral to essential biochemical processes, most notably as electron carriers in cellular respiration and photosynthesis, exemplified by ubiquinone (coenzyme Q) and plastoquinone.^2^ Beyond these physiological roles, naphthoquinones have attracted sustained interest in medicinal chemistry due to their broad spectrum of biological activities, including antitumoral,^3^ antibacterial,^4^ antifungal,^5^ antimalarial^6^ and antiparasitic effects.^7^ Central to this versatility is the ability of the quinone core to undergo reversible one- and two-electron redox processes, enabling the generation of semiquinone radicals and reactive oxygen species (ROS) under biological conditions.^8^ The capability to redox cycle is a key factor that determines biological activity of quinoidal scaffolds.

Among the diseases for which quinones have shown promise, Chagas disease is a compelling and urgent target.^9^ Caused by the protozoan *Trypanosoma cruzi*, Chagas disease remains a major neglected tropical disease affecting millions of people, predominantly in endemic regions of Latin America, despite its increasing global distribution.^10^ Despite its prevalence and severe long-term clinical manifestations, including cardiomyopathy, megacolon and megaoesophagus,^11*a*^ therapeutic options remain limited to benznidazole, Fig. 1A, and nifurtimox, both of which suffer from significant toxicity, limited efficacy and treatment discontinuation.^11^ The pressing need for new drugs with improved efficacy and safety has motivated extensive studies into redox-active compounds capable of disrupting the parasite’s oxidative balance.^12^

**Fig. 1:**
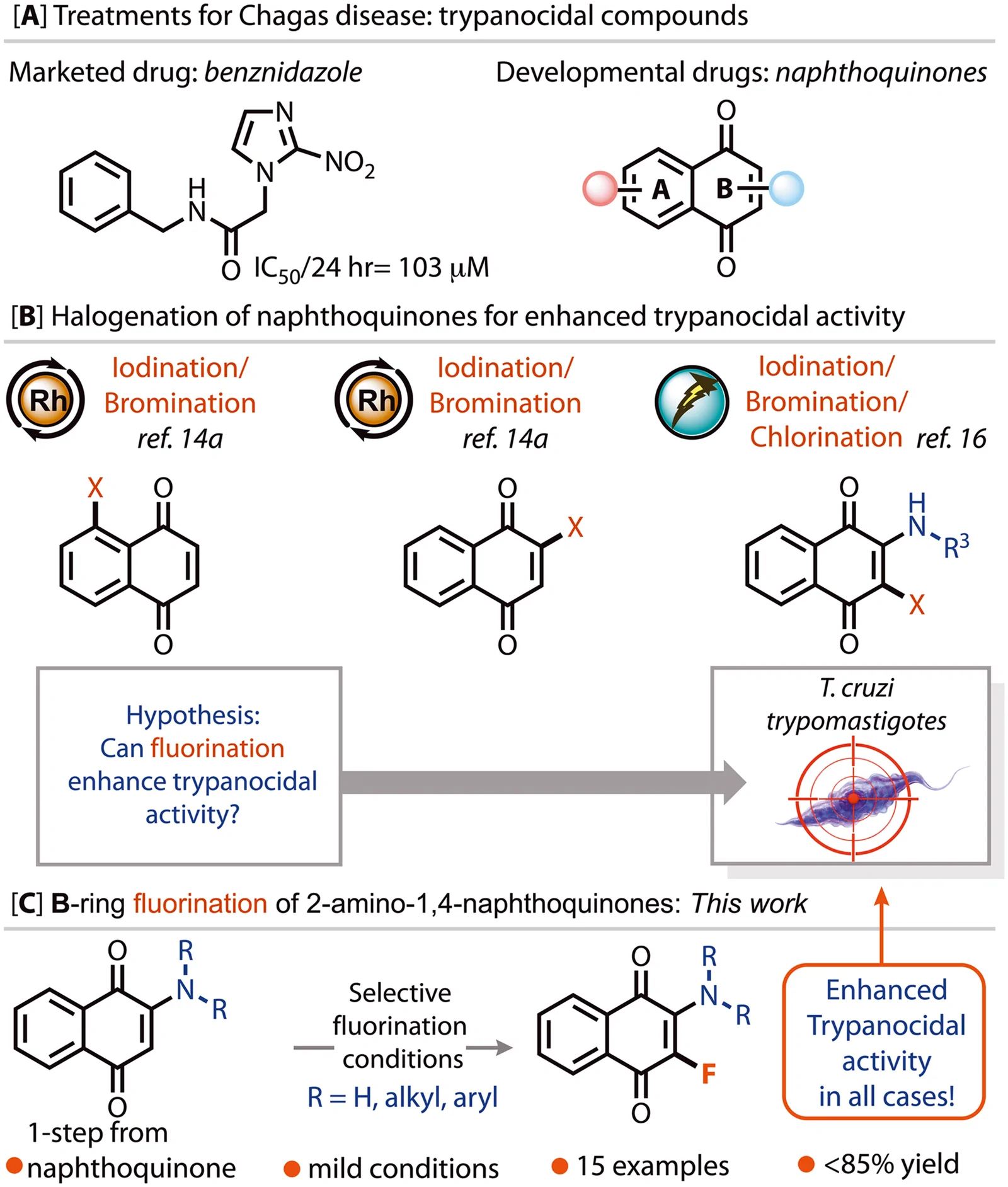
Overview of trypanocidal compounds as (potential) treatments for Chagas disease, including benznidazole, substituted naphthoquinones, and amino-fluorinated naphthoquinones studied in this work.

Naphthoquinones have emerged as particularly attractive candidates against *T. cruzi* with highly tuneable trypanocidal activity, Fig. 1A. This activity is linked to their capacity to perturb the redox homeostasis of the parasite, an organism that exhibits limited antioxidant defences and heightened sensitivity to oxidative stress.^13^ In recent years, our research groups have sought to tune the oxidation potential and stability of naphthoquinones to access structurally diverse libraries with enhanced trypanocidal profiles, Fig. 1B.^14–17^ In particular, amino-halogenation (I/Br/Cl) of the B ring, led to high levels of activity, beyond that of the marketed drug, benznidazole.^16^ Nevertheless, despite these advances and promising bioactivity towards other diseases,^18^ the systematic incorporation of fluorine into quinones remains underexplored, Fig. 1B, particularly when considering the central role of fluorine in contemporary medicinal chemistry.^19–21^

We hypothesised that the strong inductive effect of fluorine would increase the quinoidal oxidation potential and modulate the lifetime of radical intermediates formed during biological redox cycling.^22^ This electronic tuning may directly influence the generation of reactive oxygen species (ROS) within *T. cruzi*, offering a rational strategy to enhance antiparasitic activity while maintaining selectivity.^23^ Fluorination can influence lipophilicity and membrane permeability,^24,25^ which may contribute to the activity against intracellular parasites. Despite these potential advantages, direct C–F bond formation on quinones remains synthetically challenging due to their intrinsic electrophilicity and oxidative sensitivity, and methods for selective fluorination of functionalized quinones are scarce.^26^ Hence, a general fluorination method of amino-functionalised quinones, that preserved structural integrity and functional group tolerance, would be valuable. Here, we report the development of a mild and operationally simple protocol for the direct electrophilic fluorination of amino-substituted 1,4-naphthoquinones. By leveraging controlled C–F bond formation, we access a diverse series of fluorinated quinoidal derivatives and systematically evaluate the impact of fluorination on trypanocidal activity, Fig. 1C. This work provides direct evidence that the incorporation of fluorine into the quinoidal core consistently enhances antiparasitic potency compared with the corresponding nonfluorinated precursors.

We began by developing a practical and selective protocol for the fluorination of amino-substituted 1,4-naphthoquinone (1), which can be readily accessed in a single step from naphthoquinone.^27^ Our first strategy drew upon our experience in hypervalent iodine(iii)-mediated alkene fluorination,^28^ using pyridine·9HF as the nucleophilic fluoride source. This approach successfully gave the desired 2-fluoro-3-amino-1,4-naphtho-quinone 2a in good yield (Fig. 2A). However, we also explored a second, electrophilic strategy that directly applied the use of Selectfluor to the amino-substituted naphthoquinone 1. While the electron-poor naphthoquinone itself disfavours such a process, the presence of the amine group aids fluorination through the nucleophilic enamine functionality. This approach was found to be superior to the nucleophilic approach in terms of practicality, and so the yield and the equivalents of oxidant were further optimised (see SI for full details).

**Fig. 2:**
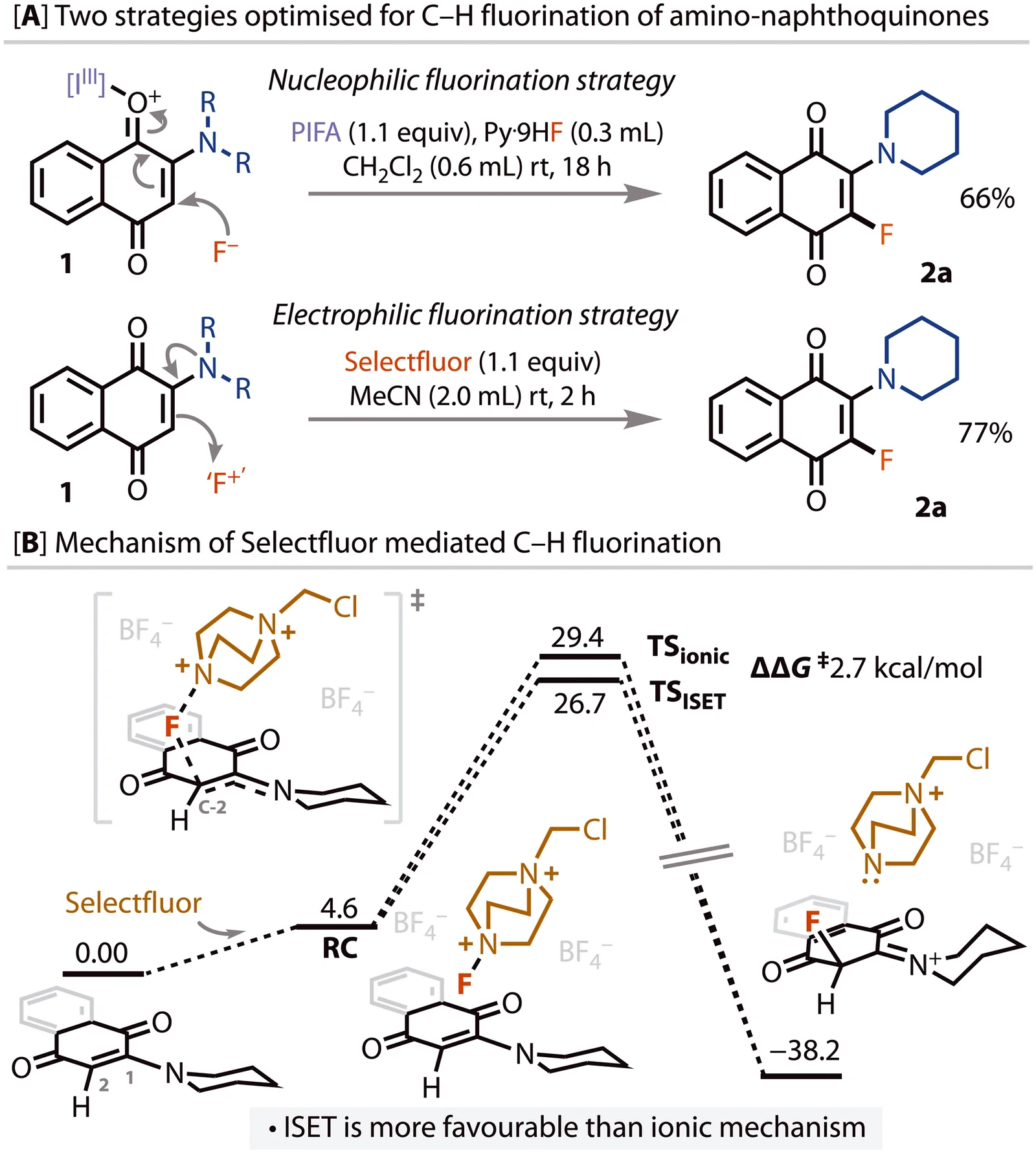
Quinone fluorination with proposed electrophilic mechanism proceeding by a favoured SET mechanism. DFT calculations for the Gibbs free energy reaction profile were carried out at the M06-2X-D3(0)/def2-SVP/SMD(MeCN)//M06-2X-D3(0)/def2-TZVP/SMD(MeCN) level of theory and energies are reported in kcal mol^−1^.

Selectfluor has been shown to react either *via* a polar two-electron S_N_2 ionic process to transfer an F^+^ ion,^29^ or an inner sphere fluorine atom transfer, initiated by a single electron transfer (SET).^30^ This bifurcative mechanistic behaviour depends on the substrate class, transformation and reaction conditions.^31^ To establish which pathway operated for this quinone C–H fluorination reaction, we used density functional theory (DFT) to calculate the difference in energy between each pathway. We found that the transition state for inner-sphere electron transfer (ISET) is energetically stabilised over the closed-shell transition state for S_N_2 (ΔΔ*G*^‡^ 2.7 kcal mol^−1^) relative to the reaction complex (RC). ISET behaviour is indicated by a build-up of spin density at C2, F and both N atoms in TS_ISET_, which was computed under the unrestricted Kohn–Sham formalism to generate a spin-broken wavefunction solution. Interestingly, whilst the N–F interatomic distance is very similar in the two transition state structures, the C2–F distance is much more extended in TS_ISET_ (2.17 Å) compared to TS_ionic_ (1.98 Å).

With the optimised conditions established, the scope of the Selectfluor-mediated fluorination was systematically investigated using a structurally diverse set of amino-substituted 1,4-naphthoquinones, Fig. 3. Cyclic secondary amines were found to be particularly well suited to the transformation. Piperidine-, piperazine- and morpholine-substituted quinones 2a–e were efficiently converted into their corresponding fluorinated derivatives in moderate to good yields. Secondary amine substrate 1h underwent smooth fluorination affording 2h without detectable competitive *N*-fluorination or extensive oxidative degradation. This observation is notable given the intrinsic nucleophilicity and oxidation sensitivity of amines, and highlights the chemoselectivity of the protocol towards C–F bond formation at the quinoidal core. Similarly, both electron-rich and electron-poor aniline-derived substrates 1i–l proved to be competent reaction partners, delivering fluorinated quinones in comparable yields (derivatives 2i–l). The functional group tolerance was demonstrated by the successful fluorination of substrates bearing alkene, alkyne and hydroxyl groups, which each remained intact under the conditions. Interestingly, when the benzylamine (PhNH–) substituted quinone 1o was deliberately subjected to excess Selectfluor, it underwent oxidative debenzylation to give the corresponding primary amine (–NH_2_) 2p.^27*b*^

**Fig. 3:**
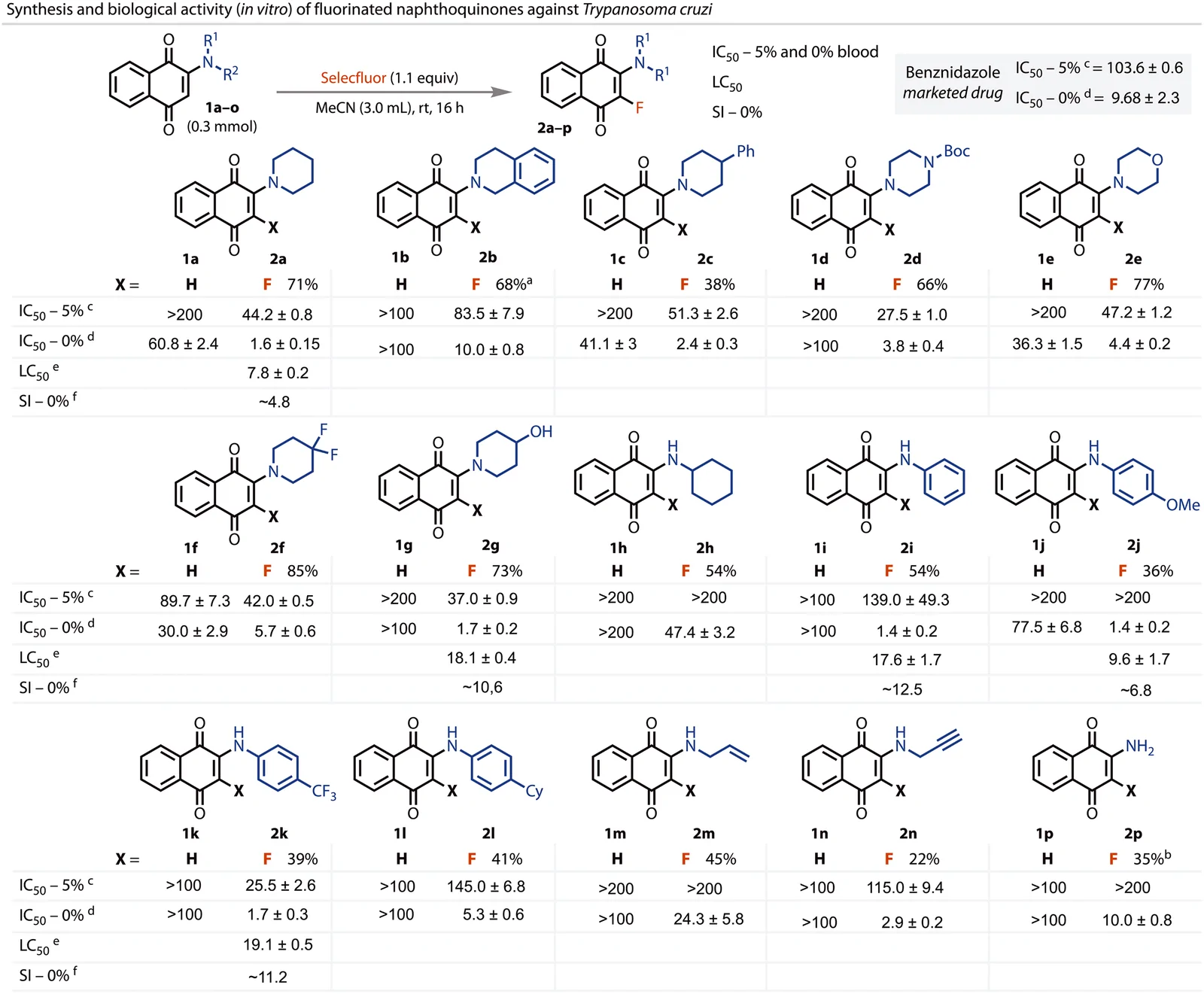
Yields give are isolated yields of fluorination reaction. a) 2 hours reaction time; b) 2.2 equivalents of Selectfluor was used on benzylamine (PhNH–) 1o; c) IC_50_/24 h (μM), 5% of blood, 4 °C; d) IC_50_/24 h (μM), 0% of blood, 37 °C; e) LC_50_/24 h (μM); f) SI 0% of blood 37 °C. IC_50_ values shown are the mean ± SD of at least three independent experiments. The relationship between the trypanocidal activity and the toxicity to the host cells was expressed as the selectivity index (SI) calculated by LC_50_/24 h for uninfected macrophages per IC_50_/24 h for bloodstream trypomastigotes form (0% of blood, 37 °C).

The biological activity (*in vitro*) of the synthesized compounds was evaluated in blood samples containing the *T. cruzi* parasite, Fig. 3, in which the IC_50_, LC_50_ and SI were calculated. The trypanocidal activity was evaluated under two distinct experimental conditions: in the presence of 5% blood at 4 °C and under blood-free conditions at 37 °C. The former provides information on compound activity against bloodstream trypomastigotes under conditions relevant to blood storage, whereas the latter evaluates parasite susceptibility at physiological temperature in the absence of blood-derived components. Differences in potency between these conditions may therefore reflect the combined influence of temperature and the biological matrix on compound availability and activity.

A consistent outcome of this study is that, for all substrate-derivative pairs examined, incorporation of a single fluorine atom into the amino-substituted 1,4-naphthoquinoidal framework led to a clear enhancement of antiparasitic activity when compared with their non-fluorinated counterparts. This uniform trend strongly supports fluorination as a beneficial structural modification for this class of redox-active scaffolds.

Across the series bearing cyclic secondary amines 2a–g, fluorinated derivatives consistently displayed lower IC_50_ values relative to the parent non-fluorinated compounds. Piperidine- and piperazine-substituted fluorinated quinones 2a and 2b showed the greatest increase in potency, indicating a beneficial interaction between the flexible amine group and the modified quinone core. A similar fluorine-dependent improvement was observed for derivative 2h, bearing a secondary amine, and aniline-type substituents 2i–l. In all cases, the fluorinated analogues outperformed their non-fluorinated precursors, although the magnitude of activity enhancement varied depending on the nature of the nitrogen substituent. For aniline-derived compounds, electron-neutral and electron-poor arylamines 2i and 2k afforded fluorinated quinones with superior activity compared to more electron-rich analogues 2j and 2l. This trend suggests that electronic communication between the aniline and the quinone core influences biological performance, potentially by fine-tuning redox behaviour and the stability of radical intermediates formed during biological redox cycling. Notably, the beneficial effect of fluorination was observed irrespective of the overall substitution pattern, indicating that the presence of fluorine exerts a dominant influence on biological activity that supersedes more subtle substituent effects. While more in-depth studies are required to elucidate the role of fluorine, our observations align with the established role of fluorine to modulate electronic properties, metabolic stability and membrane permeability.^24^

We confirmed that fluorine consistently increased the oxidation potential compared to the non-fluorinated quinones by running a series of cyclic voltammetry experiments in DMSO, Fig. 4 and SI. The more oxidising (less negative *E*_1/2_) the quinone, the more active it is. Modulation of the quinoidal redox potential in this way may contribute to the ability of the quinones to participate in intracellular redox cycling and reactive oxygen species (ROS) generation,^32^ which are key contributors to trypanocidal activity. In addition, we also found that fluorine increases the lipophilicity of these quinones as the cLog *P* increased by 0.17 (see SI for details), which may help to facilitate cellular uptake, further contributing to the observed activity enhancement.^33^ It should be noted that the relationship between redox potential, fluorination, and trypanocidal activity is correlative at this stage, as the direct assessment of ROS production and oxidative stress will be required to confirm the proposed mechanism of action.

**Fig. 4:**
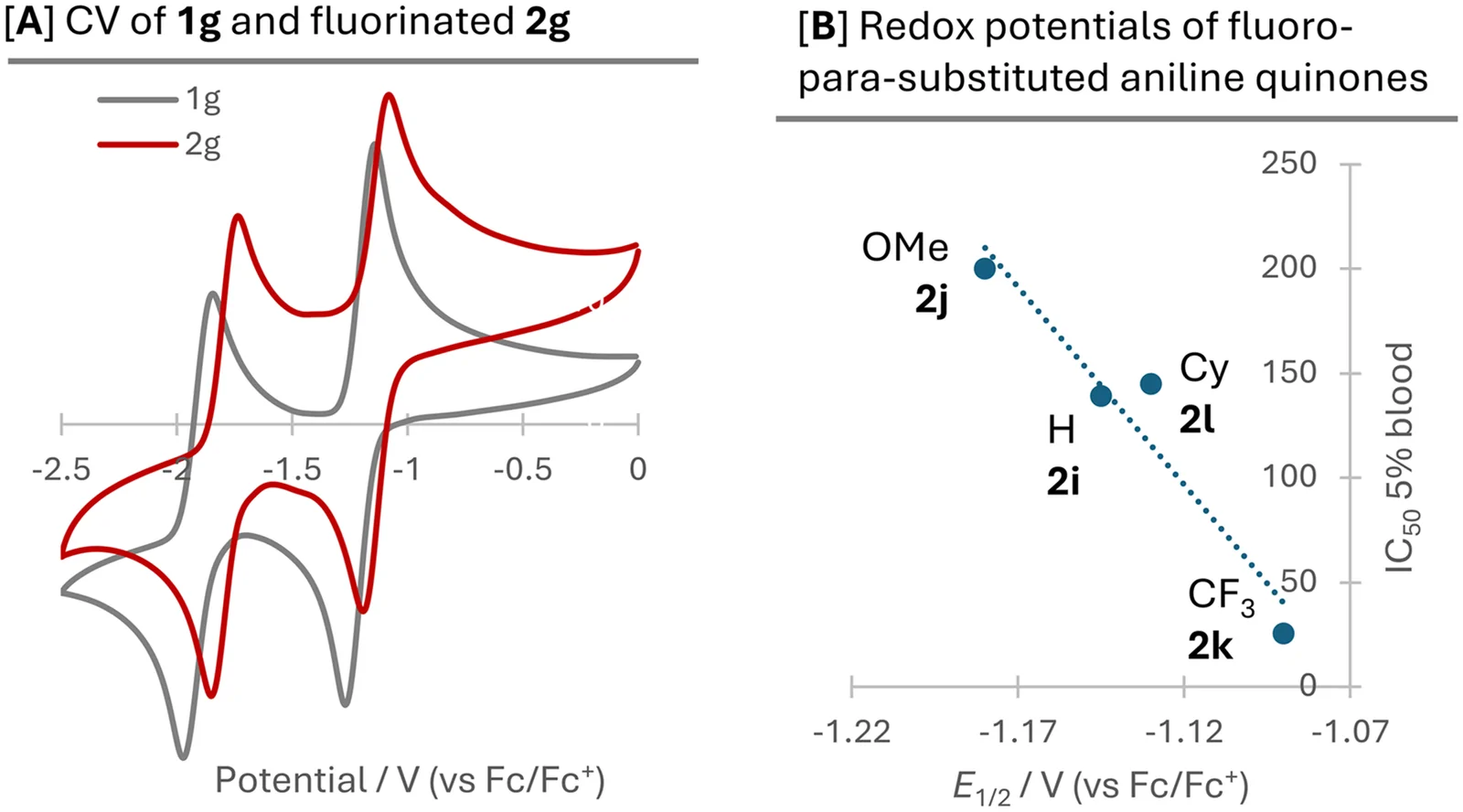
Cyclic voltammetry of amino-quinones and their fluorinated derivatives run at 100 mV s^−1^ at a glassy carbon working electrode in DMSO.

In summary, we have developed a practical, chemoselective and broadly applicable strategy for the direct electrophilic fluorination of amino-substituted 1,4-naphthoquinones. Our protocol exhibits wide functional group tolerance and accommodates a diverse range of amines. Biological evaluation against *Trypanosoma cruzi* revealed that fluorination consistently enhances antiparasitic activity across all substrate classes. This uniform improvement strongly supports C–F bond incorporation as a general and effective lead-optimisation strategy to improve the potency of quinoidal trypanocidal agents, which may benefit medicinal chemistry programs targeting Chagas disease, as well as other quinone-based therapeutic areas.

## Conflicts of interest

There are no conflicts to declare.

## Supplementary Material

MD-OLF-D6MD00631K-s001

## Data Availability

The data supporting this article are included in the supplementary information (SI). Supplementary information is available. See DOI: https://doi.org/10.1039/d6md00631k.
