# Supplementary material for: Fluorination of amino-quinones enhances anti-Trypanosoma cruzi activity
Source: RSC Med Chem. 2026 Oct 5. Online ahead of print. doi: 10.1039/d6md00631k (PMC13636235; doi:10.1039/d6md00631k)
Supplement: Supplementary file 1 [file MD-OLF-D6MD00631K-s001.pdf]

Supplementary Information (SI) for RSC Medicinal Chemistry.
This journal is © The Royal Society of Chemistry 2026

Supporting Information

Fluorination of Amino-Quinones Enhances anti-*Trypanosoma cruzi* Activity

Zhenhao Zhong,[a] Renata G. Almeida,[b] Victor F. S. Ramos,[c] Andrew J. Lacey,[a] Rubem F. S. Menna-Barreto,[c] Guilherme A. M. Jardim,[b] Eufrânio N. da Silva Júnior*[b] and Alastair J. J. Lennox*[a]

[a] *School of Chemistry, University of Bristol, Cantock's Close, Bristol, BS8 1TS, United Kingdom. E-mail: a.lennox@bristol.ac.uk.*

[b] *Instituto de Ciências Exatas, Departamento de Química, Universidade Federal de Minas Gerais, CEP 31270-901, Belo Horizonte, MG, Brazil. E-mail: eufranio@ufmg.br.*

[c] *Laboratory of Cellular Biology, IOC, FIOCRUZ, Rio de Janeiro, RJ, 21045-900, Brazil.*

Table of Content

General Experimental Considerations..........3

Experimental and Characterisation Data..........4

Computational Methods..........23

Cartesian Cooridinates..........24

Cytotoxicity and anti-*Trypanosoma cruzi* activity assays..........34

Animals..........34

Cytotoxicity to mammalian cells..........34

Trypanocidal assays..........34

Cyclic Voltammetry..........36

Cyclic voltammetry plots of measured compounds..........36

cLogP values..........40

References..........44

NMR spectra of novel compounds..........45

General Experimental Considerations

All reactions were run under an inert atmosphere (argon) with flame-dried glassware using standard techniques unless otherwise stated. All commercial reagents were used as supplied or purified by standard techniques where necessary. Flash column chromatography was performed using 230-400 mesh silica with the indicated solvent system according to standard techniques. Analytical thin-layer chromatography (TLC) was performed on precoated, aluminum-backed silica gel plates. Visualisation of the developed chromatogram was performed by UV absorbance (254 nm) or aqueous potassium permanganate stain. NMR spectra were recorded on Bruker Nano 400 or Bruker Advance III HD 500 cryo spectrometers. Chemical shifts for ^{1}H NMR spectra are recorded in parts per million from tetramethylsilane with the solvent resonance as the internal standard (chloroform: δ = 7.26 ppm, methanol: δ = 3.31 ppm). Data is reported as follows: chemical shift [multiplicity (s = singlet, d = doublet, t = triplet, q = quartet, pent = pentet, m = multiplet), coupling constant in Hz, integration]. ^{13}C NMR spectra were recorded with complete proton decoupling. Chemical shifts are reported in parts per million from tetramethylsilane with the solvent resonance as the internal standard (chloroform: δ = 77.0 ppm, methanol: δ = 44.0 ppm). ^{19}F NMR spectra were recorded with complete proton decoupling unless otherwise stated. ^{19}F NMR spectra are indirectly referenced to $CFCl_3$ automatically via direct measurement of the absolute frequency of the deuterium lock signal by the spectrometer hardware. *J* values are reported in Hz. High resolution mass spectra (HRMS) were recorded on a VG Analytical Autospec spectrometer by Electron Ionisation (EI) or Chemical Ionisation (CI) or on a Bruker micrOTOF instrument by Electrospray Ionisation (ESI). Infrared spectra were recorded using a Perkin Elmer Spectrum Two FT-IR spectrometer.

Experimental and Characterisation Data

General procedure A for the synthesis of 2-amino-1,4-naphthoquinone (1a–i, 1m–n)

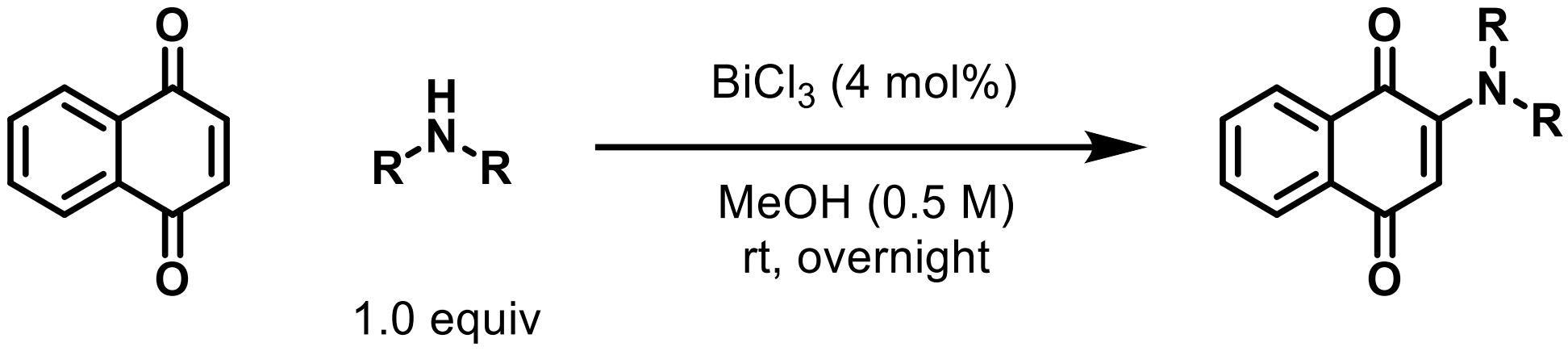

The substrates were prepared according to a literature procedure.[1] Amine (1.0 equiv) and $BiCl_3$ (4 mol%) was added subsequently to a stirred solution of 1,4-naphthoquinone (1.0 equiv) in MeOH (0.5 M). The resulting mixture was stirred at rt overnight. The solvent was concentrated under reduced pressure and purified by flash column chromatography to afford the corresponding 2-amino-1,4-naphthoquinone (**1a**–**i**, **1m**–**n**).

General procedure B for the synthesis of 2-amino-1,4-naphthoquinone (1j–l)

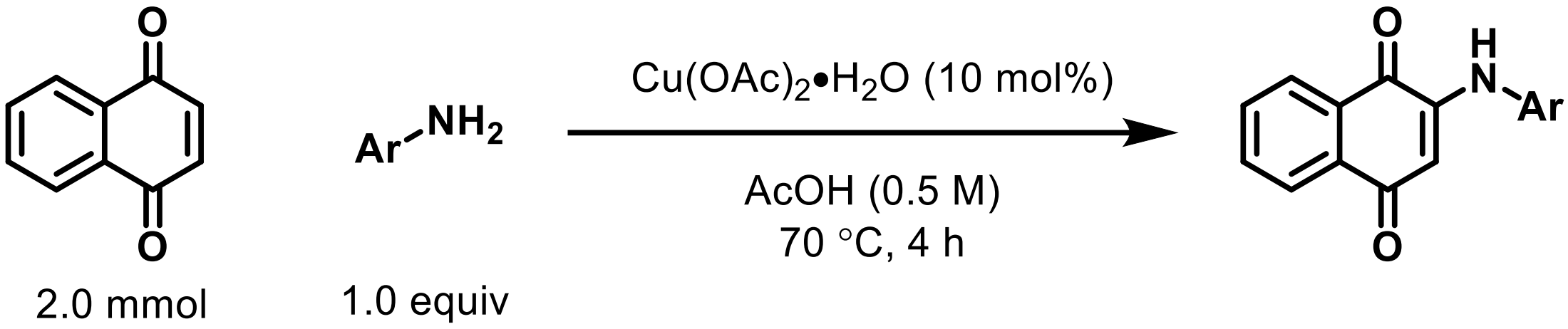

The substrates were prepared according to a literature procedure.[2] $Cu(OAc)_2{\cdot}H_2O$ (40 mg, 0.2 mmol, 10 mol%) was added to a stirred solution of 1,4-naphthoquinone (316 mg, 2.0 mmol, 1.0 equiv) and aniline (2.0 mmol, 1.0 equiv) in acetic acid (4 mL, 0.5 M) at rt. The resulting mixture was heated to 70 °C and stirred for 4 h. The solvent was concentrated under reduced pressure and purified by flash column chromatography to afford the 2-amino-1,4-naphthoquinone (**1j**–**l**).

General procedure C for the fluorination of 2-amino-1,4-naphthoquinone (2a-p)

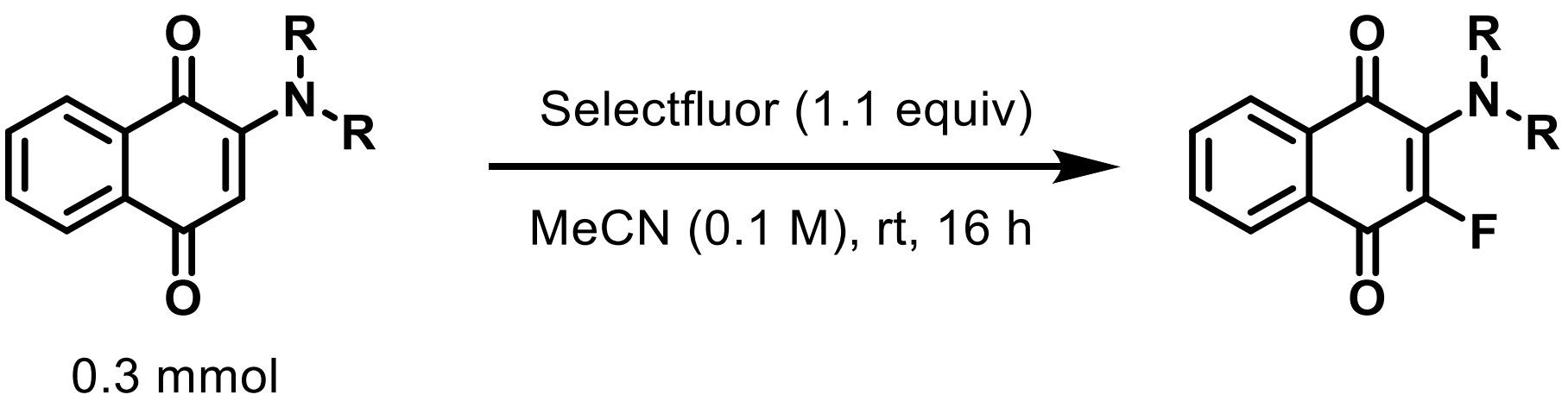

Selectfluor (78 mg, 0.22 mmol, 1.1 equiv) was added to a stirred solution of 2-amino-1,4-naphthoquinone (0.2 mmol, 1.0 equiv) in MeCN (0.1 M) at rt. The resulting mixture was stirred at rt for 16 h. The solvent was concentrated under reduced pressure, diluted with H_2O (5 mL) and extracted with CH_2Cl_2 (3 × 5 mL). The combined organic layer was dried

with $MgSO_4$, filtered and concentrated under reduced pressure. Purification by flash column chromatography (SiO_2) afforded fluorinated amino-quinones (**2a**–**p**).

2-(Piperidin-1-yl)naphthalene-1,4-dione (**1a**)[1]

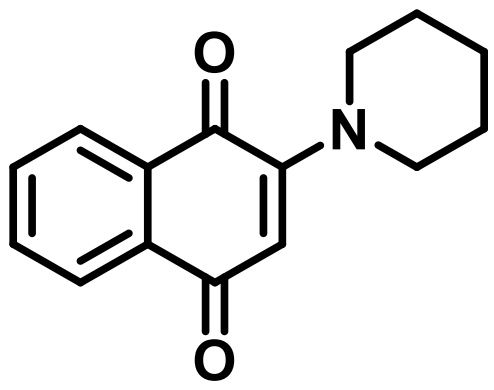

Prepared using General Procedure A on a 10 mmol scale. Purification by flash column chromatography (SiO_2, 20% EtOAc in pentane) afforded **1a** as a red solid (1.05 g, 44%).

R_f 0.3 (20% EtOAc in pentane).

^{1}H NMR (400 MHz, $CDCl_3$) δ 8.04–8.02 (m, 1H), 7.99–7.96 (m, 1H), 7.67 (td, *J* = 7.5, 1.4 Hz, 1H), 7.61 (td, *J* = 7.5, 1.4 Hz, 1H), 6.02 (s, 1H), 3.50–3.47 (m, 4H), 1.79–1.68 (m, 6H)

^{13}C NMR (101 MHz, $CDCl_3$) δ 183.5, 183.4, 154.1, 133.7, 132.9, 132.6, 132.2, 126.6, 125.4, 110.4, 50.4, 25.7, 24.3.

Analytical data (NMR) in correspond with those reported in literature.[3]

2-(3,4-Dihydroisoquinolin-2(1*H*)-yl)naphthalene-1,4-dione (**1b**)[1]

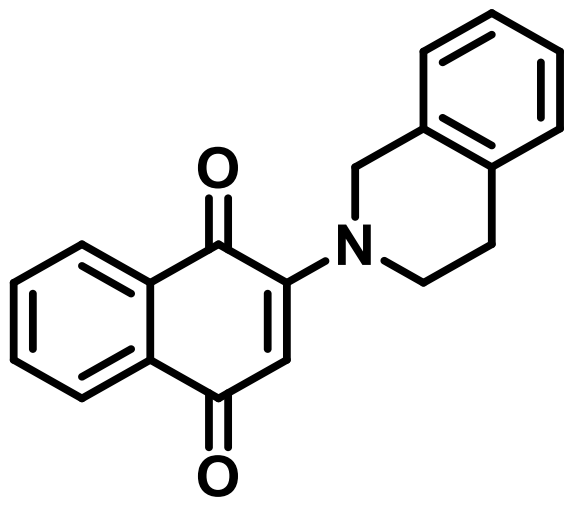

Prepared using General Procedure A on a 5 mmol scale. Purification by flash column chromatography (SiO_2, 20% EtOAc in pentane) afforded **1b** as a brown solid (0.93 g, 65%).

R_f 0.2 (20% EtOAc in pentane)

M.p. 117–119 °C.

IR (film)/cm^{-1} 2981, 1674, 1627, 1566, 1592, 1341, 1293, 1262, 1017, 975, 830, 777, 748, 725, 670.

^{1}H NMR (400 MHz, $CDCl_3$) δ 8.04 (dd, *J* = 7.6, 1.4 Hz, 1H), 8.01 (dd, *J* = 7.6, 1.4 Hz, 1H), 7.69 (td, *J* = 7.4, 1.4 Hz, 1H), 7.63 (td, *J* = 7.5, 1.5 Hz, 1H), 7.24–7.18 (m, 3H), 7.16–7.13 (m, 1H), 6.04 (s, 1H), 4.66 (s, 2H), 3.83 (t, *J* = 5.8 Hz, 2H), 3.05 (t, *J* = 5.8 Hz, 2H).

^{13}C NMR (101 MHz, CDCl$_3$) δ 183.4, 183.2, 152.6, 134.8, 133.9, 132.8, 132.7, 132.5, 132.2, 128.4, 127.1, 126.6, 126.5, 126.4, 125.5, 108.8, 50.8, 47.3, 28.8.

HRMS (ESI) m/z Calcd. for $C_{19}H_{16}NO_2$ [M+H]$^+$: 290.1176, found: 290.1164.

2-(4-Phenylpiperidin-1-yl)naphthalene-1,4-dione (**1c**)[1]

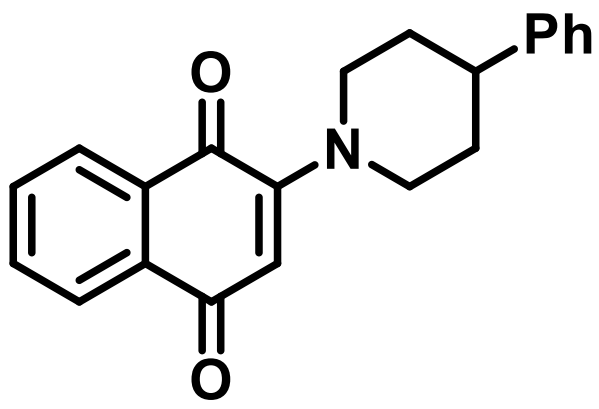

Prepared using General Procedure A on a 5 mmol scale. Purification by flash column chromatography (SiO_2, 20% EtOAc in pentane) afforded **1c** as a black solid (0.94 g, 59%).

R*f* 0.25 (20% EtOAc in pentane).

M.p. 135–138 °C.

IR (film)/cm^{-1} 1674, 1634, 1593, 1568, 1444, 1392, 1334, 1272, 1241, 1205, 974, 849, 781, 759, 702, 537.

^{1}H NMR (400 MHz, CDCl$_3$) δ 8.06–8.04 (m, 1H), 8.02–8.00 (m, 1H), 7.69 (td, *J* = 7.5, 1.5 Hz, 1H), 7.64 (td, *J* = 7.5, 1.5 Hz, 1H), 7.35–7.31 (m, 2H), 7.26–7.22 (m, 3H), 6.09 (s, 1H), 4.22–4.17 (m, 2H), 3.07 (ddd, *J* = 12.9, 11.4, 3.5 Hz, 2H), 2.82 (tt, *J* = 11.3, 4.6 Hz, 1H), 1.98–1.94 (m, 4H).

^{13}C NMR (101 MHz, CDCl$_3$) δ 183.6, 183.3, 154.0, 145.0, 133.8, 132.9, 132.5, 132.3, 128.6, 126.7, 126.6, 126.5, 125.5, 111.2, 50.0, 42.5, 33.0.

HRMS (ESI) m/z Calcd. for $C_{21}H_{19}NO_2$ [M]$^+$: 317.1410, found: 317.1403.

tert-Butyl 4-(1,4-dioxo-1,4-dihydronaphthalen-2-yl)piperazine-1-carboxylate (**1d**)[1]

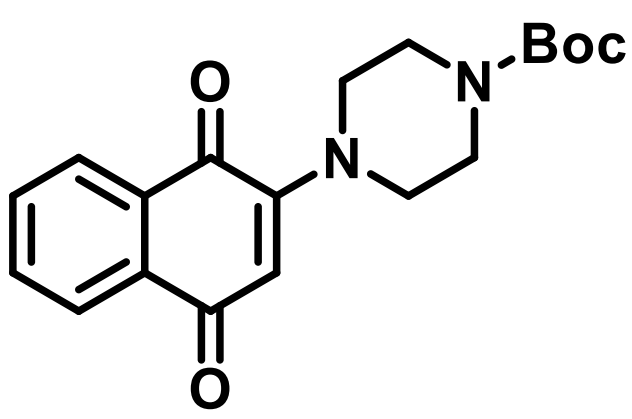

Prepared using General Procedure A on a 5 mmol scale. Purification by flash column chromatography (SiO_2, 30% EtOAc in pentane) afforded **1d** as a brown solid (1.31 g, 77%).

R*f* 0.1 (20% EtOAc in pentane)

M.p. 153–155 °C.

IR (film)/cm^{-1} 2980, 1693, 1673, 1628, 1567, 1411, 1241, 1178, 1121, 980, 727.

^{1}H NMR (400 MHz, $CDCl_3$) δ 8.03 (ddd, *J* = 7.6, 1.5, 0.5 Hz, 1H), 8.00 (ddd, *J* = 7.4, 1.6, 0.5 Hz, 1H), 7.70 (td, *J* = 7.5, 1.5 Hz, 1H), 7.64 (td, *J* = 7.5, 1.5 Hz, 1H), 6.00 (s, 1H), 3.62–3.60 (m, 4H), 3.49 (dd, *J* = 6.7, 3.8 Hz, 4H), 1.48 (s, 9H).

^{13}C NMR (101 MHz, $CDCl_3$) δ 183.6, 182.9, 154.5, 153.4, 134.0, 132.6, 132.6, 132.2, 126.7, 125.6, 111.7, 80.3, 48.6, 28.4.

HRMS (ESI) m/z Calcd. for $C_{19}H_{23}N_2O_4$ $[M+H]^+$: 343.1652, found: 343.1646.

2-Morpholinonaphthalene-1,4-dione (**1e**)[1]

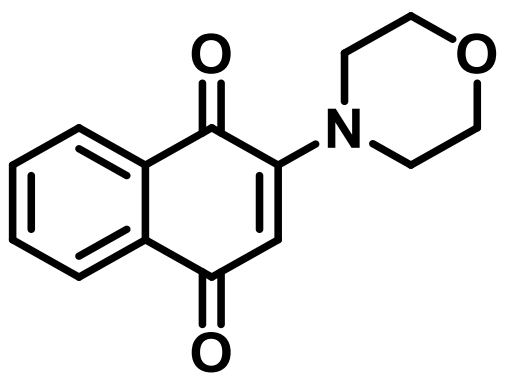

Prepared using General Procedure A on a 5 mmol scale. Purification by flash column chromatography (SiO_2, 20% EtOAc in pentane) afforded **1e** as a brown solid (0.83 g, 69%).

R*f* 0.2 (30% EtOAc in pentane)

^{1}H NMR (400 MHz, $CDCl_3$) δ 8.05–8.03 (m, 1H), 8.00 (ddd, *J* = 7.4, 1.5, 0.5 Hz, 1H), 7.70 (td, *J* = 7.5, 1.5 Hz, 1H), 7.65 (td, *J* = 7.5, 1.5 Hz, 1H), 6.02 (s, 1H), 3.88–3.85 (m, 4H), 3.51–3.49 (m, 4H).

^{13}C NMR (101 MHz, $CDCl_3$) δ 183.8, 182.9, 153.6, 134.0, 132.7, 132.6, 132.2, 126.7, 125.6, 112.0, 66.4, 49.1.

Analytical data (NMR) in correspond with those reported in literature.[3]

2-(4,4-Difluoropiperidin-1-yl)naphthalene-1,4-dione (**1f**)[1]

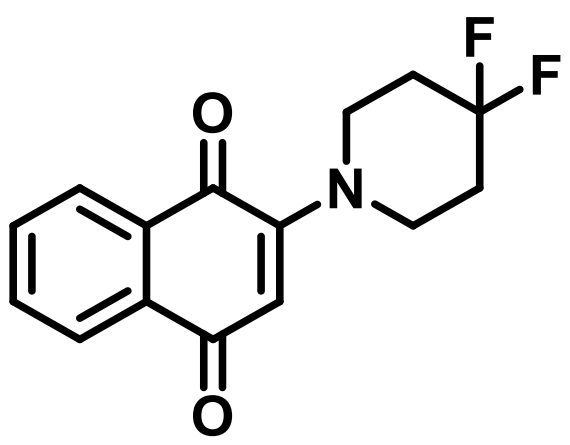

Prepared using General Procedure A on a 5 mmol scale. Purification by flash column chromatography (SiO_2, 10% EtOAc in pentane) afforded **1f** as a red solid (0.84 g, 61%).

R_f 0.45 (20% EtOAc in pentane).

M.p. 139–142 °C.

IR (film)/cm^{-1} 2981, 1630, 1561, 1235, 1108, 933, 779, 725, 657, 616.

^{1}H NMR (400 MHz, CDCl$_3$) δ 8.05–8.02 (m, 1H), 8.01–7.99 (m, 1H), 7.70 (td, *J* = 7.5, 1.5 Hz, 1H), 7.65 (td, *J* = 7.5, 1.5 Hz, 1H), 6.07 (s, 1H), 3.61–3.58 (m, 4H), 2.21–2.11 (m, 4H).

^{13}C NMR (101 MHz, CDCl$_3$) δ 183.7, 182.8, 153.1, 134.0, 132.7, 132.6, 132.2, 126.7, 125.6, 121.1 (t, *J* = 242.1 Hz), 112.7, 46.0 (t, *J* = 5.4 Hz), 33.9 (t, *J* = 23.5 Hz).

^{19}F NMR (377 MHz, CDCl$_3$) δ –97.8.

HRMS (ESI) m/z Calcd. for $C_{15}H_{14}F_2NO_2$ [M+H]$^+$: 278.0987, found: 278.0984.

2-(4-Hydroxypiperidin-1-yl)naphthalene-1,4-dione (**1g**)[1]

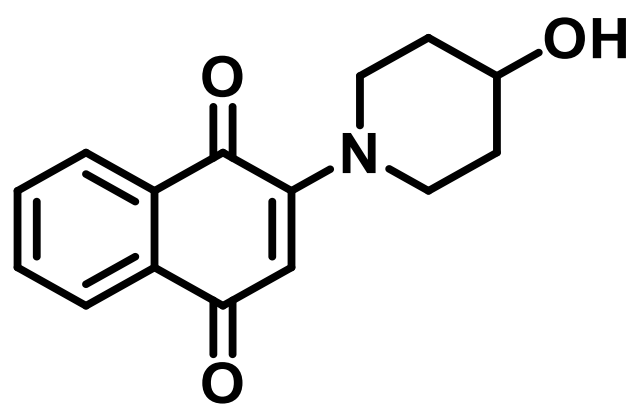

Prepared using General Procedure A on a 5 mmol scale. Purification by flash column chromatography (SiO_2, 60% EtOAc in pentane) afforded **1g** as a red solid (0.68 g, 53%).

R_f 0.1 (60% EtOAc in pentane)

M.p. 120–124 °C.

IR (film)/cm^{-1} 2981, 1672, 1642, 1570, 1270, 1077, 1044, 969, 777, 723.

^{1}H NMR (400 MHz, CDCl$_3$) δ 8.02 (dd, *J* = 7.6, 1.4 Hz, 1H), 7.98 (dd, *J* = 7.5, 1.5 Hz, 1H), 7.68 (td, *J* = 7.5, 1.4 Hz, 1H), 7.62 (td, *J* = 7.5, 1.5 Hz, 1H), 6.04 (s, 1H), 4.01 (tt, *J* = 7.9, 3.9 Hz, 1H), 3.83 (ddd, *J* = 13.1, 6.8, 3.8 Hz, 2H), 3.29 (ddd, *J* = 12.8, 8.8, 3.4 Hz, 2H), 2.07–2.00 (m, 2H), 1.87 (s, 1H), 1.73 (dtd, *J* = 16.9, 8.3, 3.7 Hz, 2H).

^{13}C NMR (101 MHz, CDCl$_3$) δ 183.6, 183.2, 153.8, 133.8, 132.8, 132.4, 132.4, 126.6, 125.5, 111.0, 66.6, 46.4, 33.9.

HRMS (ESI) m/z Calcd. for $C_{15}H_{16}NO_3$ [M+H]$^+$: 258.1125, found: 258.1124.

2-(Cyclohexylamino)naphthalene-1,4-dione (**1h**)[1]

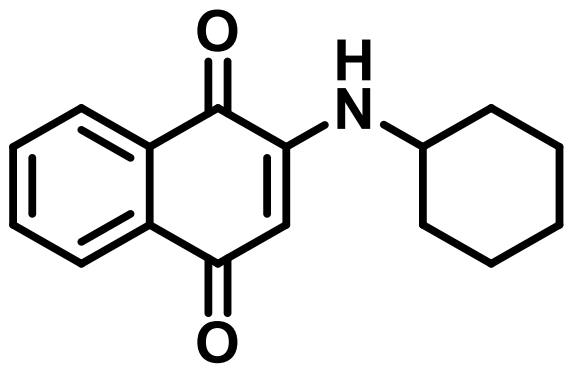

Prepared using General Procedure A on a 5 mmol scale. Purification by flash column chromatography (SiO_2, 20% EtOAc in pentane) afforded **1h** as a red solid (0.56 g, 44%).

R*f* 0.5 (20% EtOAc in pentane)

^{1}H NMR (400 MHz, $CDCl_3$) δ 8.09 (ddd, *J* = 7.7, 1.4, 0.5 Hz, 1H), 8.03 (ddd, *J* = 7.6, 1.4, 0.5 Hz, 1H), 7.71 (td, *J* = 7.6, 1.4 Hz, 1H), 7.59 (td, *J* = 7.5, 1.3 Hz, 1H), 5.84 (d, *J* = 7.9 Hz, 1H), 5.75 (d, *J* = 0.7 Hz, 1H), 3.29 (tdd, *J* = 12.7, 7.8, 3.8 Hz, 1H), 2.03 (ddd, *J* = 8.5, 5.4, 2.6 Hz, 2H), 1.79 (dt, *J* = 12.8, 3.9 Hz, 2H), 1.69–1.64 (m, 1H), 1.46–1.18 (m, 5H).

^{13}C NMR (101 MHz, $CDCl_3$) δ 182.9, 182.1, 146.7, 134.7, 133.7, 131.8, 130.5, 126.2, 126.1, 100.7, 51.1, 31.9, 25.4, 24.5.

Analytical data (NMR) in correspond with those reported in literature.[4]

2-(Phenylamino)naphthalene-1,4-dione (**1i**)[1]

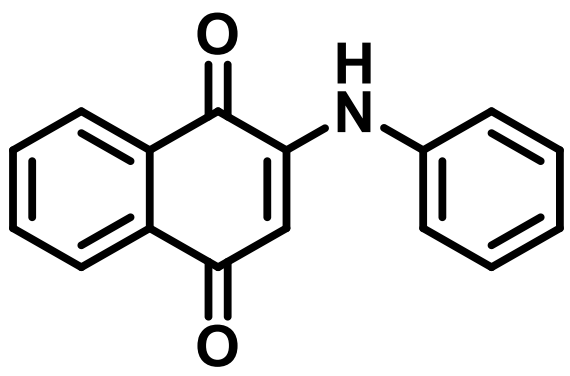

Prepared using General Procedure A on a 5 mmol scale. Purification by flash column chromatography (SiO_2, 20% EtOAc in pentane) afforded naphthoquinone **1i** as a red solid (0.63 g, 51%).

R*f* 0.5 (20% EtOAc in pentane)

^{1}H NMR (400 MHz, $CDCl_3$) δ 8.13–8.08 (m, 2H), 7.76 (td, *J* = 7.6, 1.3 Hz, 1H), 7.67 (td, *J* = 7.5, 1.3 Hz, 1H), 7.57 (s, 1H), 7.45–7.40 (m, 2H), 7.29–7.26 (m, 2H), 7.24–7.19 (m, 1H), 6.42 (s, 1H).

^{13}C NMR (101 MHz, $CDCl_3$) δ 183.9, 182.1, 144.7, 137.4, 134.9, 133.2, 132.3, 130.4, 129.7, 126.5, 126.1, 125.6, 122.6, 103.4.

Analytical data (NMR) in correspond with those reported in literature.[3]

2-((4-Methoxyphenyl)amino)naphthalene-1,4-dione (**1j**)[2]

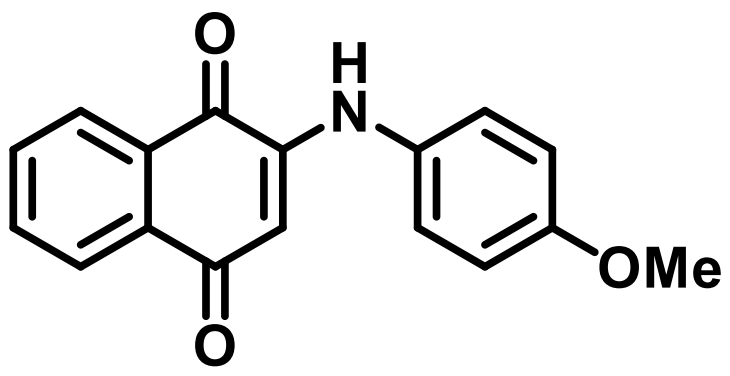

Prepared using General Procedure B on a 2 mmol scale. Purification by flash column chromatography (SiO_2, CH_2Cl_2) afforded **1j** as a black solid (0.31 g, 55%).

R*f* 0.3 (20% EtOAc in pentane)

^{1}H NMR (400 MHz, $CDCl_3$) δ 8.10 (ddd, *J* = 7.2, 5.4, 1.3 Hz, 2H), 7.75 (td, *J* = 7.6, 1.3 Hz, 1H), 7.65 (td, *J* = 7.6, 1.3 Hz, 1H), 7.43 (s, 1H), 7.21–7.18 (m, 2H), 6.96–6.93 (m, 2H), 6.22 (s, 1H), 3.83 (s, 3H).

^{13}C NMR (101 MHz, $CDCl_3$) δ 183.7, 182.2, 157.7, 145.7, 134.9, 133.4, 132.2, 130.4, 130.0, 126.4, 126.1, 124.8, 114.9, 102.5, 55.5.

Analytical data (NMR) in correspond with those reported in literature.[2]

2-((4-(Trifluoromethyl)phenyl)amino)naphthalene-1,4-dione (**1k**)[2]

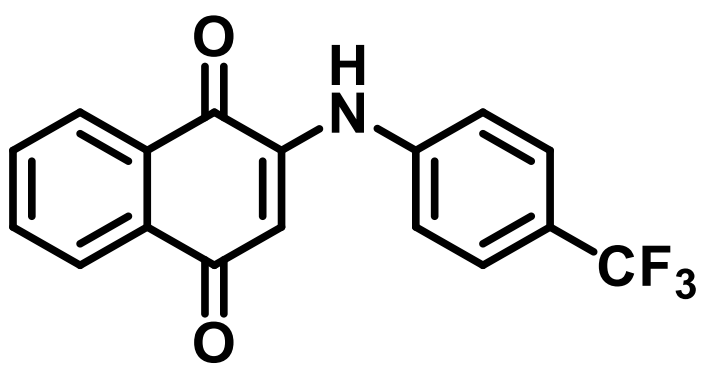

Prepared using General Procedure B on a 2 mmol scale. Purification by flash column chromatography (SiO_2, CH_2Cl_2) afforded **1k** as a red solid (0.27 g, 42%).

R*f* 0.5 (20% EtOAc in pentane)

^{1}H NMR (400 MHz, $CDCl_3$) δ 8.16–8.12 (m, 2H), 7.79 (td, *J* = 7.5, 1.3 Hz, 1H), 7.72–7.67 (m, 4H), 7.39 (d, *J* = 8.4 Hz, 2H), 6.53 (s, 1H).

^{13}C NMR (101 MHz, $CDCl_3$) δ 184.0, 181.7, 143.7, 140.9, 135.1, 130.2, 127.1 (q, *J* = 271.3 Hz), 127.0 (q, *J* = 3.8 Hz), 126.8, 126.7, 126.3, 121.6, 104.9.

^{19}F NMR (377 MHz, $CDCl_3$) δ –62.3.

Analytical data (NMR) in correspond with those reported in literature.[2]

2-((4-Cyclohexylphenyl)amino)naphthalene-1,4-dione (**1l**)[2]

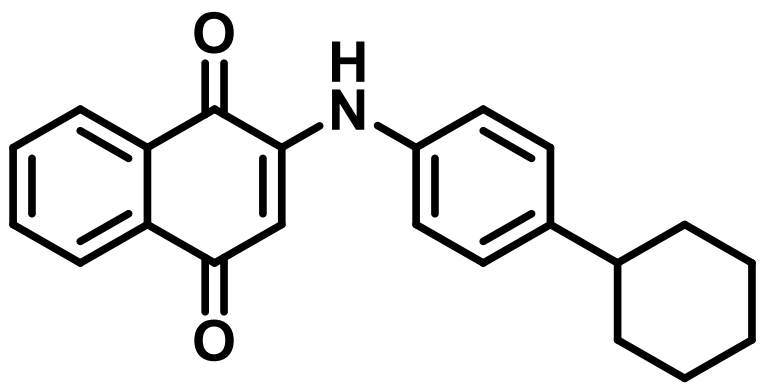

Prepared using General Procedure B on a 2 mmol scale. Purification by flash column chromatography (SiO_2, CH_2Cl_2) afforded **1l** as a red solid (0.37 g, 56%).

R*f* 0.6 (20% EtOAc in pentane)

^{1}H NMR (400 MHz, $CDCl_3$) δ 8.13–8.10 (m, 2H), 7.76 (td, *J* = 7.5, 1.4 Hz, 1H), 7.66 (td, *J* = 7.5, 1.3 Hz, 1H), 7.51 (s, 1H), 7.26–7.18 (m, 4H), 6.38 (s, 1H), 2.54–2.49 (m, 1H), 1.89–1.84 (m, 4H), 1.76 (ddt, *J* = 13.7, 4.5, 2.1 Hz, 1H), 1.41 (td, *J* = 9.2, 2.7 Hz, 4H), 1.31–1.24 (m, 1H).

^{13}C NMR (101 MHz, $CDCl_3$) δ 183.9, 182.2, 145.9, 145.0, 135.0, 134.9, 133.3, 132.2, 130.4, 128.0, 126.5, 126.1, 122.8, 103.1, 44.1, 34.4, 26.8, 26.1.

Analytical data (NMR) in correspond with those reported in literature.[5]

2-(Allylamino)naphthalene-1,4-dione (**1m**)[1]

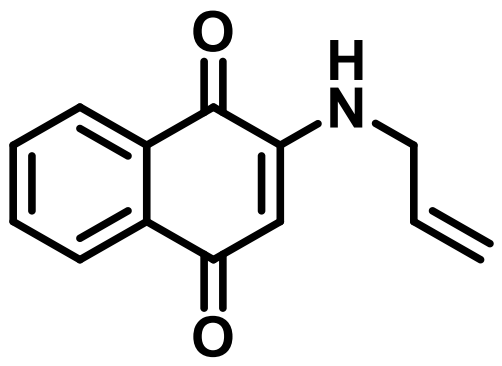

Prepared using General Procedure A on a 20 mmol scale. Purification by flash column chromatography (SiO_2, 20% EtOAc in pentane) afforded **1m** as a red solid (2.47 g, 58%).

R*f* 0.3 (20% EtOAc in pentane)

^{1}H NMR (400 MHz, $CDCl_3$) δ 8.10 (dd, *J* = 7.7, 1.3 Hz, 1H), 8.05 (dd, *J* = 7.7, 1.3 Hz, 1H), 7.73 (td, *J* = 7.5, 1.3 Hz, 1H), 7.62 (td, *J* = 7.5, 1.3 Hz, 1H), 6.01 (s, 1H), 5.88 (ddt, *J* = 17.2, 10.6, 5.5 Hz, 1H), 5.75 (s, 1H), 5.34–5.27 (m, 2H), 3.84 (td, *J* = 5.7, 2.9 Hz, 2H).

^{13}C NMR (101 MHz, $CDCl_3$) δ 183.0, 181.9, 147.7, 134.7, 133.5, 132.0, 131.6, 130.5, 126.3, 126.2, 118.2, 101.6, 44.9.

Analytical data (NMR) in correspond with those reported in literature.[6]

2-(Prop-2-yn-1-ylamino)naphthalene-1,4-dione (**1n**)[1]

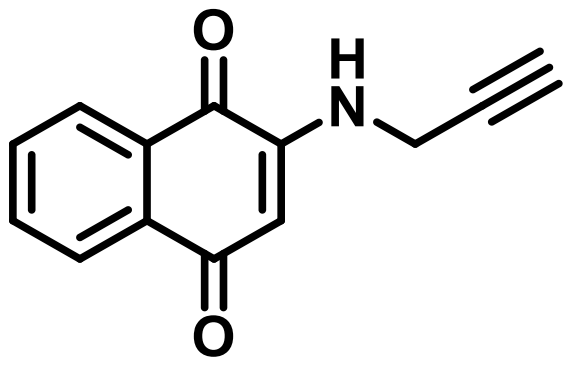

Prepared using General Procedure A on a 5 mmol scale. Purification by flash column chromatography (SiO_2, 20% EtOAc in pentane) afforded **1n** as a red solid (0.84 g, 79%).

R_f 0.3 (20% EtOAc in pentane)

^{1}H NMR (400 MHz, $CDCl_3$) δ 8.10 (ddd, *J* = 7.7, 1.4, 0.5 Hz, 1H), 8.06 (ddd, *J* = 7.7, 1.4, 0.5 Hz, 1H), 7.74 (td, *J* = 7.6, 1.4 Hz, 1H), 7.64 (td, *J* = 7.6, 1.4 Hz, 1H), 5.99 (s, 1H), 5.84 (s, 1H), 4.00 (ddd, *J* = 5.7, 2.6, 0.6 Hz, 2H), 2.35 (t, *J* = 2.5 Hz, 1H).

^{13}C NMR (101 MHz, $CDCl_3$) δ 183.1, 181.5, 147.0, 134.8, 133.3, 132.2, 130.4, 126.3, 126.3, 102.6, 73.4, 32.2.

Analytical data (NMR) in correspond with those reported in literature.[7]

2-(Benzylamino)naphthalene-1,4-dione (**1o**)[1]

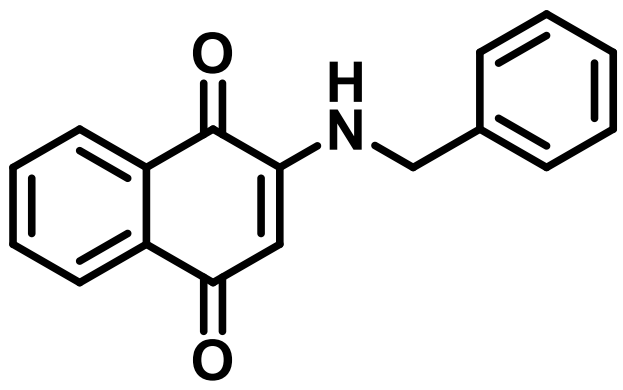

Prepared using General Procedure A on a 20 mmol scale. Purification by flash column chromatography (SiO_2, 20% EtOAc in pentane) afforded **1o** as a red solid (3.25 g, 62%).

R_f 0.3 (20% EtOAc in pentane)

^{1}H NMR (400 MHz, $CDCl_3$) δ 8.11–8.09 (m, 1H), 8.06 (dt, *J* = 7.7, 0.9 Hz, 1H), 7.73 (td, *J* = 7.6, 1.4 Hz, 1H), 7.63 (td, *J* = 7.6, 1.3 Hz, 1H), 7.40–7.36 (m, 2H), 7.35–7.31 (m, 3H), 6.20 (s, 1H), 5.79 (d, *J* = 0.7 Hz, 1H), 4.38 (d, *J* = 5.7 Hz, 2H).

^{13}C NMR (101 MHz, $CDCl_3$) δ 183.1, 181.9, 147.7, 135.9, 134.8, 133.5, 132.1, 130.5, 129.0, 128.1, 127.6, 126.3, 126.2, 101.8, 46.8.

Analytical data (NMR) in correspond with those reported in literature.[3]

2-Fluoro-3-(piperidin-1-yl)naphthalene-1,4-dione (**2a**)

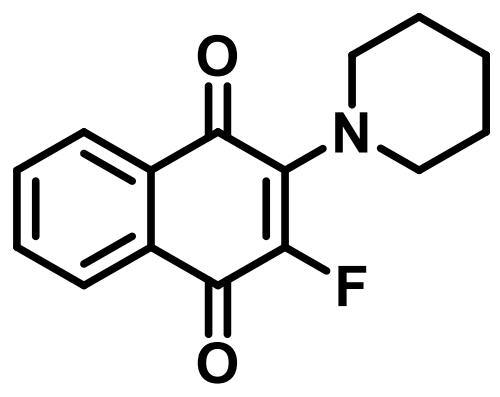

Prepared using General Procedure C. Purification by flash column chromatography (SiO_2, 10% EtOAc in pentane) afforded **2a** as a black solid (55.3 mg, 71%).

R*f* 0.6 (20% EtOAc in pentane).

M.p. 87–90 °C.

IR (film)/cm^{-1} 2923, 2853, 1671, 1651, 1604, 1572, 1338, 1186, 964, 792, 721.

^{1}H NMR (400 MHz, $CDCl_3$) δ 8.05 (dd, *J* = 7.5, 1.5 Hz, 1H), 7.96 (dd, *J* = 7.6, 1.5 Hz, 1H), 7.67 (td, *J* = 7.5, 1.5 Hz, 1H), 7.62 (td, *J* = 7.5, 1.5 Hz, 1H), 3.50–3.46 (m, 4H), 1.71 (ddq, *J* = 12.2, 7.4, 4.5 Hz, 6H).

^{13}C NMR (101 MHz, $CDCl_3$) δ 183.4 (d, *J* = 11.3 Hz), 176.0 (d, *J* = 19.3 Hz), 147.0 (d, *J* = 260.9 Hz), 138.5, 134.0, 132.9, 131.4 (d, *J* = 2.1 Hz), 130.5 (d, *J* = 3.8 Hz), 126.6, 125.6 (d, *J* = 4.0 Hz), 52.1 (d, *J* = 5.2 Hz), 26.7 (d, *J* = 1.7 Hz), 24.2 (CH_2).

^{19}F NMR (377 MHz, $CDCl_3$) δ –139.6.

HRMS (EI) m/z Calcd. for $C_{15}H_{14}NO_2F$ $[M]^+$: 259.1003, found: 259.0999.

2-(3,4-Dihydroisoquinolin-2(1*H*)-yl)-3-fluoronaphthalene-1,4-dione (**2b**)

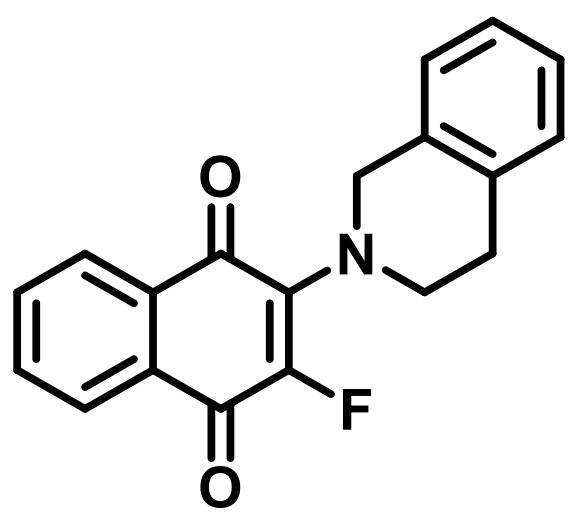

Prepared using General Procedure C. Purification by flash column chromatography (SiO_2, 10% EtOAc in pentane) afforded **2b** as a dark red gum (48.0 mg, 52%).

R*f* 0.6 (20% EtOAc in pentane)

IR (film)/cm^{-1} 2981, 1669, 1644, 1608, 1574, 1339, 1186, 963, 718.

^{1}H NMR (400 MHz, $CDCl_3$) δ 8.09–8.07 (m, 1H), 8.02–8.00 (m, 1H), 7.68 (dtd, J = 21.0, 7.5, 1.5 Hz, 3H), 7.21–7.17 (m, 3H), 7.09–7.07 (m, 1H), 4.79 (dd, J = 3.0, 1.1 Hz, 2H), 3.80 (td, J = 5.8, 1.8 Hz, 2H), 3.13 (t, J = 5.8 Hz, 2H).

^{13}C NMR (101 MHz, CDCl$_3$) δ 183.5 (d, *J* = 11.0 Hz), 176.0 (d, *J* = 19.2 Hz), 146.7 (d, *J* = 261.1 Hz), 137.8, 134.2, 134.1, 133.5 (d, *J* = 2.1 Hz), 133.0, 131.2 (d, *J* = 2.0 Hz), 130.4 (d, *J* = 4.0 Hz), 129.0, 126.2, 126.1, 125.7 (d, *J* = 4.0 Hz), 52.7 (d, *J* = 6.6 Hz), 48.8 (d, *J* = 4.1 Hz), 29.5 (d, *J* = 2.0 Hz).

^{19}F NMR (377 MHz, CDCl$_3$) δ –140.1.

HRMS (EI) m/z Calcd. for $C_{19}H_{15}FNO_2$ [M+H]$^+$: 308.1081, found: 308.1075.

Note: The compound undergoes slow degradation over time upon standing; characterization data were acquired on the freshly isolated material immediately following purification.

2-Fluoro-3-(4-phenylpiperidin-1-yl)naphthalene-1,4-dione (**2c**)

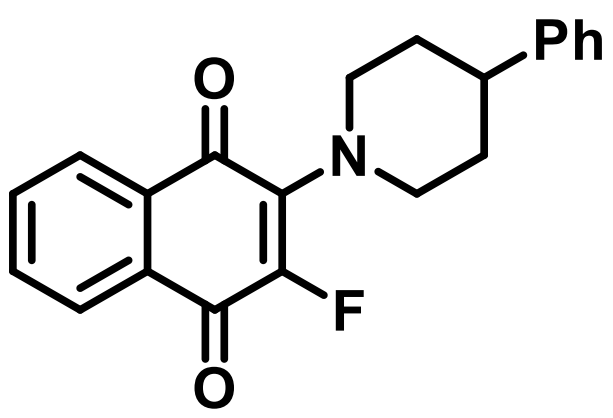

Prepared using General Procedure C. Purification by flash column chromatography (SiO_2, 10% EtOAc in pentane) afforded **2c** as a dark red solid (38.0 mg, 38%).

R*f* 0.65 (20% EtOAc in pentane)

M.p. 95–98 °C.

IR (film)/cm^{-1} 2981, 1652, 1615, 1575, 1335, 1151, 963, 764, 704.

^{1}H NMR (400 MHz, CDCl$_3$) δ 8.10–8.08 (m, 1H), 8.02–8.00 (m, 1H), 7.71 (td, *J* = 7.5, 1.5 Hz, 1H), 7.66 (td, *J* = 7.5, 1.5 Hz, 1H), 7.35–7.31 (m, 2H), 7.27–7.21 (m, 3H), 3.99–3.95 (m, 2H), 3.35–3.28 (m, 2H), 2.80 (ddd, *J* = 15.9, 9.5, 7.3 Hz, 1H), 1.99–1.93 (m, 4H).

^{13}C NMR (101 MHz, CDCl$_3$) δ 183.5 (d, *J* = 11.1 Hz), 176.2 (d, *J* = 19.4 Hz), 147.4 (d, *J* = 262.3 Hz), 145.3, 138.3, 134.1, 133.0, 131.4 (d, *J* = 2.1 Hz), 130.5 (d, *J* = 4.0 Hz), 128.6, 126.8, 126.7, 126.5, 125.7 (d, *J* = 3.8 Hz), 51.7 (d, *J* = 5.2 Hz), 42.4, 34.0 (d, *J* = 1.5 Hz).

^{19}F NMR (377 MHz, CDCl$_3$) δ –138.8.

HRMS (EI) m/z Calcd. for $C_{21}H_{19}FNO_2$ [M+H]$^+$: 336.1394, found: 336.1393.

tert-Butyl 4-(3-fluoro-1,4-dioxo-1,4-dihydronaphthalen-2-yl)piperazine-1-carboxylate (**2d**)

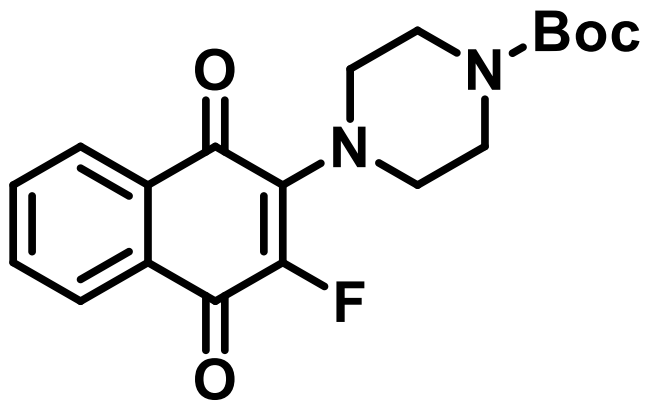

Prepared using General Procedure C. Purification by flash column chromatography (SiO_2, 10% EtOAc in pentane) afforded **2d** as a black solid (71.4 mg, 66%).

R*f* 0.5 (20% EtOAc in pentane)

M.p. 98–101 °C.

IR (film)/cm^{-1} 2922, 2853, 1703, 1666, 1651, 1608, 1421, 1343, 1154, 955, 720.

^{1}H NMR (400 MHz, $CDCl_3$) δ 8.08–8.05 (m, 1H), 7.99 (dd, *J* = 7.4, 1.6 Hz, 1H), 7.70 (td, *J* = 7.5, 1.6 Hz, 1H), 7.66 (td, *J* = 7.5, 1.6 Hz, 1H), 3.62–3.54 (m, 4H), 3.52–3.45 (m, 4H), 1.48 (s, 9H).

^{13}C NMR (101 MHz, $CDCl_3$) δ 183.1 (d, *J* = 11.0 Hz), 176.3 (d, *J* = 19.6 Hz), 154.6, 147.5 (d, *J* = 264.2 Hz), 137.4, 134.2, 133.3, 131.2 (d, *J* = 2.0 Hz), 130.2 (d, *J* = 3.9 Hz), 126.7, 125.8 (d, *J* = 3.7 Hz), 80.2, 50.5, 50.4, 28.4.

^{19}F NMR (377 MHz, $CDCl_3$) δ –137.8.

HRMS (ESI) m/z Calcd. for $C_{19}H_{22}FN_2O_4$ $[M+H]^+$: 361.1558, found: 361.1560.

2-Fluoro-3-morpholinonaphthalene-1,4-dione (**2e**)

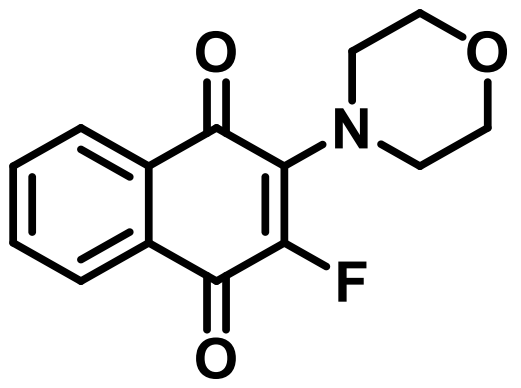

Prepared using General Procedure C. Purification by flash column chromatography (SiO_2, 10% EtOAc in pentane) afforded **2e** as a black solid (61.2 mg, 77%).

R*f* 0.6 (30% EtOAc in pentane)

M.p. 161–163 °C.

IR (film)/cm^{-1} 2980, 1671, 1651, 1606, 1575, 1339, 1257, 1113, 969, 723.

^{1}H NMR (400 MHz, $CDCl_3$) δ 8.07–8.04 (m, 1H), 7.99–7.96 (m, 1H), 7.70 (td, *J* = 7.5, 1.6 Hz, 1H), 7.65 (td, *J* = 7.5, 1.6 Hz, 1H), 3.84–3.82 (m, 4H), 3.58–3.55 (m, 4H).

^{13}C NMR (101 MHz, CDCl$_3$) δ 183.1 (d, *J* = 10.9 Hz), 176.2 (d, *J* = 19.6 Hz), 147.3 (d, *J* = 264.1 Hz), 137.2, 134.2 (d, *J* = 1.4 Hz), 133.2, 131.2 (d, *J* = 2.1 Hz), 130.2 (d, *J* = 4.0 Hz), 126.7, 125.7 (d, *J* = 3.8 Hz), 67.3 (d, *J* = 1.9 Hz), 51.0 (d, *J* = 5.0 Hz).

^{19}F NMR (377 MHz, CDCl$_3$) δ –138.5.

HRMS (ESI) m/z Calcd. for $C_{14}H_{13}FNO_3$ [M+H]$^+$: 262.0874, found: 262.0872.

2-(4,4-Difluoropiperidin-1-yl)-3-fluoronaphthalene-1,4-dione (**2f**)

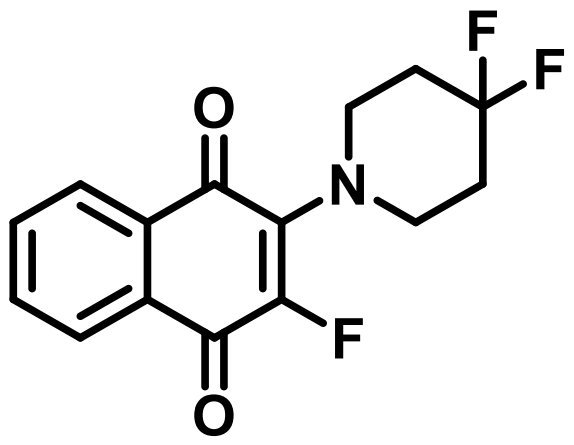

Prepared using General Procedure C. Purification by flash column chromatography (SiO_2, 10% EtOAc in pentane) afforded **2f** as a red solid (71.0 mg, 85%).

R*f* 0.6 (20% EtOAc in pentane).

M.p. 152–155 °C.

IR (film)/cm^{-1} 2981, 1618, 1337, 1243, 1117, 932, 720, 666.

^{1}H NMR (400 MHz, CDCl$_3$) δ 8.08–8.06 (m, 1H), 8.01–7.99 (m, 1H), 7.74–7.69 (m, 1H), 7.69–7.65 (m, 1H), 3.62–3.59 (m, 4H), 2.21–2.11 (m, 4H).

^{13}C NMR (101 MHz, CDCl$_3$) δ 183.1 (d, *J* = 10.7 Hz), 176.4 (d, *J* = 19.9 Hz), 148.0 (d, *J* = 265.4 Hz), 137.2, 134.3 (d, *J* = 1.4 Hz), 133.4, 131.2 (d, *J* = 2.1 Hz), 130.2 (d, *J* = 4.0 Hz), 126.8, 125.8 (d, *J* = 3.9 Hz), 122.4 (d, *J* = 241.6 Hz), 47.6 (q, *J* = 5.5 Hz), 34.8 (td, *J* = 23.0, 1.7 Hz).

^{19}F NMR (377 MHz, CDCl$_3$) δ –97.5, –136.7.

HRMS (EI) m/z Calcd. for $C_{15}H_{13}F_3NO_2$ [M+H]$^+$: 296.0893, found: 296.0893.

2-Fluoro-3-(4-hydroxypiperidin-1-yl)naphthalene-1,4-dione (**2g**)

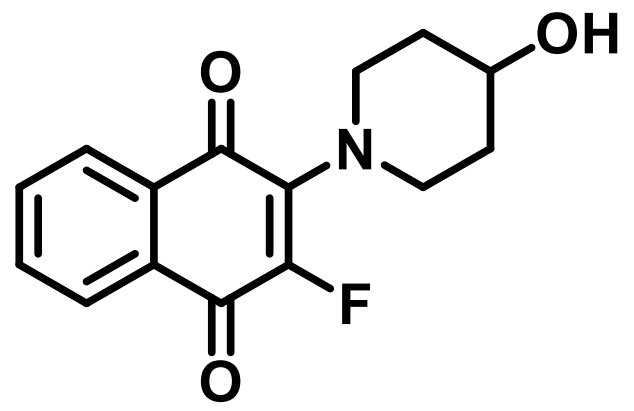

Prepared using General Procedure C. Purification by flash column chromatography (SiO_2, 50% EtOAc in pentane) afforded **2g** as a red solid (56.6 mg, 73%).

R*f* 0.1 (50% EtOAc in pentane).

M.p. 121–124 °C.

IR (film)/cm^{-1} 2981, 1653, 1617, 1341, 1239, 1188, 941, 716.

^{1}H NMR (400 MHz, $CDCl_3$) δ 8.06 (dd, *J* = 7.3, 1.7 Hz, 1H), 7.98 (dd, *J* = 7.6, 1.4 Hz, 1H), 7.69 (td, *J* = 7.5, 1.6 Hz, 1H), 7.64 (td, *J* = 7.5, 1.5 Hz, 1H), 3.97 (tt, *J* = 8.2, 3.9 Hz, 1H), 3.82–3.76 (m, 2H), 3.36–3.29 (m, 2H), 2.08–2.01 (m, 2H), 1.83 (s, 1H), 1.73 (dtd, *J* = 12.8, 8.8, 3.8 Hz, 2H).

^{13}C NMR (101 MHz, $CDCl_3$) δ 183.3 (d, *J* = 11.1 Hz), 176.2 (d, *J* = 19.3 Hz), 147.3 (d, *J* = 262.3 Hz), 138.1, 134.1, 133.1, 131.3 (d, *J* = 2.1 Hz), 130.4 (d, *J* = 4.0 Hz), 126.7, 125.7 (d, *J* = 3.9 Hz), 66.9, 48.3 (d, *J* = 5.4 Hz), 35.0 (d, *J* = 1.5 Hz).

^{19}F NMR (377 MHz, $CDCl_3$) δ –138.6.

HRMS (EI) m/z Calcd. for $C_{15}H_{15}FNO_3$ $[M+H]^+$: 276.1030, found: 276.1026.

2-(Cyclohexylamino)-3-fluoronaphthalene-1,4-dione (**2h**)

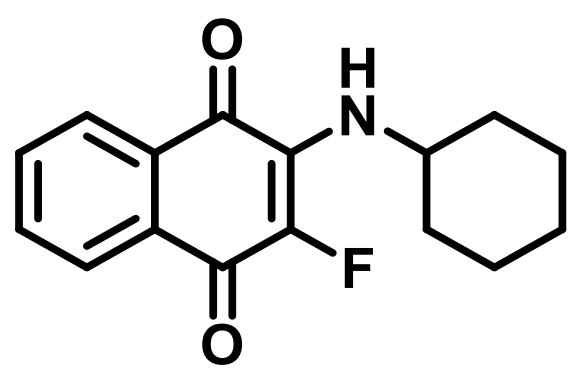

Prepared using General Procedure C. Purification by flash column chromatography (SiO_2, 5% EtOAc in pentane) afforded **2h** as a red solid (41.0 mg, 54%).

R*f* 0.6 (10% EtOAc in pentane).

M.p. 144–146 °C.

IR (film)/cm^{-1} 3326, 2981, 2923, 1674, 1609, 1571, 1505, 1362, 1270, 1081, 943, 720.

^{1}H NMR (400 MHz, $CDCl_3$) δ 8.08 (dd, *J* = 7.7, 1.3 Hz, 1H), 7.98 (dd, *J* = 7.7, 1.3 Hz, 1H), 7.70 (td, *J* = 7.5, 1.3 Hz, 1H), 7.59 (td, *J* = 7.6, 1.3 Hz, 1H), 5.42–5.39 (m, 1H), 3.77 (dddd, *J* = 13.0,

10.9, 6.6, 2.8 Hz, 1H), 2.07–2.02 (m, 2H), 1.77 (dp, *J* = 11.4, 3.8 Hz, 2H), 1.64 (dq, *J* = 11.9, 3.8 Hz, 1H), 1.44–1.15 (m, 5H).

^{13}C NMR (101 MHz, $CDCl_3$) δ 182.5 (d, *J* = 12.5 Hz), 175.1 (d, *J* = 17.3 Hz), 140.7 (d, *J* = 251.4 Hz), 134.9 (d, *J* = 1.5 Hz), 132.6 (d, *J* = 1.7 Hz), 132.3, 131.5 (d, *J* = 3.7 Hz), 129.6 (d, *J* = 1.7 Hz), 126.3, 126.0 (d, *J* = 4.1 Hz), 52.8 (d, *J* = 7.3 Hz), 34.1 (d, *J* = 2.2 Hz), 25.3, 24.5.

^{19}F NMR (377 MHz, $CDCl_3$) δ –156.7.

HRMS (ESI) m/z Calcd. for $C_{16}H_{17}FNO_2$ $[M+H]^+$: 274.1238, found: 274.1235.

2-Fluoro-3-(phenylamino)naphthalene-1,4-dione (**2i**)

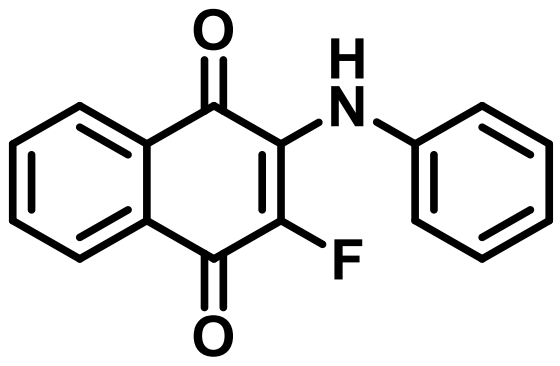

Prepared using General Procedure C. Purification by flash column chromatography (SiO_2, 10% EtOAc in pentane) afforded **2i** as a black solid (24.0 mg, 45%).

R*f* 0.5 (20% EtOAc in pentane).

M.p. 206–208 °C.

IR (film)/cm^{-1} 3306, 2922, 1666, 1625, 1574, 1446, 1250, 965, 718.

^{1}H NMR (500 MHz, $CDCl_3$) δ 8.17–8.15 (m, 1H), 8.11 (dd, J = 7.7, 1.3 Hz, 1H), 7.78 (td, J = 7.6, 1.3 Hz, 1H), 7.69 (td, J = 7.6, 1.3 Hz, 1H), 7.36 (dd, J = 8.5, 7.4 Hz, 2H), 7.20–7.11 (m, 4H).

^{13}C NMR (126 MHz, $CDCl_3$) δ 182.4 (d, *J* = 11.7 Hz), 175.9 (d, *J* = 18.2 Hz), 142.1 (d, *J* = 265.3 Hz), 137.8 (d, *J* = 1.6 Hz), 135.1 (d, *J* = 1.4 Hz), 133.0, 131.2 (d, *J* = 3.3 Hz), 129.7 (d, *J* = 1.8 Hz), 129.4 (d, *J* = 1.5 Hz), 128.8, 126.7, 126.4 (d, *J* = 3.6 Hz), 125.1, 122.4 (d, *J* = 5.0 Hz).

^{19}F NMR (377 MHz, $CDCl_3$) δ –133.6.

HRMS (EI) m/z Calcd. for $C_{16}H_{11}FNO_2$ $[M+H]^+$: 268.0768, found: 268.0774.

2-Fluoro-3-((4-methoxyphenyl)amino)naphthalene-1,4-dione (**2j**)

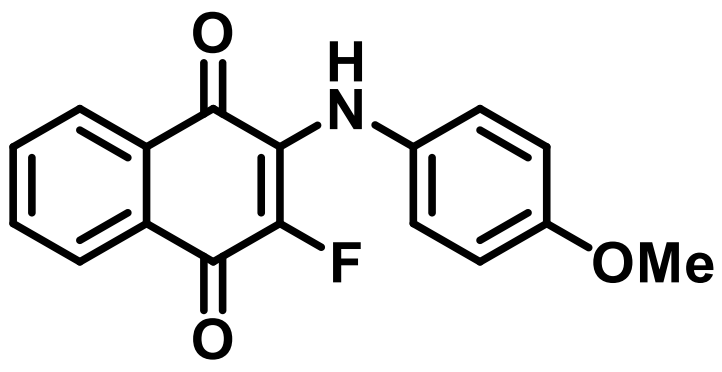

Prepared using General Procedure C. Purification by flash column chromatography (SiO_2, 10% EtOAc in pentane) afforded **2j** as a black solid (32.3 mg, 36%).

R*f* 0.45 (20% EtOAc in pentane).

M.p. 171–174 °C.

IR (film)/cm^{-1} 3307, 2981, 1666, 1630, 1508, 1351, 1239, 1170, 966, 717.

^{1}H NMR (500 MHz, $CDCl_3$) δ 8.14 (dt, *J* = 7.8, 0.9 Hz, 1H), 8.10–8.08 (m, 1H), 7.76 (td, *J* = 7.6, 1.4 Hz, 1H), 7.67 (td, *J* = 7.6, 1.3 Hz, 1H), 7.10–7.06 (m, 3H), 6.89–6.86 (m, 2H), 3.82 (s, 3H).

^{13}C NMR (126 MHz, $CDCl_3$) δ 182.5 (d, *J* = 11.6 Hz), 175.6 (d, *J* = 17.7 Hz), 157.4, 141.7 (d, *J* = 262.7 Hz), 135.1 (d, *J* = 1.4 Hz), 132.8, 131.3 (d, *J* = 3.3 Hz), 130.7 (d, *J* = 1.5 Hz), 130.2, 129.6 (d, *J* = 1.7 Hz), 126.6, 126.3 (d, *J* = 3.7 Hz), 124.4 (d, *J* = 4.7 Hz), 55.5.

^{19}F NMR (377 MHz, $CDCl_3$) δ –138.0.

HRMS (EI) m/z Calcd. for $C_{17}H_{13}FNO_3$ $[M+H]^+$: 298.0874, found: 298.0875.

2-Fluoro-3-((4-(trifluoromethyl)phenyl)amino)naphthalene-1,4-dione (**2k**)

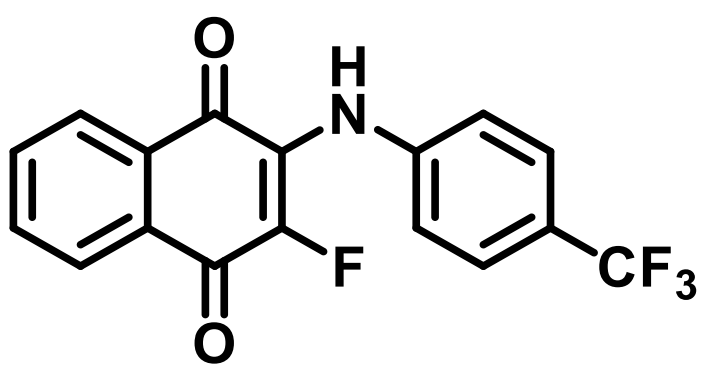

Prepared using General Procedure C. Purification by flash column chromatography (SiO_2, 10% EtOAc in pentane) afforded **2k** as a red solid (39.6 mg, 39%).

R*f* 0.45 (20% EtOAc in pentane).

M.p. 189–192 °C.

IR (film)/cm^{-1} 3253, 2957, 2922, 1728, 1682, 1626, 1609, 1578, 1523, 1503, 1285, 1110, 1067, 966, 713.

^{1}H NMR (400 MHz, $CDCl_3$) δ 8.19–8.16 (m, 1H), 8.14 (dd, *J* = 7.7, 1.3 Hz, 1H), 7.81 (td, *J* = 7.6, 1.4 Hz, 1H), 7.73 (td, *J* = 7.6, 1.4 Hz, 1H), 7.61 (d, *J* = 8.4 Hz, 2H), 7.21 (s, 1H), 7.17 (dd, *J* = 8.6, 4.0 Hz, 2H).

^{13}C NMR (126 MHz, CDCl$_3$) δ 182.1 (d, *J* = 11.5 Hz), 176.1 (d, *J* = 18.7 Hz), 142.8 (d, *J* = 269.0 Hz), 141.0, 135.3 (d, *J* = 1.4 Hz), 133.4, 130.9 (d, *J* = 3.4 Hz), 129.6 (d, *J* = 1.7 Hz), 128.3, 126.9, 126.6 (d, *J* = 3.6 Hz), 126.1 (q, *J* = 3.8 Hz), 124.0 (q, *J* = 271.6 Hz), 121.4 (d, *J* = 5.5 Hz).

^{19}F NMR (377 MHz, CDCl$_3$) δ –62.1, –129.1.

HRMS (EI) m/z Calcd. for $C_{17}H_{10}F_4NO_2$ [M+H]$^+$: 336.0642, found: 336.0629.

2-((4-Cyclohexylphenyl)amino)-3-fluoronaphthalene-1,4-dione (**2l**)

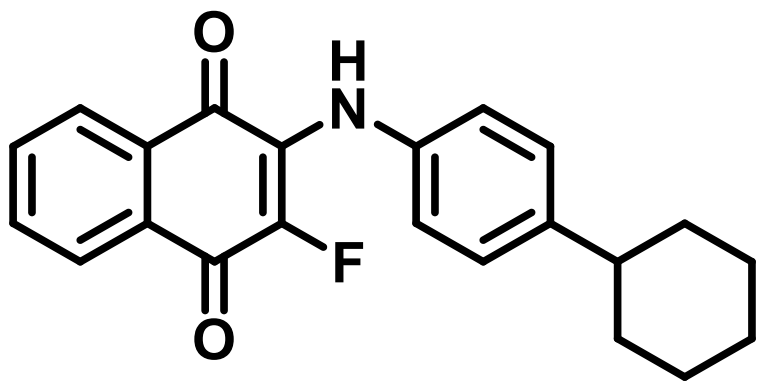

Prepared using General Procedure C. Purification by flash column chromatography (SiO_2, 10% EtOAc in pentane) afforded **2l** as a black solid (43.2 mg, 41%).

R*f* 0.6 (20% EtOAc in pentane)

M.p. 189–191 °C.

IR (film)/cm^{-1} 3307, 2981, 2918, 1651, 1622, 1575, 1521, 1253, 968, 717.

^{1}H NMR (500 MHz, CDCl$_3$) δ 8.15 (dd, *J* = 7.7, 1.3 Hz, 1H), 8.10 (dd, *J* = 7.6, 1.3 Hz, 1H), 7.76 (td, *J* = 7.5, 1.3 Hz, 1H), 7.68 (td, *J* = 7.6, 1.3 Hz, 1H), 7.19–7.17 (m, 2H), 7.14 (d, *J* = 3.4 Hz, 1H), 7.05 (dd, *J* = 8.4, 3.7 Hz, 2H), 2.50 (tt, *J* = 11.5, 3.6 Hz, 1H), 1.91–1.83 (m, 4H), 1.75 (dtt, *J* = 12.9, 3.2, 1.6 Hz, 1H), 1.45–1.35 (m, 4H), 1.30–1.21 (m, 1H).

^{13}C NMR (126 MHz, CDCl$_3$) δ 182.5 (d, *J* = 11.7 Hz), 175.7 (d, *J* = 17.9 Hz), 145.3, 141.9 (d, *J* = 264.1 Hz), 135.4 (d, *J* = 1.5 Hz), 135.1 (d, *J* = 1.4 Hz), 132.9, 131.3 (d, *J* = 3.3 Hz), 129.6 (d, *J* = 1.5 Hz), 127.1, 126.7, 126.3 (d, *J* = 3.6 Hz), 122.4 (d, *J* = 5.0 Hz), 44.0, 34.4, 26.8, 26.1.

^{19}F NMR (377 MHz, CDCl$_3$) δ –135.1.

HRMS (EI) m/z Calcd. for $C_{22}H_{21}FNO_2$ [M+H]$^+$: 350.1551, found: 350.1554.

2-(Allylamino)-3-fluoronaphthalene-1,4-dione (**2m**)

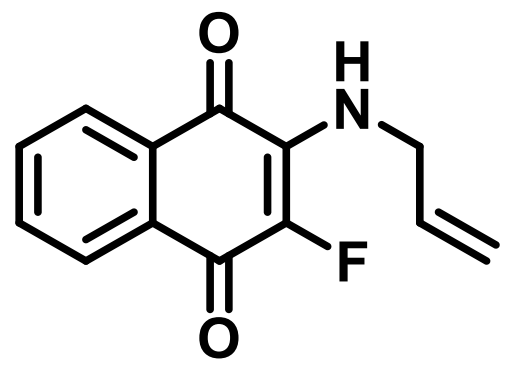

Prepared using General Procedure C. Purification by flash column chromatography (SiO_2, 5% EtOAc in pentane) afforded **2m** as a red solid (31.3 mg, 45%).

R*f* 0.6 (10% EtOAc in pentane).

M.p. 112–115 °C.

IR (film)/cm^{-1} 3322, 2980, 2924, 1678, 1610, 1692, 1569, 1495, 1360, 1182, 934, 719, 657.

^{1}H NMR (400 MHz, $CDCl_3$) δ 8.09 (dd, *J* = 7.6, 1.3 Hz, 1H), 8.01 (dd, *J* = 7.7, 1.3 Hz, 1H), 7.72 (td, *J* = 7.6, 1.3 Hz, 1H), 7.62 (td, *J* = 7.6, 1.3 Hz, 1H), 5.94 (ddt, *J* = 15.3, 10.7, 5.6 Hz, 1H), 5.56 (s, 1H), 5.31-5.26 (m, 2H), 4.17 (tq, *J* = 6.0, 1.6 Hz, 2H).

^{13}C NMR (101 MHz, $CDCl_3$) δ 182.3 (d, *J* = 12.3 Hz), 175.4 (d, *J* = 17.6 Hz), 141.4 (d, *J* = 253.4 Hz), 135.0 (d, *J* = 1.5 Hz), 133.7 (d, *J* = 2.5 Hz), 133.3, 132.5, 131.3 (d, *J* = 3.7 Hz), 129.5 (d, *J* = 1.7 Hz), 126.4, 126.2 (d, *J* = 4.0 Hz), 117.4, 46.6 (d, *J* = 7.9 Hz).

^{19}F NMR (377 MHz, $CDCl_3$) δ –155.7.

HRMS (EI) m/z Calcd. for $C_{13}H_{11}FNO_2$ $[M+H]^+$: 232.0768, found: 232.0767.

2-Fluoro-3-(prop-2-yn-1-ylamino)naphthalene-1,4-dione (**2n**)

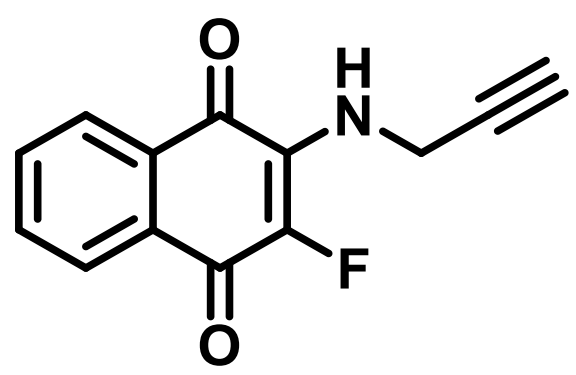

Prepared using General Procedure C. Purification by flash column chromatography (SiO_2, 10% EtOAc in pentane) afforded **2n** as a red solid (14.4 mg, 22%).

R*f* 0.4 (20% EtOAc in pentane).

M.p. 187–189 °C.

IR (film)/cm^{-1} 2981, 1669, 1644, 1609, 1574, 1339, 1186, 963, 719.

^{1}H NMR (400 MHz, $CDCl_3$) δ 8.12 (dd, *J* = 7.7, 1.4 Hz, 1H), 8.03 (dd, *J* = 7.7, 1.3 Hz, 1H), 7.74 (td, *J* = 7.5, 1.3 Hz, 1H), 7.65 (td, *J* = 7.5, 1.3 Hz, 1H), 5.50 (s, 1H), 4.33 (dt, *J* = 6.1, 2.4 Hz, 2H), 2.37 (t, *J* = 2.5 Hz, 1H).

^{13}C NMR (126 MHz, $CDCl_3$) δ 181.9 (d, *J* = 12.0 Hz), 175.7 (d, *J* = 17.9 Hz), 142.0 (d, *J* = 255.9 Hz), 135.0 (d, *J* = 1.5 Hz), 132.8, 132.4 (d, *J* = 1.4 Hz), 131.1 (d, *J* = 3.6 Hz), 129.6 (d, *J* = 1.7 Hz), 126.5, 126.3 (d, *J* = 4.0 Hz), 78.9 (d, *J* = 2.8 Hz), 73.2, 34.3 (d, *J* = 9.7 Hz).

^{19}F NMR (377 MHz, $CDCl_3$) δ –154.0.

HRMS (EI) m/z Calcd. for $C_{13}H_9FNO_2$ $[M+H]^+$: 230.0612, found: 230.0602.

2-Amino-3-fluoronaphthalene-1,4-dione (**2p**)

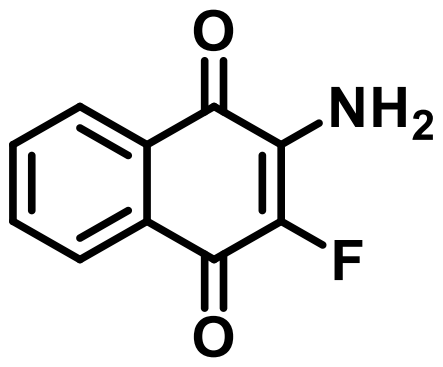

Prepared using a modified procedure from General Procedure C with Selectfluor (2.1 equiv). Purification by flash column chromatography (SiO_2, 20% EtOAc in pentane) afforded **2p** as a black solid (13.5 mg, 35%).

R_f 0.3 (20% EtOAc in pentane).

M.p. 186–188 °C.

IR (film)/cm^{-1} 2981, 1686, 1651, 1620, 1585, 1563, 1369, 1180, 947, 719.

^{1}H NMR (500 MHz, CD_3OD) δ 8.04–8.01 (m, 2H), 7.77 (td, *J* = 7.6, 1.4 Hz, 1H), 7.69 (td, *J* = 7.6, 1.3 Hz, 1H).

^{13}C NMR (126 MHz, CD_3OD) δ 182.9 (d, *J* = 10.9 Hz), 176.0 (d, *J* = 15.5 Hz), 143.1 (d, *J* = 250.1 Hz), 137.8 (d, *J* = 5.5 Hz), 135.9 (d, *J* = 1.4 Hz), 133.8, 132.6 (d, *J* = 4.3 Hz), 131.4 (d, *J* = 1.9 Hz), 127.2, 126.7 (d, *J* = 4.1 Hz).

^{19}F NMR (377 MHz, CD_3OD) δ –156.9.

HRMS (EI) m/z Calcd. for $C_{10}H_7FNO_2$ $[M+H]^+$: 192.0455, found: 192.0458.

Note: The NH_2 signal was not observed in the ^{1}H NMR spectrum due to the rapid H/D exchange with the CD_3OD solvent.

Computational methods

Computational investigations were carried out using ORCA 6.1.[8] Geometry optimisations and frequency calculations were carried out with the M06-2X functional[9] developed by the Grimme group, Ahlrich's def2-SVP basis set[10,11] and the D3(0) dispersion correction developed by the Grimme group.[12] Solvation of solute structures (Selectfluor, quinone, RC, TS_{ionic}, TS_{ISET} and PROD) with acetonitrile solvent was modelled with Cramer and Truhlar's SMD solvation model.[13] The RIJCOSX approximation was utilised for all calculations by using the `rijcosx` keyword.[14,15] The DFT integration grid setting was set to `defgrid3`. The convergence setting for geometry optimisations was set to `tight`. For transition state geometry optimisations, `maxstep 0.1` was set in the `%geom` block. Free energy changes were calculated assuming the quasi-rigid rotor and harmonic oscillator (RRHO) approximations of Grimme[16] with the default cut-off frequency (1.00 cm^{-1}).

Symmetry-broken/spin-polarised/spin-broken wavefunctions were generated for open-shell singlet states at the DFT level of theory by using the `flipspin` functionality in the `%scf` input block, although it was found that merely invoking the unrestricted Kohn-Sham (`! uks`) formalism whilst enforcing a singlet spin multiplicity successfully led to the same spin-broken wavefunction solution as the `flipspin` functionality.

Cartesian coordinates

Selectfluor

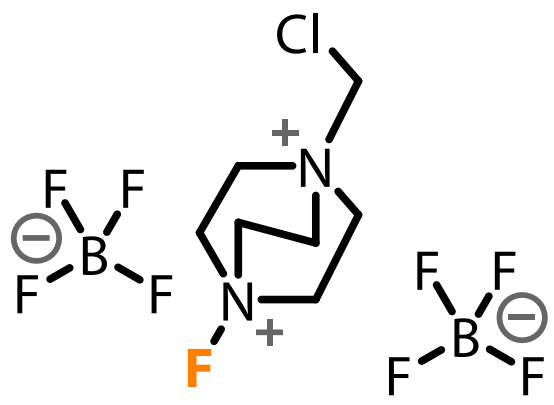

N	0.866783	-1.106076	0.819004
C	1.920010	-2.018450	0.284146
C	0.910367	-1.039555	2.305524
C	0.966698	0.256415	0.227806
C	2.343689	0.816691	0.605150
C	2.294843	-0.505624	2.702433
C	3.291823	-1.436809	0.668222
H	1.738518	-2.995636	0.744115
H	1.778293	-2.056580	-0.801926
H	0.846369	0.147687	-0.856290
H	0.154650	0.845520	0.666240
H	0.109247	-0.360933	2.615985
H	0.737766	-2.057869	2.668236
H	3.854152	-2.138551	1.291607
H	3.864479	-1.167746	-0.225492
H	2.953613	0.969116	-0.290473
H	2.253578	1.743965	1.179252
H	2.211555	0.409945	3.297693
H	2.858858	-1.265440	3.252108
N	3.087219	-0.178501	1.464836
C	4.383839	0.485794	1.821408
H	4.909528	0.706239	0.885963
H	4.131134	1.400849	2.370205
Cl	5.404189	-0.539789	2.832318
F	-0.343369	-1.624951	0.464183
F	0.861531	0.154153	-3.073250
F	2.838416	-0.402024	-2.077667
F	1.509986	-2.033636	-2.960242
F	2.615908	-0.585573	-4.345938
F	2.068306	2.225835	4.406774
F	2.229398	3.460498	2.492731
F	0.553812	1.923647	2.723372
F	0.515089	3.847641	3.959776
B	1.340508	2.880576	3.405891
B	1.952648	-0.717729	-3.134177

Quinone

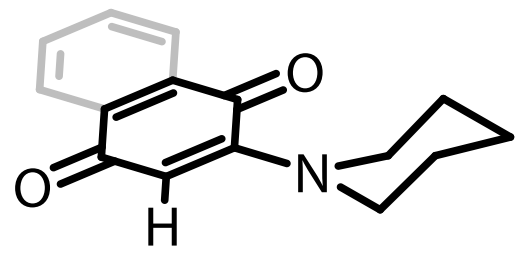

C	-2.955499	-2.345899	1.653090
C	-2.684655	-0.922091	-0.378855
H	-3.129267	-3.022234	-0.382784
O	-3.187095	-3.441726	2.136801
C	-2.905935	-2.136856	0.211572
C	-2.768788	-1.151218	2.538605
C	-2.289422	0.248600	0.506835
C	-2.482967	0.100187	1.978003
C	-2.890736	-1.282913	3.921708
H	-3.109960	-2.267716	4.336579
C	-2.314673	1.215723	2.802603
H	-2.080212	2.180014	2.349460
C	-2.448010	1.082194	4.183223
H	-2.324769	1.953534	4.828298
C	-2.733651	-0.166675	4.742347
H	-2.832798	-0.268474	5.824364
O	-1.779694	1.242606	0.042625
N	-2.629386	-0.721557	-1.724358
C	-2.589004	-1.870864	-2.622429
H	-3.618211	-2.213122	-2.847360
H	-2.067223	-2.693252	-2.118327
C	-3.228928	0.471139	-2.340121
H	-3.266980	1.284807	-1.613301
H	-4.270145	0.216769	-2.617601
C	-1.848262	-1.518807	-3.911156
H	-0.788254	-1.344416	-3.663926
H	-1.888196	-2.387104	-4.585172
C	-2.465807	0.882320	-3.590175
H	-1.442764	1.173505	-3.300405
H	-2.951372	1.764784	-4.032157
C	-2.422293	-0.275407	-4.583986
H	-3.447521	-0.487685	-4.932835
H	-1.827573	-0.009966	-5.470444

RC

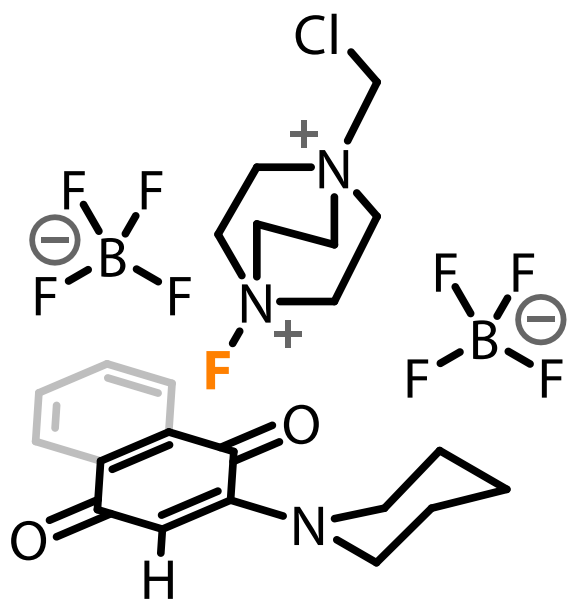

N	0.722929	-0.986193	0.369068
C	1.789986	-1.892510	-0.147249
C	0.754356	-0.891524	1.854263
C	0.802106	0.368991	-0.242543
C	2.172140	0.953630	0.122652
C	2.135662	-0.341118	2.243421
C	3.152094	-1.279719	0.219973
H	1.624289	-2.861832	0.334966
H	1.642084	-1.956601	-1.231428
H	0.679813	0.237758	-1.324068
H	-0.017209	0.955133	0.189213
H	-0.055415	-0.211074	2.146146
H	0.586182	-1.903722	2.237037
H	3.732464	-1.963391	0.846838
H	3.713570	-1.008611	-0.680457
H	2.780563	1.094702	-0.775868
H	2.068804	1.891082	0.677177
H	2.045285	0.577812	2.831984
H	2.708078	-1.092272	2.796319
N	2.925827	-0.016221	1.002041
C	4.211998	0.671456	1.352954
H	4.735810	0.887320	0.415360
H	3.945759	1.589529	1.890190
Cl	5.244572	-0.326637	2.378786
F	-0.477314	-1.534839	0.008940
C	-3.325984	-2.204982	1.009800
C	-3.224967	-0.856289	-1.091756
H	-3.729395	-2.939795	-0.970256
O	-3.530133	-3.277474	1.558127
C	-3.432598	-2.041380	-0.429950
C	-2.988268	-0.994060	1.827396
C	-2.733585	0.336845	-0.287747
C	-2.756835	0.237391	1.198216
C	-2.897228	-1.091589	3.214257
H	-3.072477	-2.060963	3.682954
C	-2.441461	1.365505	1.958142

H	-2.245149	2.311516	1.452390
C	-2.360786	1.265965	3.346728
H	-2.103793	2.146698	3.937382
C	-2.583303	0.037821	3.972888
H	-2.509591	-0.040484	5.058938
O	-2.243045	1.300471	-0.834830
N	-3.248001	-0.720740	-2.437472
C	-3.121282	-1.867698	-3.330628
H	-4.103619	-2.101599	-3.780367
H	-2.796375	-2.741185	-2.755291
C	-3.651491	0.503043	-3.136331
H	-3.824896	1.307126	-2.418866
H	-4.614709	0.285039	-3.634233
C	-2.089096	-1.556272	-4.416122
H	-1.103197	-1.456559	-3.935867
H	-2.037036	-2.410454	-5.107948
C	-2.619363	0.888547	-4.186984
H	-1.670844	1.119951	-3.678664
H	-2.955065	1.794922	-4.712676
C	-2.415753	-0.266601	-5.166973
H	-3.339857	-0.408082	-5.753776
H	-1.613081	-0.030988	-5.882119
F	0.658579	0.262684	-3.556392
F	2.641060	-0.289170	-2.565238
F	1.279638	-1.928675	-3.380242
F	2.386284	-0.540519	-4.826931
F	1.931002	2.419388	3.907274
F	2.077296	3.660294	1.995666
F	0.438159	2.085821	2.210210
F	0.339437	4.001907	3.452363
B	1.196376	3.058775	2.901760
B	1.741820	-0.625518	-3.601937

TS$_{ionic}$

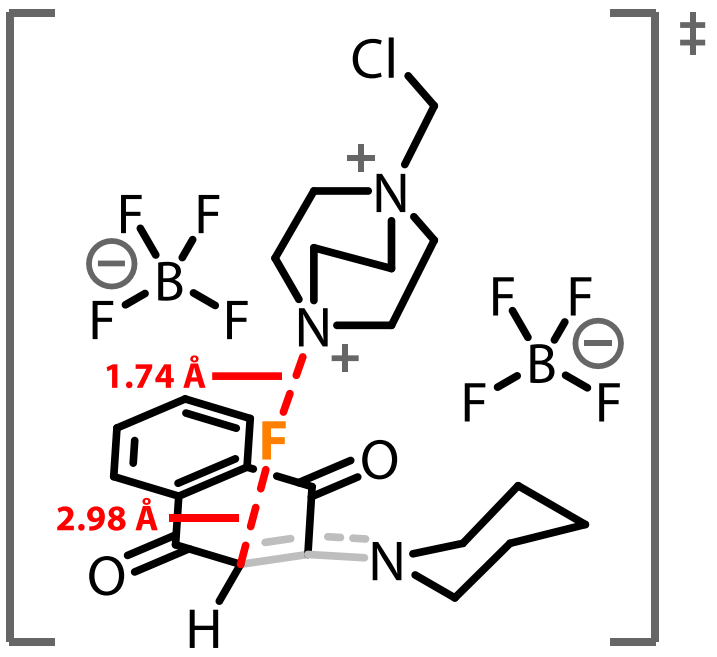

N	0.947085	-1.096208	0.822151
C	1.933573	-2.013334	0.270535
C	0.965029	-1.061368	2.275341
C	1.022551	0.234822	0.242638
C	2.399437	0.817822	0.624019
C	2.355531	-0.531697	2.696959
C	3.332496	-1.450151	0.631915
H	1.772816	-2.999165	0.720327
H	1.798495	-2.055990	-0.815573
H	0.913464	0.149086	-0.844159
H	0.219649	0.843341	0.674222
H	0.170972	-0.384003	2.613654
H	0.794215	-2.077358	2.649274
H	3.909528	-2.159704	1.233238
H	3.889273	-1.160689	-0.266407
H	3.009981	1.003882	-0.265631
H	2.302446	1.721962	1.234165
H	2.279591	0.386251	3.290393
H	2.921425	-1.291932	3.244461
N	3.149031	-0.201043	1.456040
C	4.452396	0.439704	1.807538
H	4.978427	0.662905	0.873138
H	4.223067	1.353500	2.367770
Cl	5.472033	-0.607794	2.802542
F	-0.645736	-1.664738	0.411133
C	-2.696892	-2.343599	1.619822
C	-2.849809	-0.928031	-0.457385
H	-2.550335	-3.095930	-0.388709
O	-2.760572	-3.452834	2.114183
C	-2.549516	-2.162794	0.170458
C	-2.691297	-1.113201	2.467354
C	-2.535761	0.298073	0.392912
C	-2.605276	0.152729	1.869203
C	-2.691120	-1.225755	3.856503
H	-2.743793	-2.219581	4.303116

C	-2.507260	1.299317	2.661463
H	-2.417848	2.274235	2.181018
C	-2.513066	1.181842	4.049921
H	-2.430276	2.076497	4.669004
C	-2.606640	-0.078311	4.645968
H	-2.603506	-0.166767	5.733551
O	-2.134645	1.314963	-0.124041
N	-3.168683	-0.788264	-1.727216
C	-3.089336	-1.889856	-2.697196
H	-4.035263	-1.899704	-3.258024
H	-2.986141	-2.849523	-2.188090
C	-3.480700	0.483270	-2.403988
H	-3.695002	1.264021	-1.674347
H	-4.395173	0.289122	-2.984532
C	-1.910170	-1.614298	-3.632977
H	-0.978900	-1.609786	-3.045122
H	-1.842495	-2.439516	-4.356907
C	-2.334738	0.852938	-3.342677
H	-1.432104	1.032666	-2.740185
H	-2.590953	1.789966	-3.858179
C	-2.077786	-0.272355	-4.345256
H	-2.929929	-0.337346	-5.042605
H	-1.182434	-0.049369	-4.942668
F	0.833869	0.193788	-3.117906
F	2.828651	-0.365963	-2.152773
F	1.460637	-1.999385	-2.980783
F	2.551537	-0.587611	-4.413311
F	1.935861	2.179749	4.485961
F	2.153717	3.472355	2.615587
F	0.513250	1.888120	2.721932
F	0.357502	3.768330	4.010373
B	1.239794	2.841679	3.467960
B	1.919209	-0.690688	-3.180850

TS$_{ISET}$

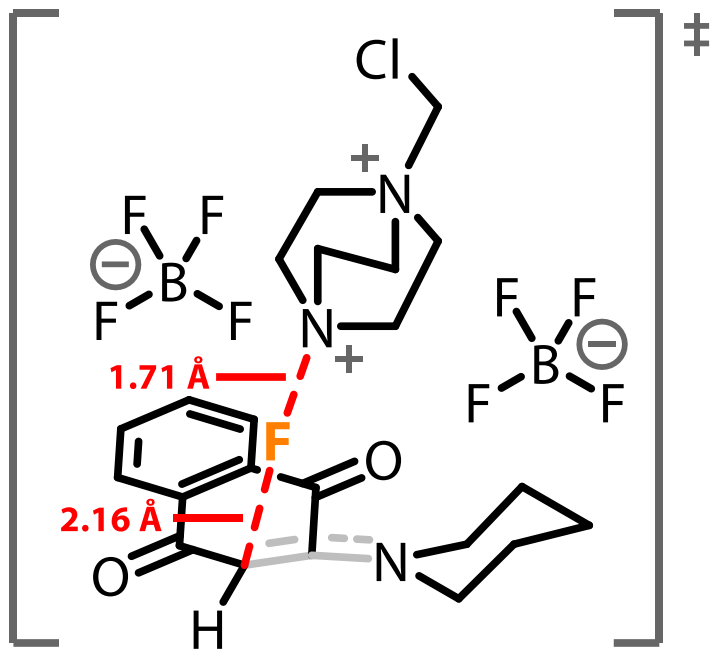

N	0.944323	-1.088546	0.829598
C	1.930390	-2.011862	0.282142
C	0.950802	-1.050793	2.284706
C	1.014402	0.241535	0.241881
C	2.390501	0.823036	0.626134
C	2.340487	-0.518810	2.704878
C	3.325413	-1.444393	0.646644
H	1.764190	-2.993771	0.737838
H	1.793472	-2.056798	-0.803576
H	0.907379	0.147024	-0.844225
H	0.208428	0.847075	0.670902
H	0.155430	-0.371231	2.614147
H	0.778164	-2.066658	2.657101
H	3.900161	-2.151743	1.252555
H	3.884593	-1.158886	-0.251320
H	3.002990	1.004886	-0.262899
H	2.291408	1.730221	1.231398
H	2.260095	0.402077	3.292865
H	2.904439	-1.276448	3.257785
N	3.137409	-0.192234	1.464996
C	4.439734	0.451421	1.818871
H	4.967982	0.671505	0.884960
H	4.207012	1.367041	2.374800
Cl	5.455756	-0.591850	2.820921
F	-0.603773	-1.670205	0.405348
C	-2.717719	-2.356170	1.619007
C	-2.896095	-0.941368	-0.460296
H	-2.723751	-3.118696	-0.388347
O	-2.755118	-3.465603	2.118309
C	-2.684941	-2.183113	0.165181
C	-2.669652	-1.126888	2.465511
C	-2.543552	0.277312	0.384991
C	-2.590752	0.136864	1.863249
C	-2.634038	-1.238273	3.854127
H	-2.682384	-2.231284	4.303029

C	-2.465217	1.283515	2.651094
H	-2.379878	2.257161	2.167454
C	-2.436744	1.168162	4.039580
H	-2.332103	2.063299	4.654655
C	-2.521715	-0.090453	4.639746
H	-2.490917	-0.177596	5.727025
O	-2.133441	1.288166	-0.137029
N	-3.186494	-0.789774	-1.743151
C	-3.097144	-1.885892	-2.716653
H	-4.037040	-1.897231	-3.288106
H	-2.994536	-2.848276	-2.212984
C	-3.481916	0.486699	-2.415348
H	-3.700911	1.264052	-1.683405
H	-4.391369	0.302054	-3.007582
C	-1.908432	-1.607298	-3.640109
H	-0.983000	-1.606922	-3.042969
H	-1.834645	-2.429592	-4.366888
C	-2.326260	0.858423	-3.341137
H	-1.428276	1.029801	-2.729411
H	-2.573524	1.799820	-3.853162
C	-2.065587	-0.262058	-4.347908
H	-2.912577	-0.320752	-5.052174
H	-1.164701	-0.039514	-4.937119
F	0.837035	0.182831	-3.116554
F	2.825196	-0.370352	-2.134184
F	1.465992	-2.008532	-2.966090
F	2.565727	-0.600404	-4.396092
F	1.916250	2.187623	4.485044
F	2.139450	3.485802	2.618986
F	0.506145	1.894536	2.711152
F	0.333301	3.770082	4.004246
B	1.223580	2.849608	3.464654
B	1.923945	-0.700067	-3.168547

PROD

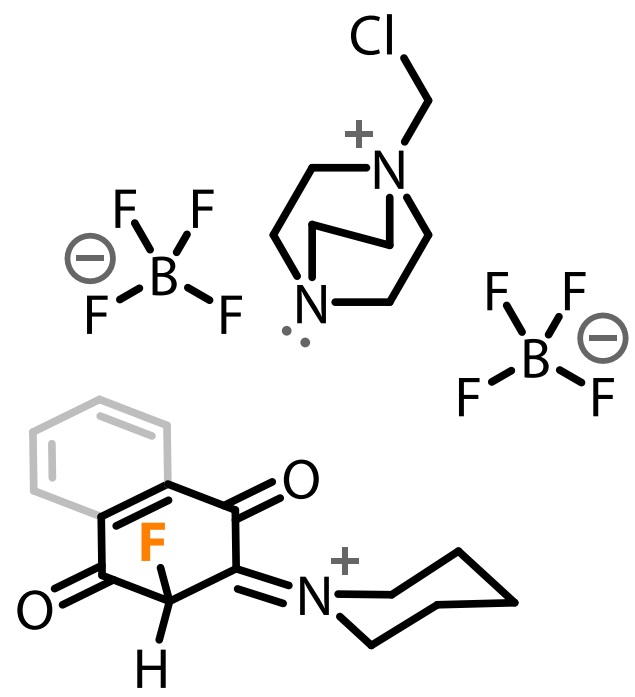

N	0.039598	-0.793750	0.441848
C	0.802121	-1.902107	-0.129568
C	0.488298	-0.546782	1.812582
C	0.268302	0.404395	-0.362696
C	1.727672	0.875722	-0.222038
C	2.017876	-0.340210	1.870987
C	2.295958	-1.528411	-0.249629
H	0.665405	-2.782493	0.512497
H	0.407604	-2.132091	-1.126745
H	0.057507	0.156733	-1.409440
H	-0.396972	1.211655	-0.025882
H	-0.017344	0.349875	2.189956
H	0.213554	-1.403966	2.441156
H	2.950214	-2.265943	0.227477
H	2.605012	-1.370354	-1.287806
H	2.200341	1.072664	-1.190457
H	1.814868	1.753380	0.429793
H	2.291008	0.585293	2.392664
H	2.538328	-1.192227	2.321850
N	2.519839	-0.218626	0.459318
C	3.944205	0.204884	0.393051
H	4.229334	0.232547	-0.664815
H	4.018668	1.193904	0.858280
Cl	5.023777	-0.908260	1.251660
F	-1.889516	-2.974967	-0.678840
C	-2.231861	-2.036915	1.424462
C	-2.738752	-0.754195	-0.737821
H	-3.738691	-2.488350	-0.021080
O	-1.700274	-3.012369	1.887604
C	-2.703269	-2.092397	-0.034539
C	-2.496173	-0.811753	2.214802
C	-3.093963	0.465273	0.127871
C	-2.825438	0.394820	1.580494
C	-2.309438	-0.844589	3.597351
H	-2.053367	-1.791791	4.074033
C	-2.944732	1.570598	2.329565

H	-3.188507	2.503908	1.821033
C	-2.731966	1.536554	3.704061
H	-2.803583	2.456009	4.286755
C	-2.425928	0.328424	4.338776
H	-2.267068	0.305978	5.417869
O	-3.669421	1.395893	-0.376891
N	-2.680433	-0.659143	-2.019901
C	-2.825171	-1.821132	-2.938179
H	-3.709084	-1.556756	-3.538766
H	-3.054279	-2.722142	-2.372045
C	-2.553192	0.613459	-2.787499
H	-2.368449	1.436935	-2.099136
H	-3.528442	0.762829	-3.273747
C	-1.609388	-1.963212	-3.839066
H	-0.710866	-2.112396	-3.220847
H	-1.751074	-2.861751	-4.455158
C	-1.419971	0.525855	-3.810688
H	-0.456112	0.560986	-3.287881
H	-1.485153	1.445042	-4.410810
C	-1.464188	-0.712120	-4.697671
H	-2.317794	-0.651659	-5.392371
H	-0.548111	-0.762239	-5.303453
F	1.733341	1.331779	-3.406897
F	3.468220	-0.047841	-2.863810
F	1.362278	-0.903865	-3.104610
F	2.512410	-0.179423	-4.942480
F	0.730695	1.867624	3.783460
F	2.232728	2.876563	2.387996
F	0.051894	2.654743	1.744840
F	0.603746	4.120365	3.410899
B	0.901019	2.886903	2.839232
B	2.268450	0.052978	-3.594105

Cytotoxicity and anti-*Trypanosoma cruzi* activity assays

Animals

All animal procedures were performed in accordance with the Guidelines for Care and Use of the Laboratory Animals of Oswaldo Cruz Institute (CEUA/IOC) and approved by the Animal Ethics Committee under the license L002/2024 from the Ethics Committee for Animal Use of the Oswaldo Cruz Institute. All experiments dealing with animals were also performed in accordance with the Brazilian Law 11.794/2008. The Albino Swiss mice were housed at a maximum of 6 individuals per cage, kept in a specific-pathogen-free (SPF) room at 20 to 22 °C under a 12/12 h light/dark cycle, 50 to 60% humidity and provided sterilized water and chow *ad libitum*.

Cytotoxicity to mammalian cells

The cytotoxicity assays were performed using primary cultures of peritoneal macrophages obtained from Albino Swiss mice. For the experiments, 2.5 × 104 cells in 200 µL of RPMI-1640 medium (pH 7.2 plus 10% foetal bovine serum and 2 mM glutamine) were added to each well of a 96-well microtiter plate and incubated for 24 h at 37 °C. The treatment of the cultures was performed in fresh supplemented medium (200 µL/well) for 24 h at 37 °C. After this period, 110 µL of the medium was discarded and 10 µL of PrestoBlue (Invitrogen) was added to complete the final volume of 100 µL. Thus, the plate was incubated for 2 h and the measurement was performed at 560 and 590 nm, as recommended by the manufacturer. The results were expressed as the difference in the percentage of reduction between treated and untreated cells being the LC50/24 h value, corresponding to the concentration that leads to damage of 50% of the mammalian cells.[17]

Trypanocidal assays

5% of blood at 4 °C: For all the experiments stock solutions of the compounds were prepared in dimethyl sulfoxide, with the final concentration of the solvent never exceeding 0.5%, concentration known to exert no toxicity to the parasite or host cells.[18] Bloodstream trypomastigotes of Y strain[19] were obtained from infected Swiss Webster mice at the peak of parasitaemia by differential centrifugation process. The parasites (5 × 10^6 parasites per mL) plus 5% of blood were incubated at 4 °C for 24 h in 5% CO_2 atmosphere in absence or presence of the compounds. Parasites counts were performed by light microscopy, and the activity of the compounds was expressed as IC_{50}/24 h, corresponding to the concentration that led to 50% lysis of the parasites. At least three independent experiments were performed, and the mean and standard deviation were calculated. The standard drug Bz was used as control.

0% of blood at 37 °C: The experiments were performed with the Y strain of *T. cruzi*.[20] Stock solutions of the compounds were prepared in dimethylsulfoxide (DMSO), with the final

concentration of the latter in the experiments never exceeding 0.1%. Preliminary experiments showed that at concentrations of up to 0.5%, DMSO has no deleterious effect on parasites. Bloodstream trypomastigotes were obtained from infected Albino Swiss mice at the peak of parasitemia by differential centrifugation. The parasites were resuspended to a concentration of 10×10^6 cells/mL in DMES medium. This suspension (100 μL) was added to the same volume of each of the compounds, which had been previously prepared at twice the desired final concentrations. The incubation was performed in 96-well microplates (Nunc Inc., Rochester, USA) at 37 ºC for 24 h. Bz (Lafepe, Brazil), the standard drug for treatment of chagasic patients, was used as control. Cell counts were performed in a Neubauer chamber, and the activity of the compounds corresponding to the concentration that led to 50% lysis of the parasites was expressed as the IC_{50}/24 h.

Cyclic voltammetry

Data was acquired on an Autolab/M101 potentiostat. All measurements were recorded using a three-electrode cell setup with a homemade Ag/$AgNO_3$ (0.01 M $AgNO_3$ in MeCN/0.1 M $TBAPF_6$) reference electrode. The measurements were conducted in pure DMSO using $TBAPF_6$ (0.1 M) as the electrolyte with a scan rate at 0.1 V/s. A platinum wire was used as the counter electrode. Prior to any measurement, the glassy carbon working electrode (3 mm diameter, 0.0707 cm^2 geometric surface area) was polished with a polishing slurry (0.3 mm aluminum oxide) on a microcloth polishing disc. Residual traces of slurry were removed by sonicating the as-polished electrode three times in water (1 min each) and one time in acetone (1 min). The solution was degassed before each measurement.

Cyclic voltammetry plots of measured compounds

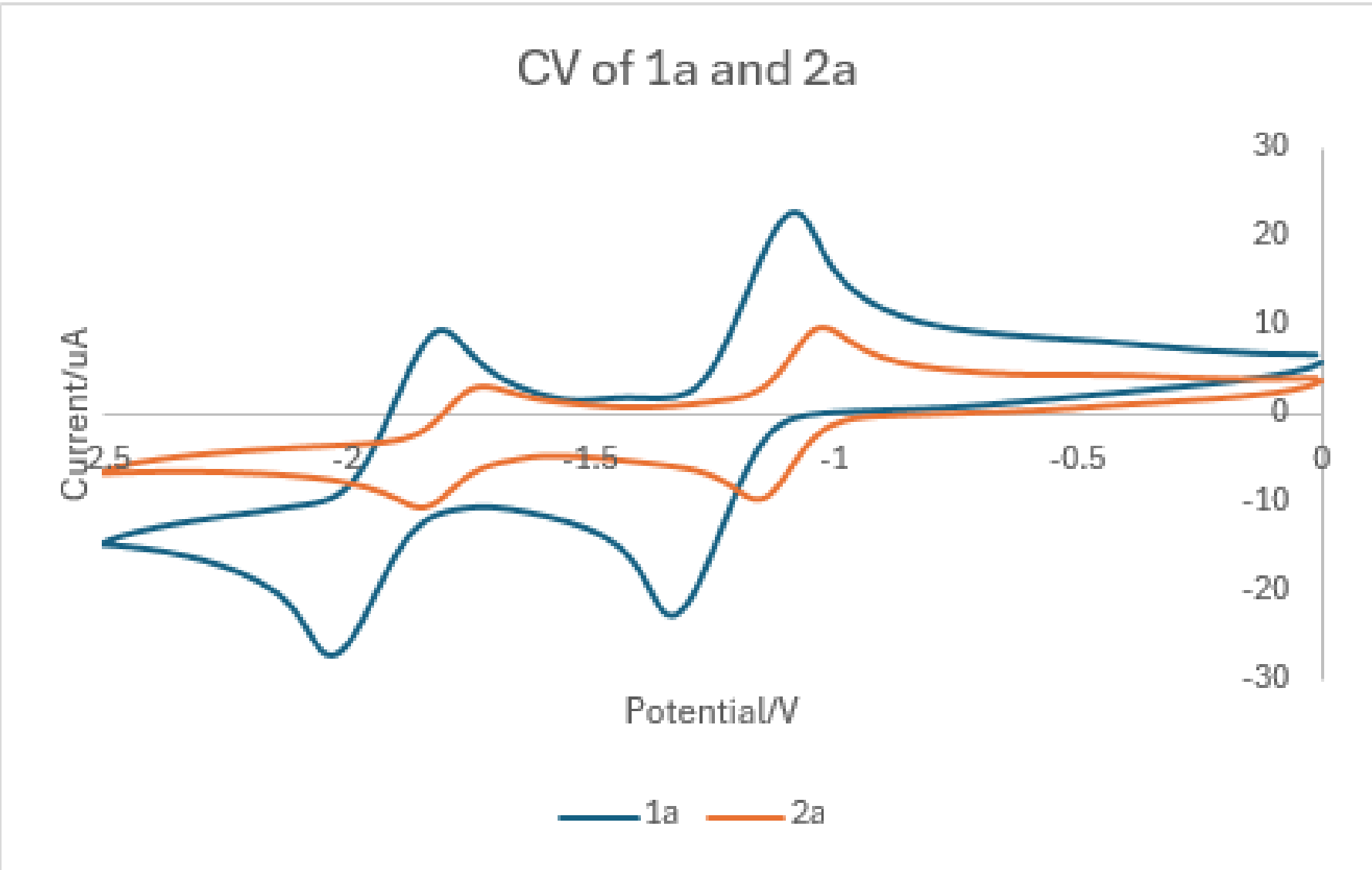

Figure S1. Cyclic voltammetry of **1a** and **2a** at 100 mV/s at a glassy carbon working electrode in DMSO.

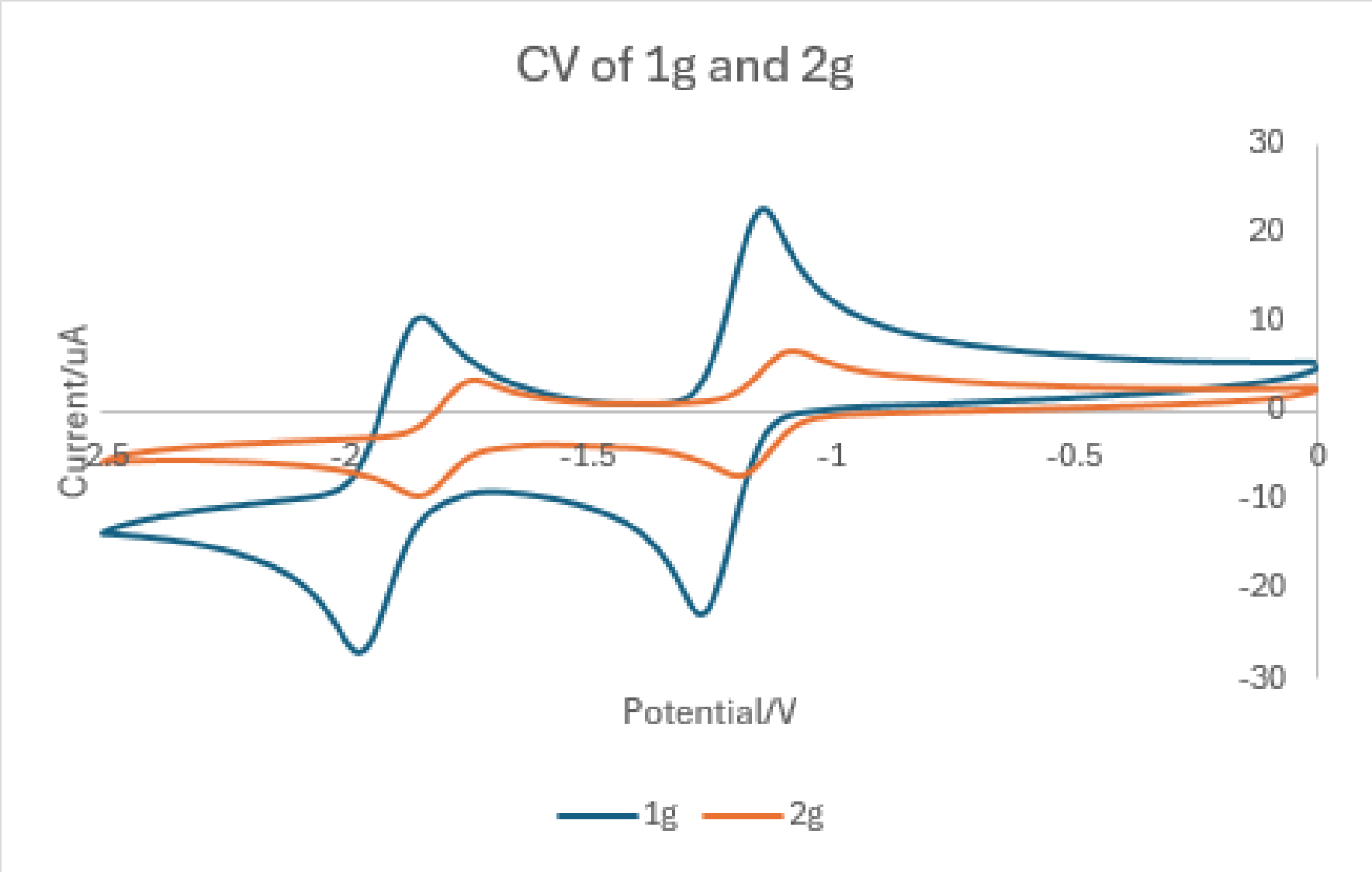

Figure S2. Cyclic voltammetry of **1g** and **2g** at 100 mV/s at a glassy carbon working electrode in DMSO.

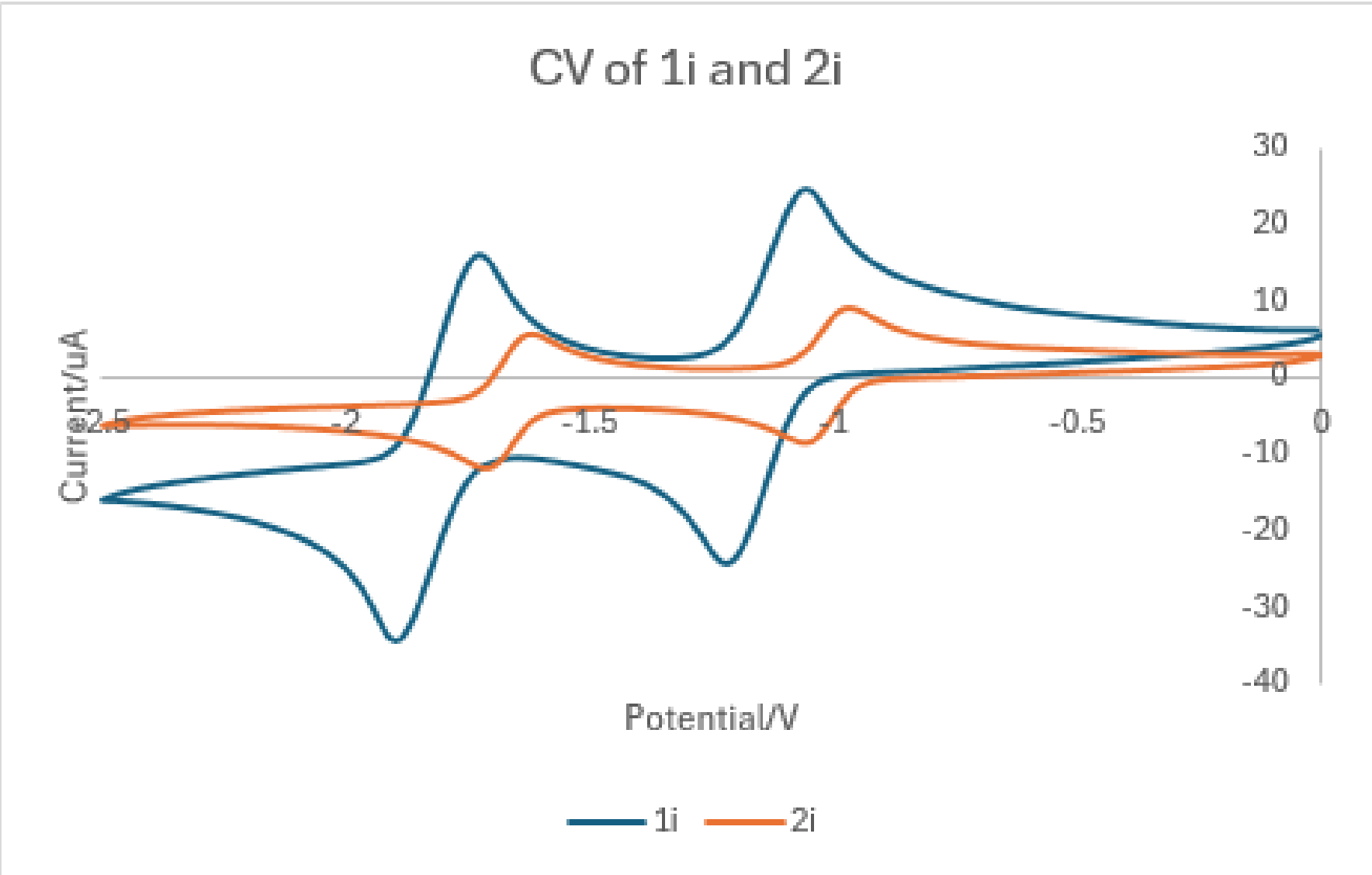

Figure S3. Cyclic voltammetry of **1i** and **2i** at 100 mV/s at a glassy carbon working electrode in DMSO.

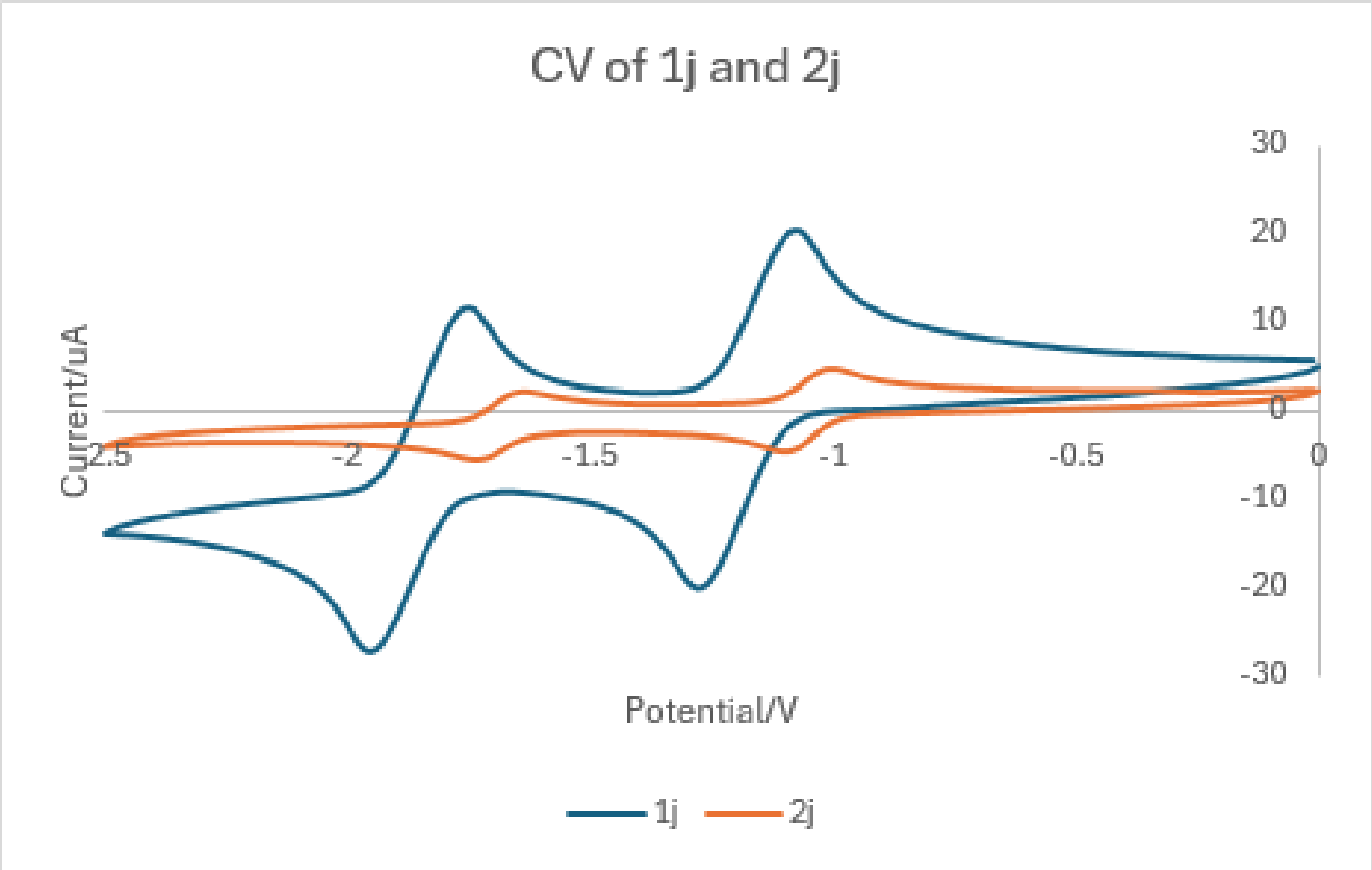

Figure S4. Cyclic voltammetry of **1j** and **2j** at 100 mV/s at a glassy carbon working electrode in DMSO.

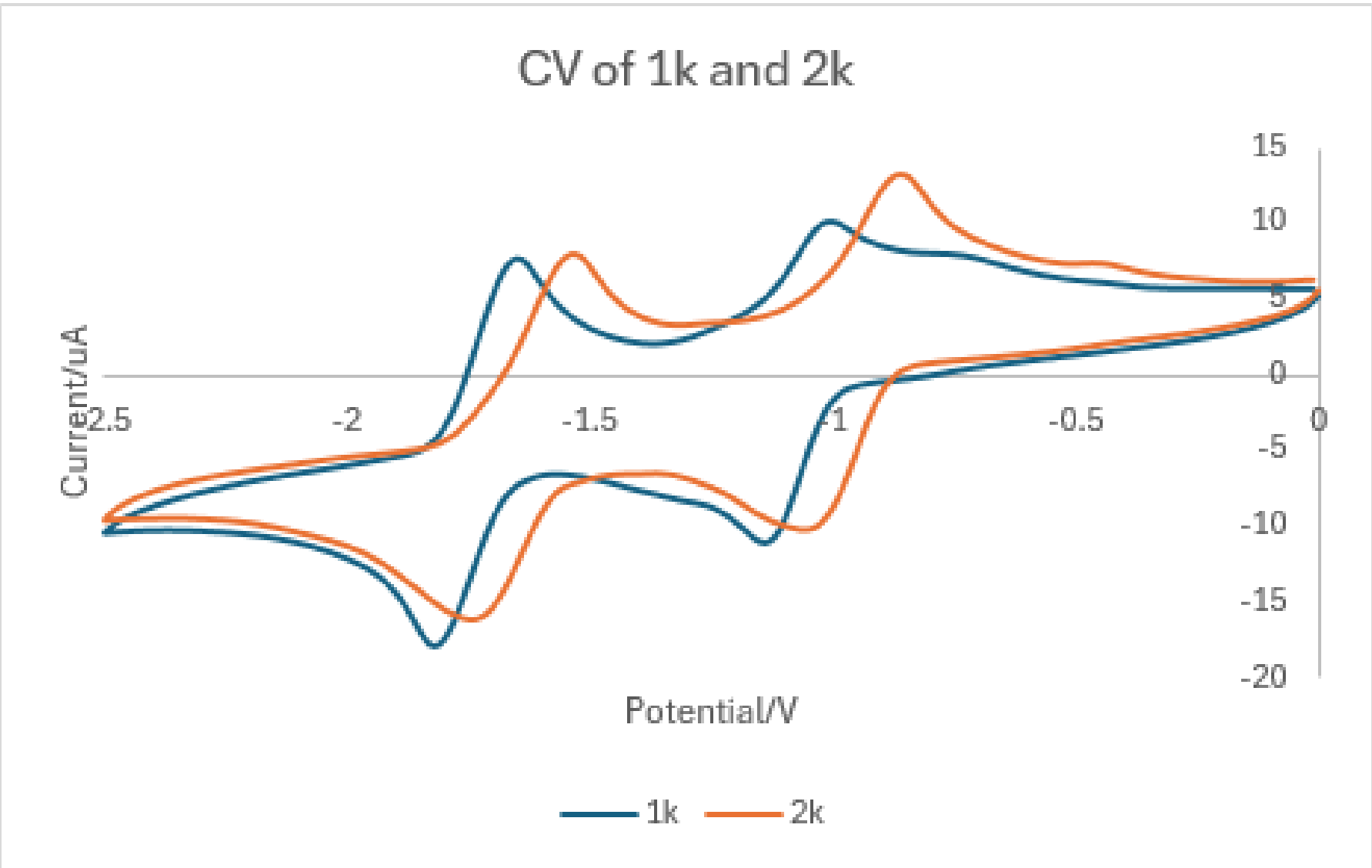

Figure S5. Cyclic voltammetry of **1k** and **2k** at 100 mV/s at a glassy carbon working electrode in DMSO.

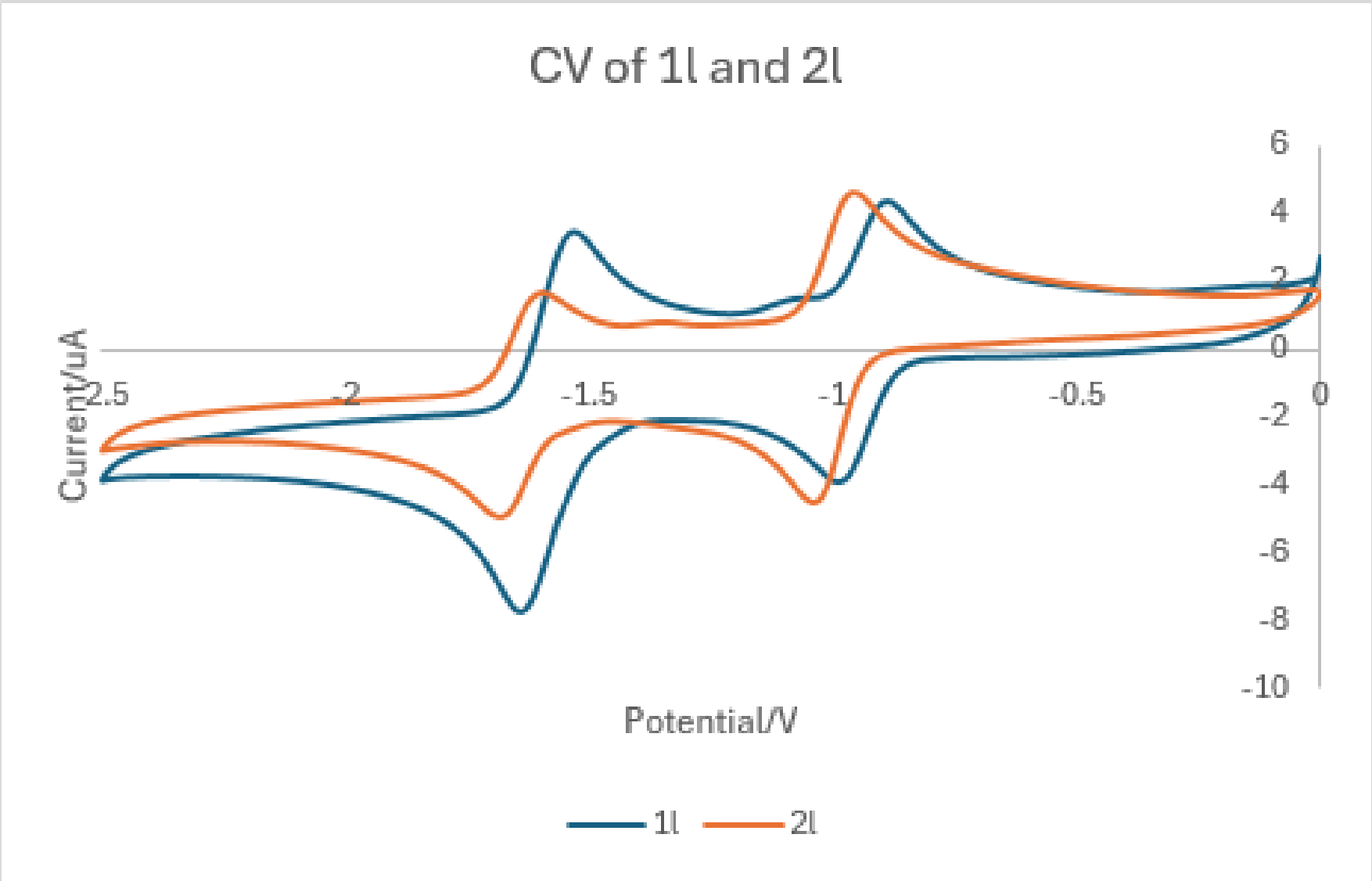

Figure S6. Cyclic voltammetry of **1l** and **2l** at 100 mV/s at a glassy carbon working electrode in DMSO.

cLogP values

Table S1. Calculated cLogP values of fluorinated and non-fluorinated amino-quinones

Entry	Compound	Structure	cLogP
**1**	**1a**	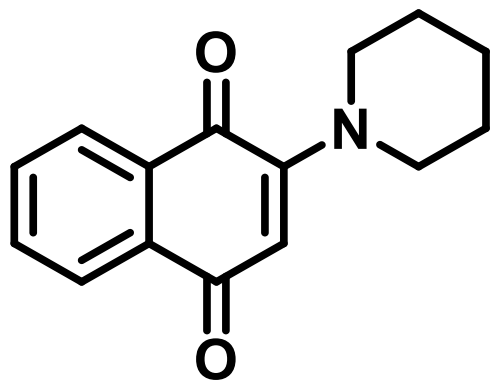	**2.96**
**2**	**2a**	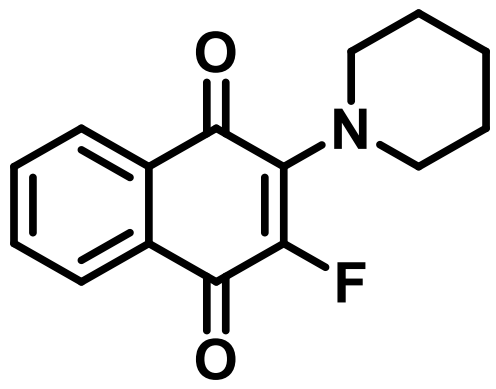	**3.13**
**3**	**1b**	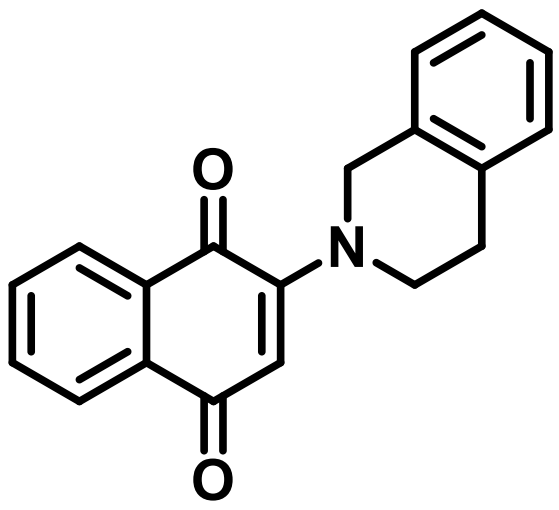	**3.79**
**4**	**2b**	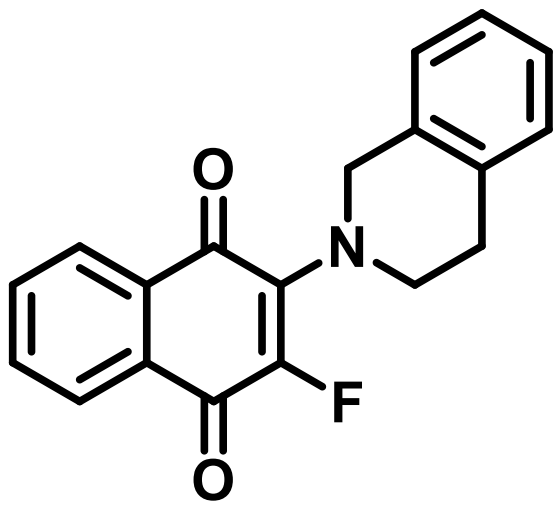	**3.96**
**5**	**1c**	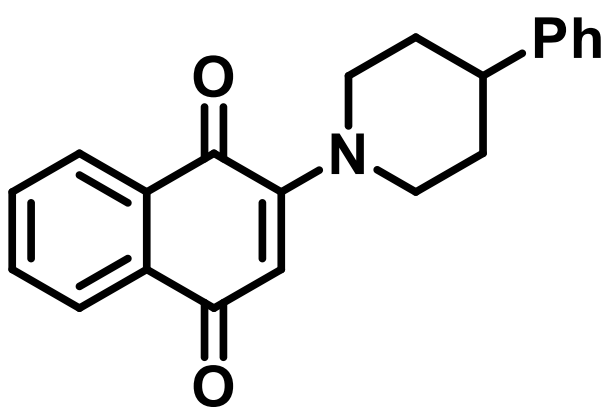	**4.37**
**6**	**2c**	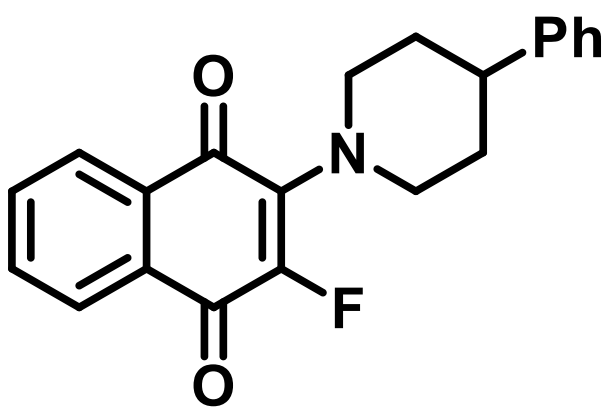	**4.54**
**7**	**1d**	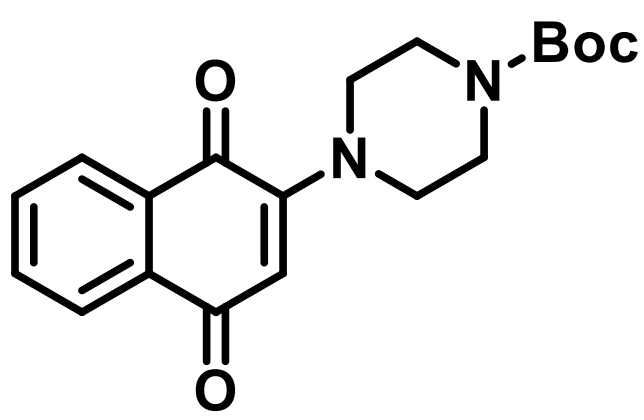	**3.63**

**8**	**2d**	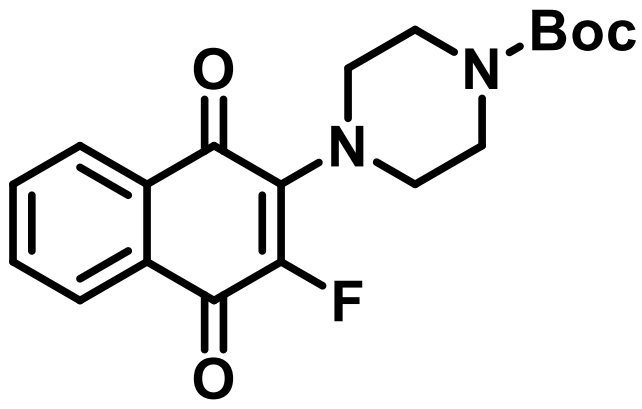	**3.80**
**9**	**1e**	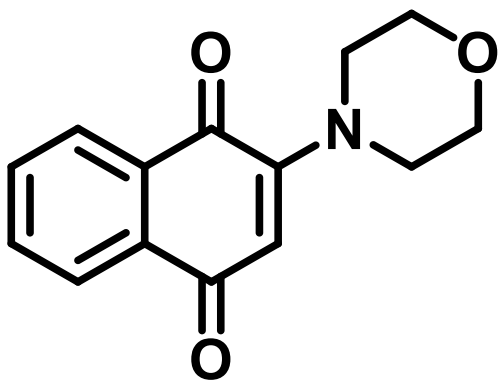	**1.66**
**10**	**2e**	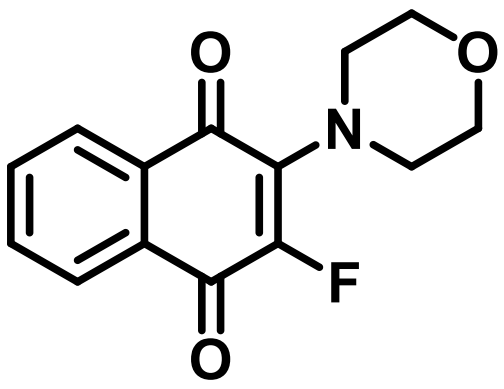	**1.83**
**11**	**1f**	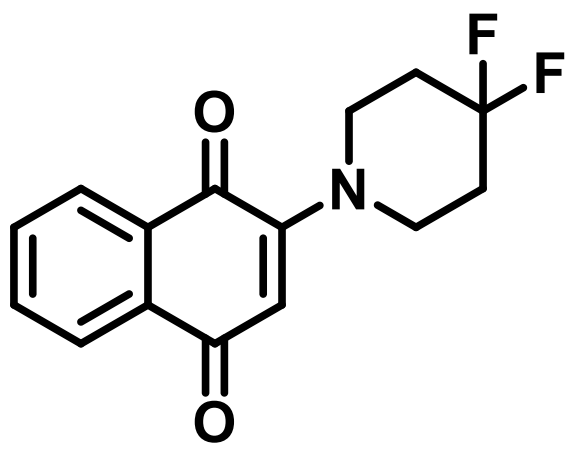	**2.35**
**12**	**2f**	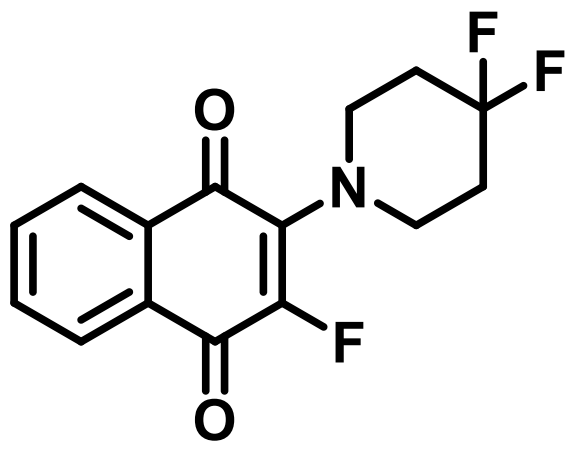	**2.52**
**13**	**1g**	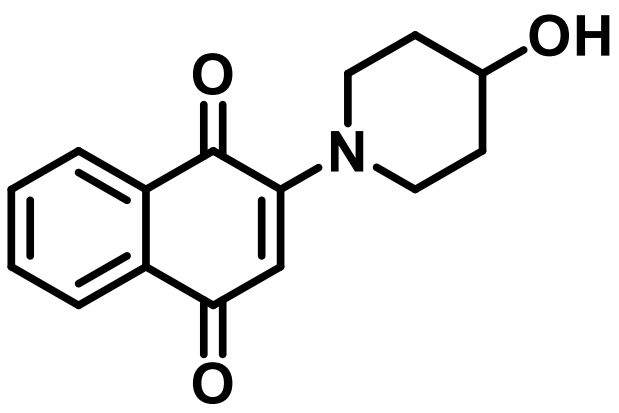	**0.87**
**14**	**2g**	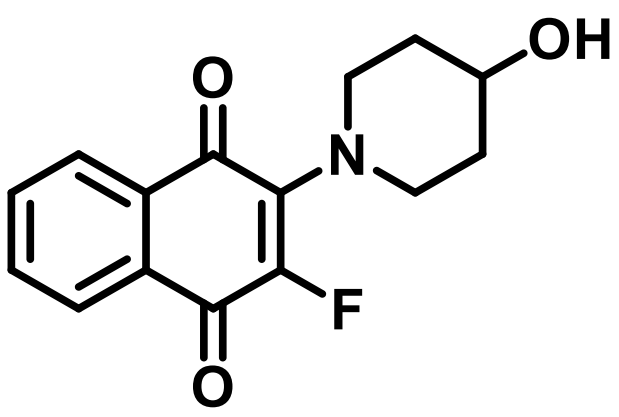	**1.05**
**15**	**1h**	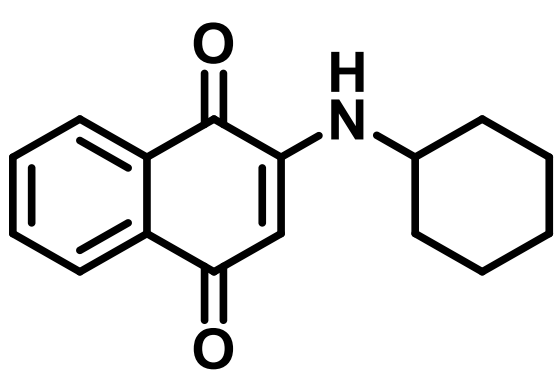	**4.05**

16	2h	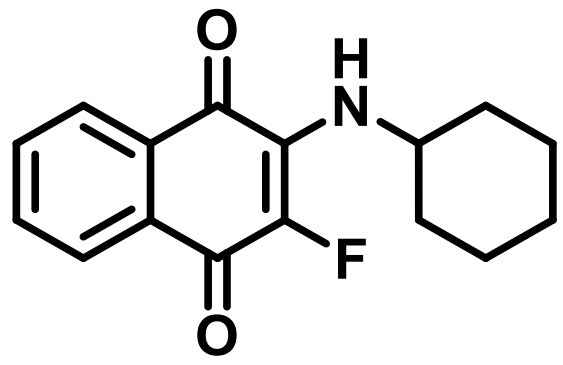	4.22
17	1i	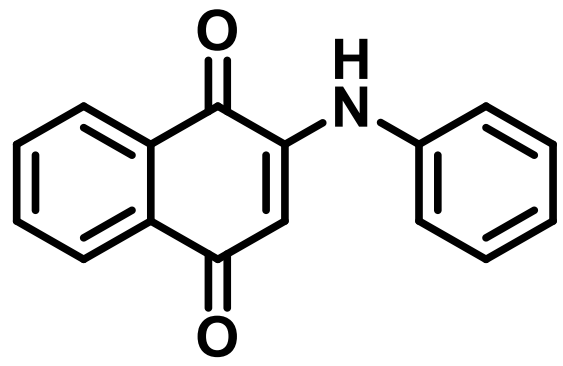	3.98
18	2i	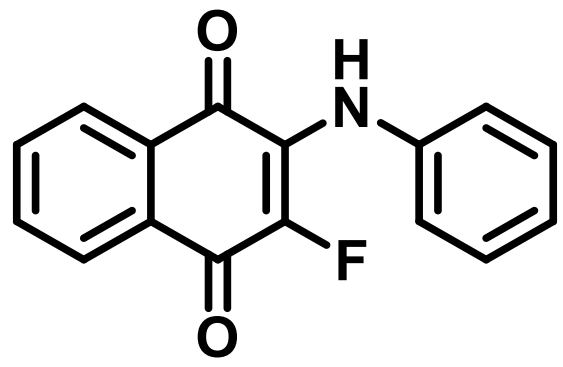	4.15
19	1j	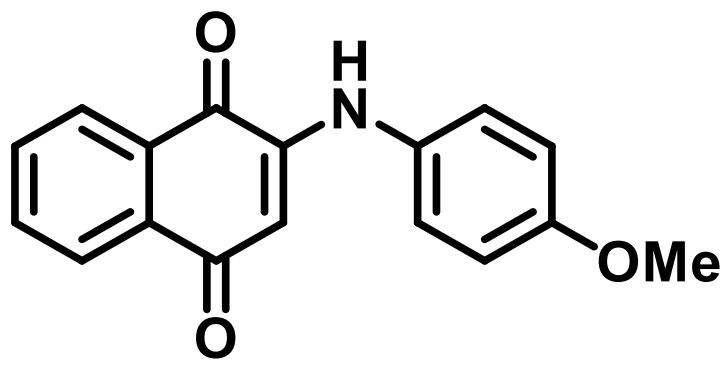	4.08
20	2j	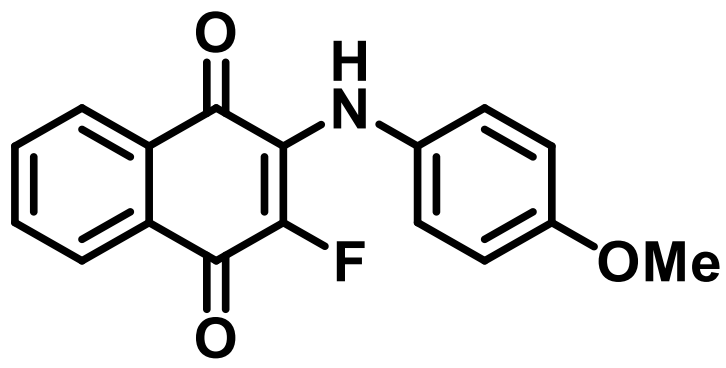	4.25
21	1k	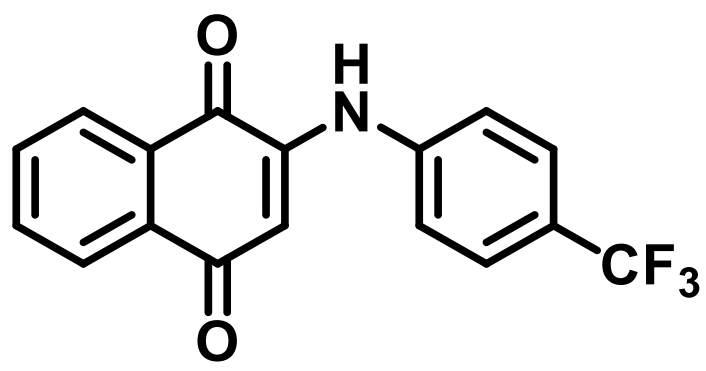	5.39
22	2k	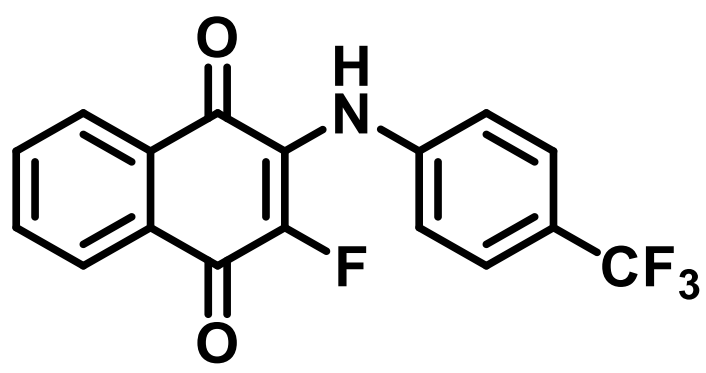	5.56
23	1l	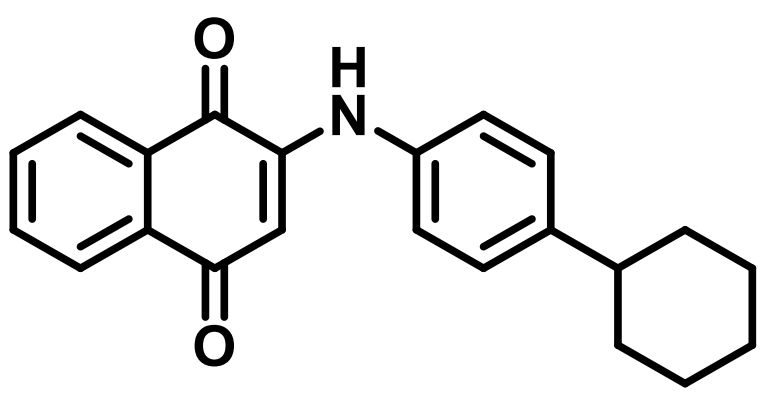	6.60
24	2l	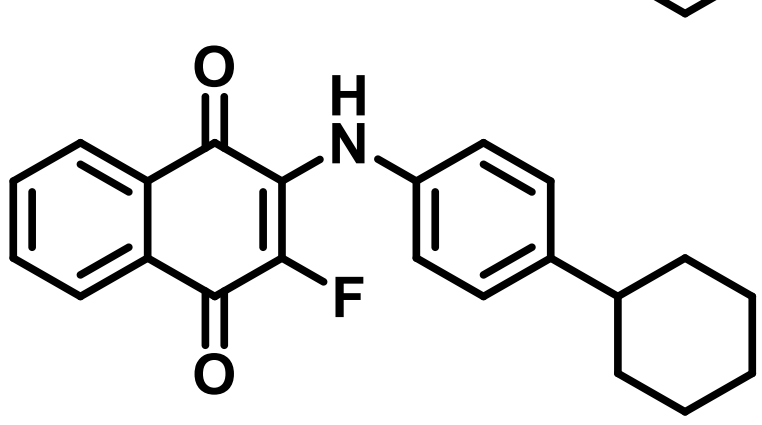	6.77

**25**	**1m**	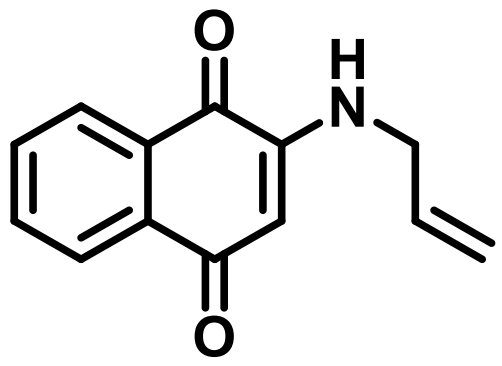	**2.79**
**26**	**2m**	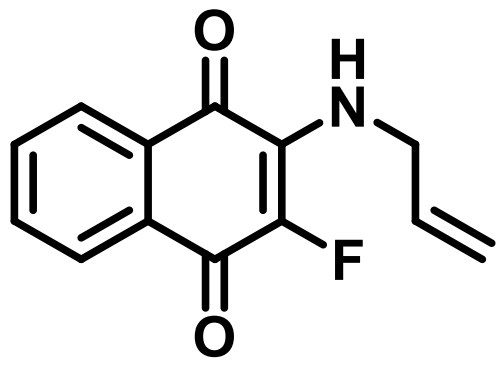	**2.96**
**27**	**1n**	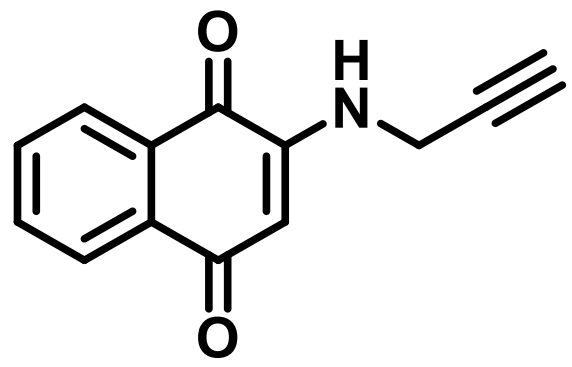	**2.44**
**28**	**2n**	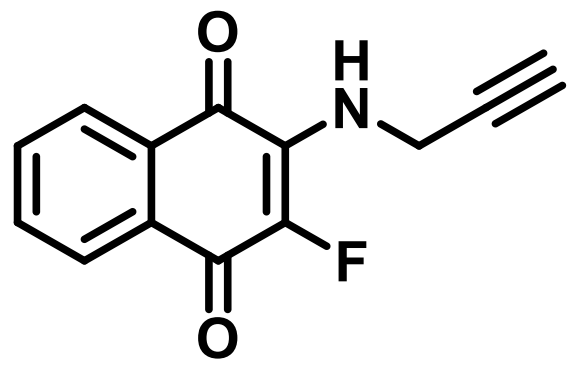	**2.61**
**29**	**1p**	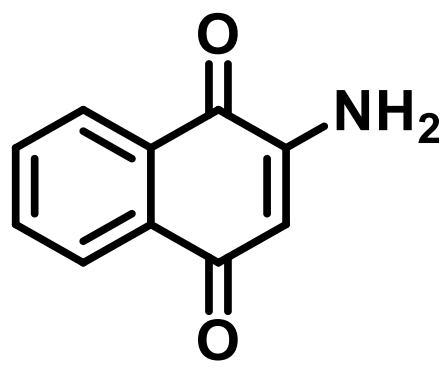	**1.98**
**30**	**2p**	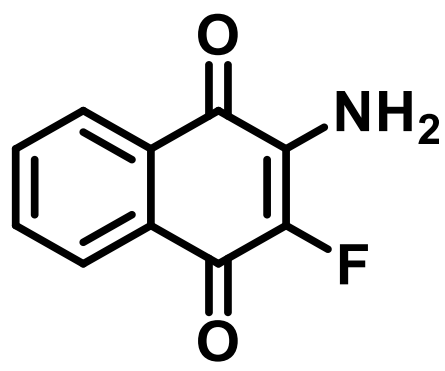	**2.15**

References

[1] M. Bhuyan and G. Baishya. *Org. Biomol. Chem.* 2022, **20**, 9172-9183.

[2] C. S. Lisboa, V. G. Santos, B. G. Vaz, N. C. Lucas, M. N. Eberlin and S. J. Garden, *J. Org. Chem.* 2011, **76**, 5264–5273.

[3] B. Liu and S. Ji, *Synth. Commun.* 2008, **38**, 1201–1211.

[4] X. Wu, X. Zhao, L. Zhang, G. Li and Y. He, *Tetrahedron Lett.* 2022, **112**, 154208.

[5] H. Yang, X. Wu, M. Li, Y. Wang, M. Guo, L. Chen, R. Ma and M. Sun, *Bioorg. Med. Chem. Lett.* 2025, **129**, 130360.

[6] R. K. Jha, M. Batabyal and S. Kumar, *J. Org. Chem.* 2023, **88**, 7401–7424.

[7] T. D. Suja, K. V. L. Divya, L. V. Naik, A. R. Kumar and A. Kamal, *Bioorg. Med. Chem. Lett.* 2016, **26**, 2072–2076.

[8] F. Neese, *Wiley Interdiscip. Rev. Comput. Mol. Sci.*, 2025, **15**, e70019.

[9] Y. Zhao and D. G. Truhlar, *Theor. Chem. Acc.*, 2008, **120**, 215–241.

[10] F. Weigend and R. Ahlrichs, *Phys. Chem. Chem. Phys.*, 2005, **7**, 3297–3305.

[11] F. Weigend, *Phys. Chem. Chem. Phys.*, 2006, **8**, 1057–1065.

[12] S. Grimme, J. Antony, S. Ehrlich and H. Krieg, *J. Chem. Phys.*, 2010, **132**, 154104.

[13] A. V. Marenich, C. J. Cramer and D. G. Truhlar, *J. Phys. Chem. B*, 2009, **113**, 6378–6396.

[14] F. Neese, F. Wennmohs, A. Hansen and U. Becker, *Chem. Phys.*, 2009, **356**, 98–109.

[15] B. Helmich-Paris, B. de Souza, F. Neese and R. Izsák, *J. Chem. Phys.*, 2021, **155**, 104109.

[16] S. Grimme, *Chem. Eur. J.*, 2012, **18**, 9955–9964.

[17] S. L. de Castro, M. C. Pinto and A. V. Pinto, *Microbios*. 1994, **78**, 83–90.

[18] J. M. Wood, N. S. Satam, R. G. Almeida, V. S. Cristani, D. P. de Lima, L. Dantas-Pereira, K. Salomao, R. F. S. Menna-Barreto, I. N. N. Namboothiri, J. F. Bower and E. N. da Silva Junior, *Bioorg. Med. Chem.*, 2020, **28**, 115565.

[19] L. F. Umekita, H. A. Takehara and I. Mota, *Folia Clin. Biol.*, 1953, **20**, 191–207.

[20] (a) P. A. S. Junior, I. Molina, S. M. F. Murta, A. Sanchez-Montalva, F. Salvador, R. Correa-Oliveria and C. M. Carneiro, *Am. J. Trop. Med. Hyg.*, 2017, **97**, 1289–1303. (b) J. Bermudez, C. Davies, A. Simonazzi, J. P. Real and S. Palma, *Acta Trop.*, 2016, **156**, 1–16. (c) Y. Jackson, B. Wyssa and F. Chappuis, *J. Antimicrob. Chemother.*, 2020, **75**, 690–696.

NMR spectra of novel compounds

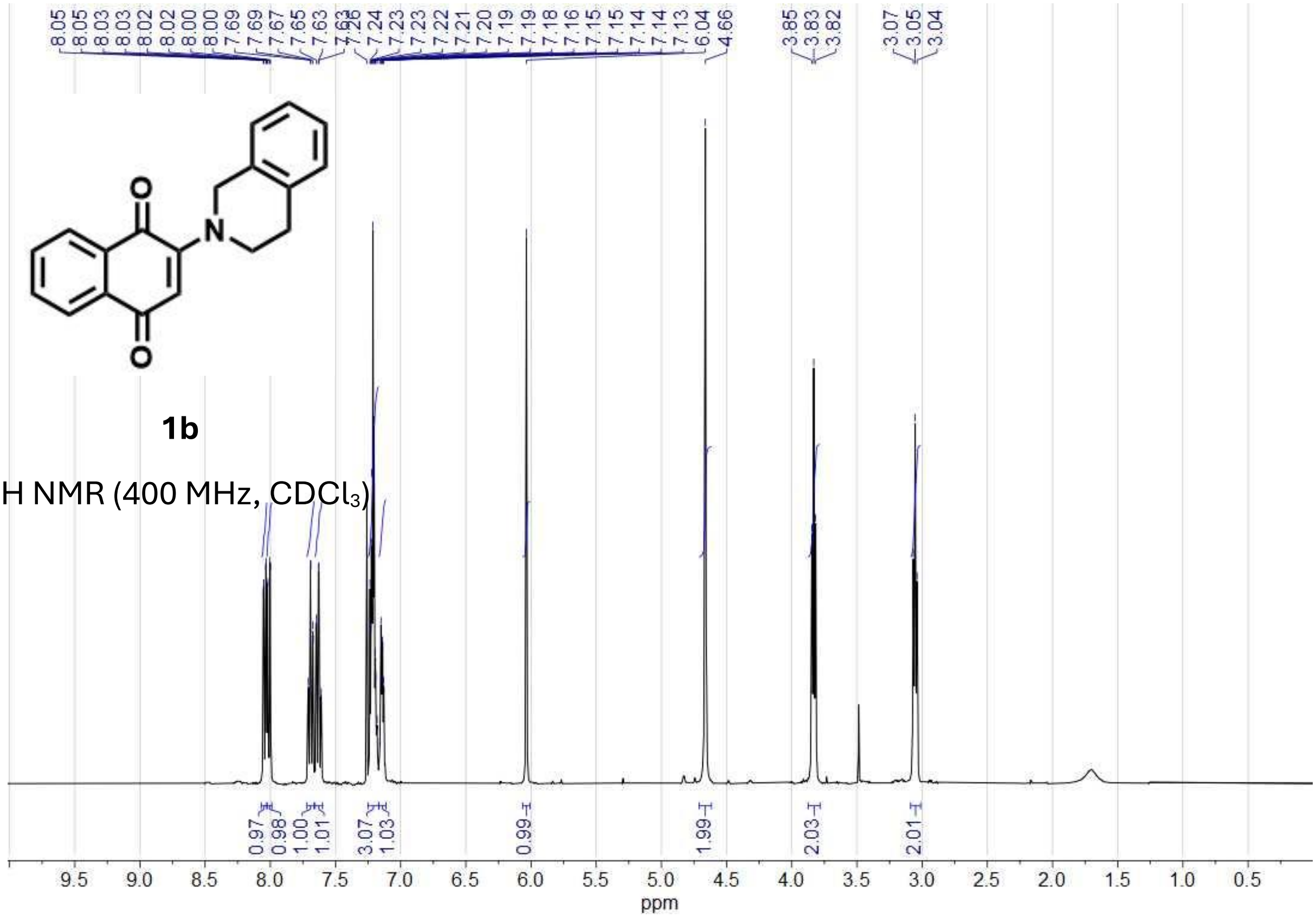

1b

^{1}H NMR (400 MHz, $CDCl_3$)

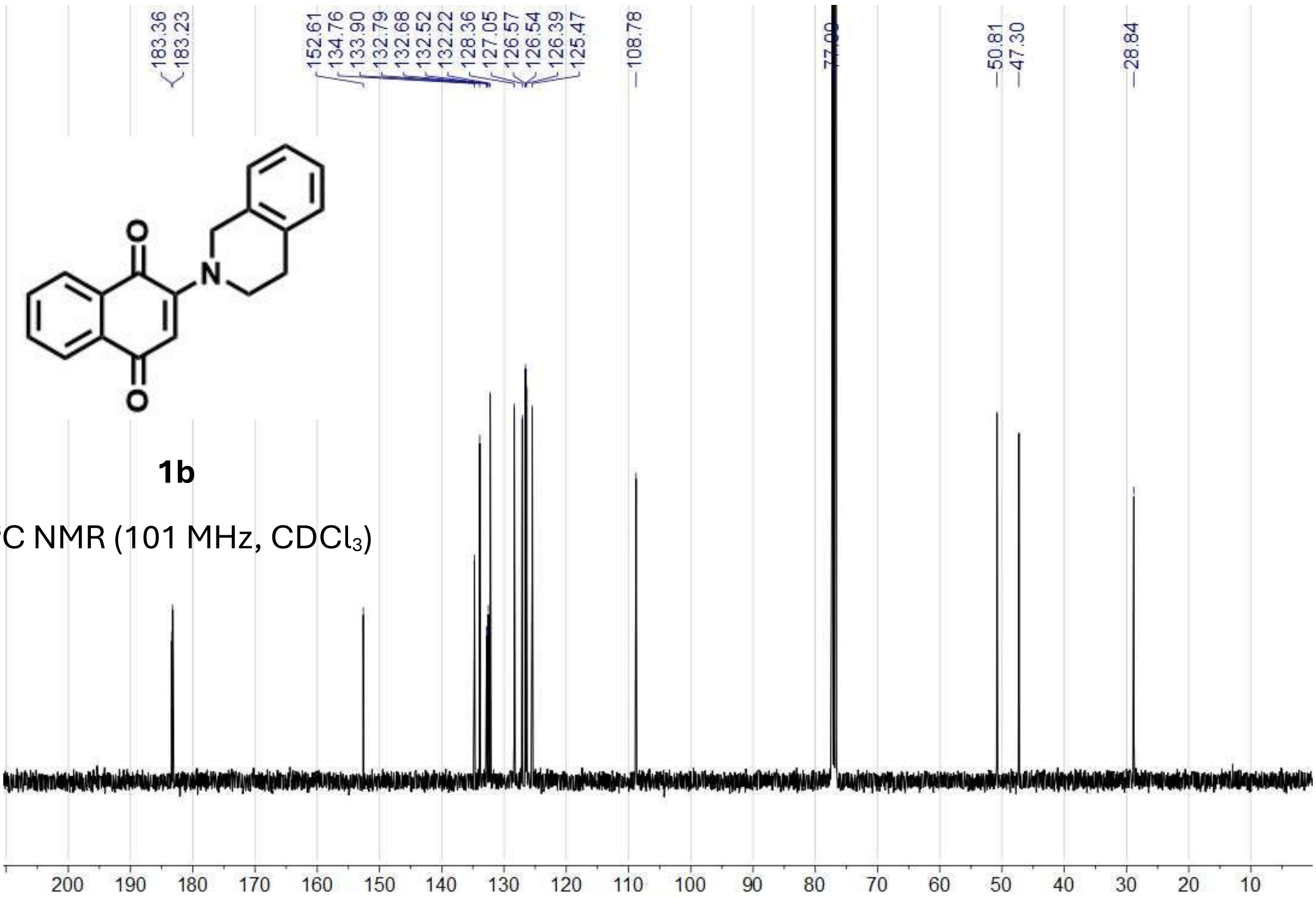

1b

^{13}C NMR (101 MHz, $CDCl_3$)

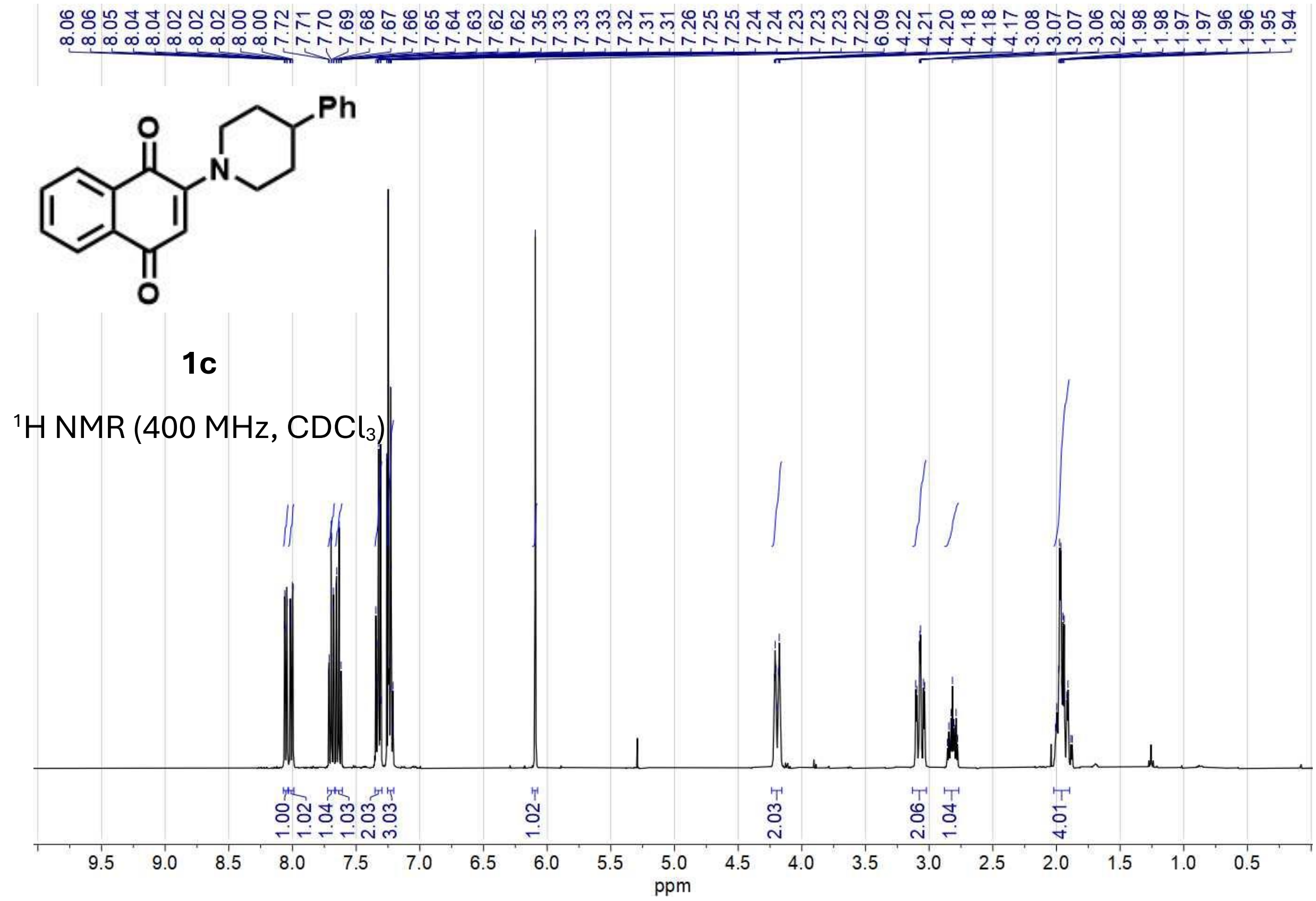
8.06 8.05 8.04 8.04 8.02 8.02 8.02 8.00 8.00 7.72 7.71 7.70 7.69 7.68 7.67 7.66 7.65 7.64 7.63 7.62 7.62 7.35 7.33 7.33 7.33 7.32 7.31 7.31 7.26 7.25 7.25 7.24 7.24 7.23 7.23 7.23 7.22 6.09 4.22 4.21 4.20 4.18 4.18 4.17 3.08 3.07 3.07 3.06 2.82 1.98 1.98 1.97 1.97 1.96 1.96 1.95 1.94
Ph
O
N
O
1c
1H NMR (400 MHz, CDCl3)
1.00 1.02 1.04 1.03 2.03 3.03 1.02 2.03 2.06 1.04 4.01
9.5 9.0 8.5 8.0 7.5 7.0 6.5 6.0 5.5 5.0 4.5 4.0 3.5 3.0 2.5 2.0 1.5 1.0 0.5
ppm

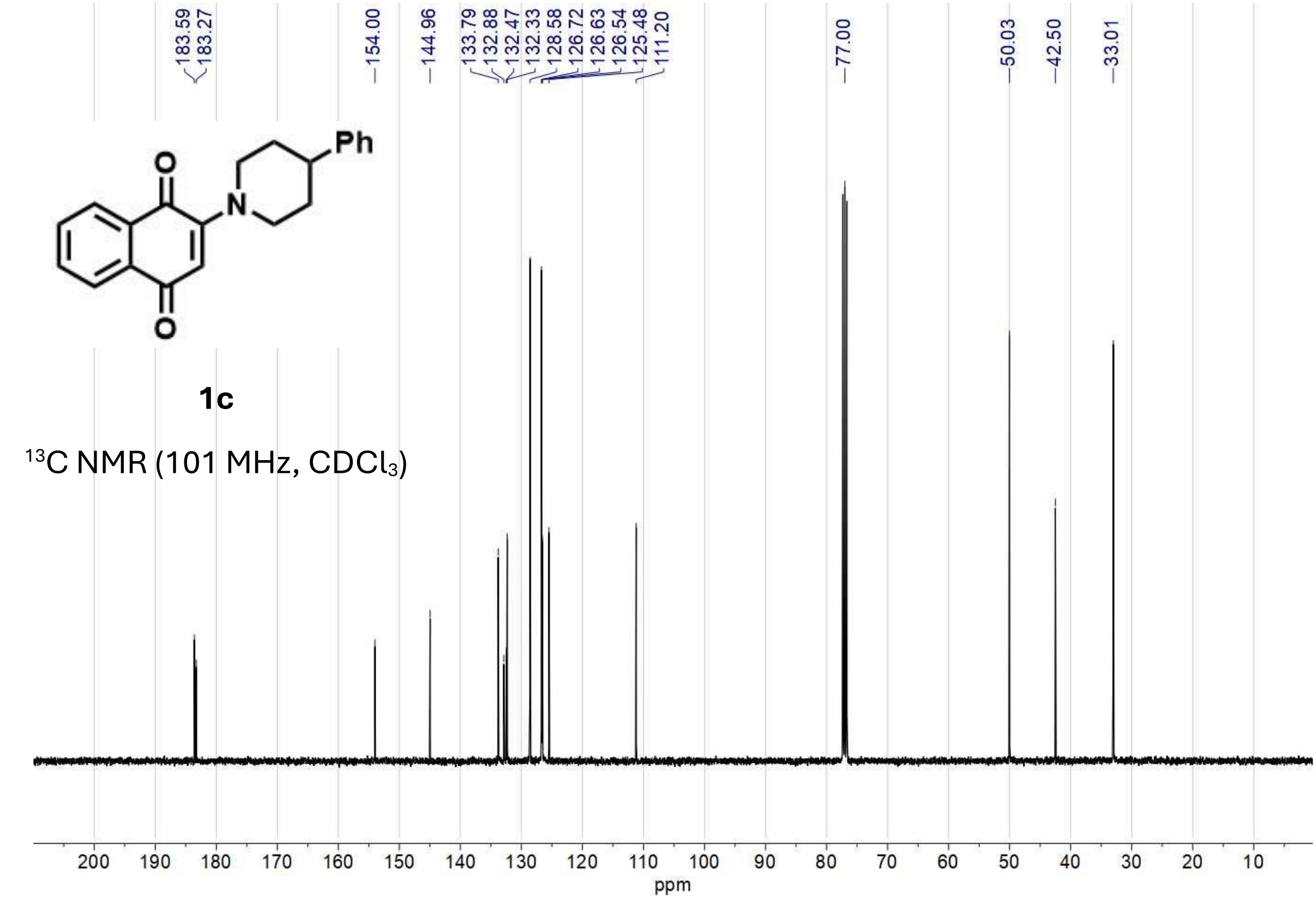
183.59 183.27 154.00 144.96 133.79 132.88 132.47 132.33 128.58 126.72 126.63 126.54 125.48 111.20 77.00 50.03 42.50 33.01
Ph
O
N
O
1c
13C NMR (101 MHz, CDCl3)
200 190 180 170 160 150 140 130 120 110 100 90 80 70 60 50 40 30 20 10
ppm

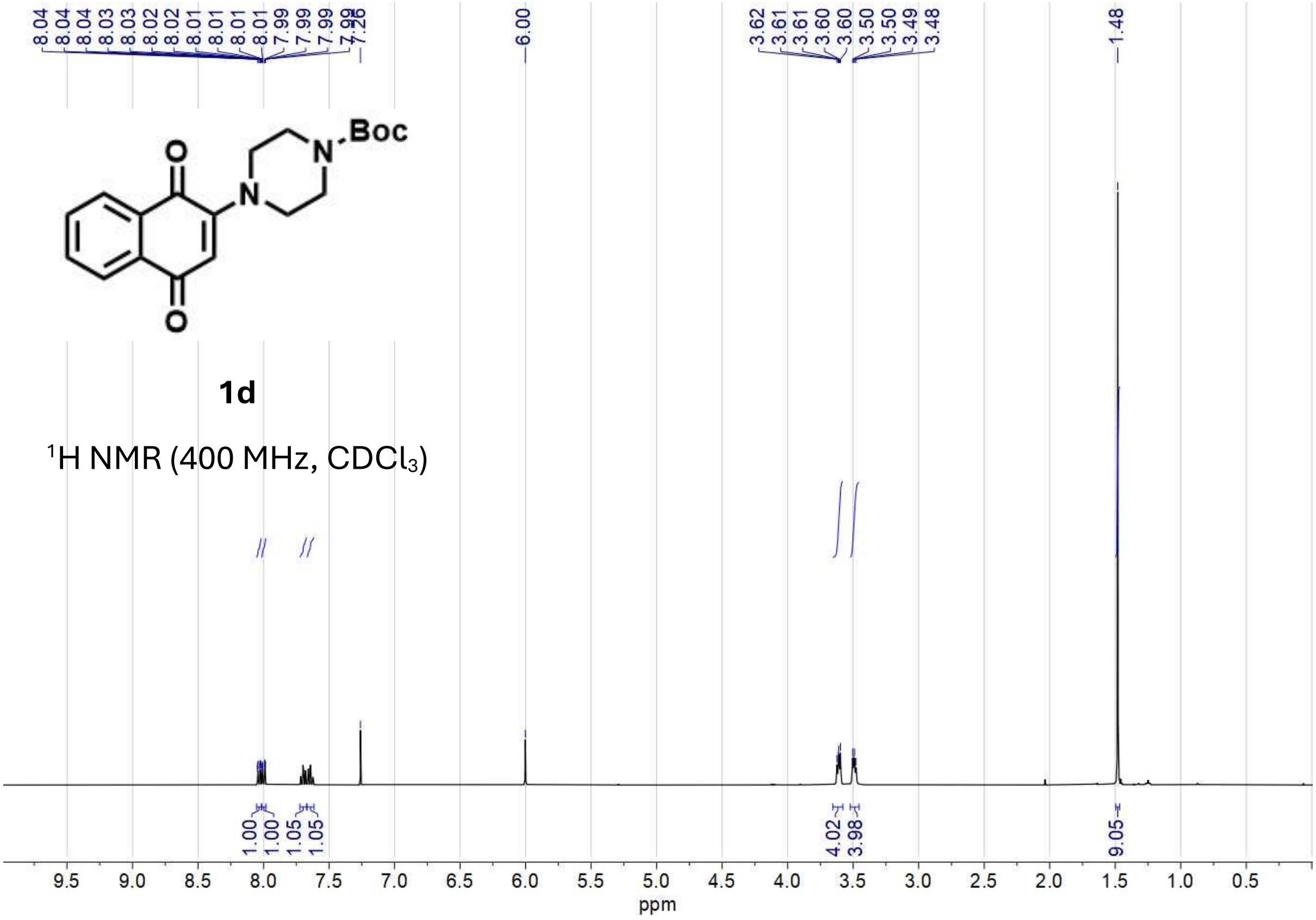

Boc
1d
1H NMR (400 MHz, CDCl3)
ppm

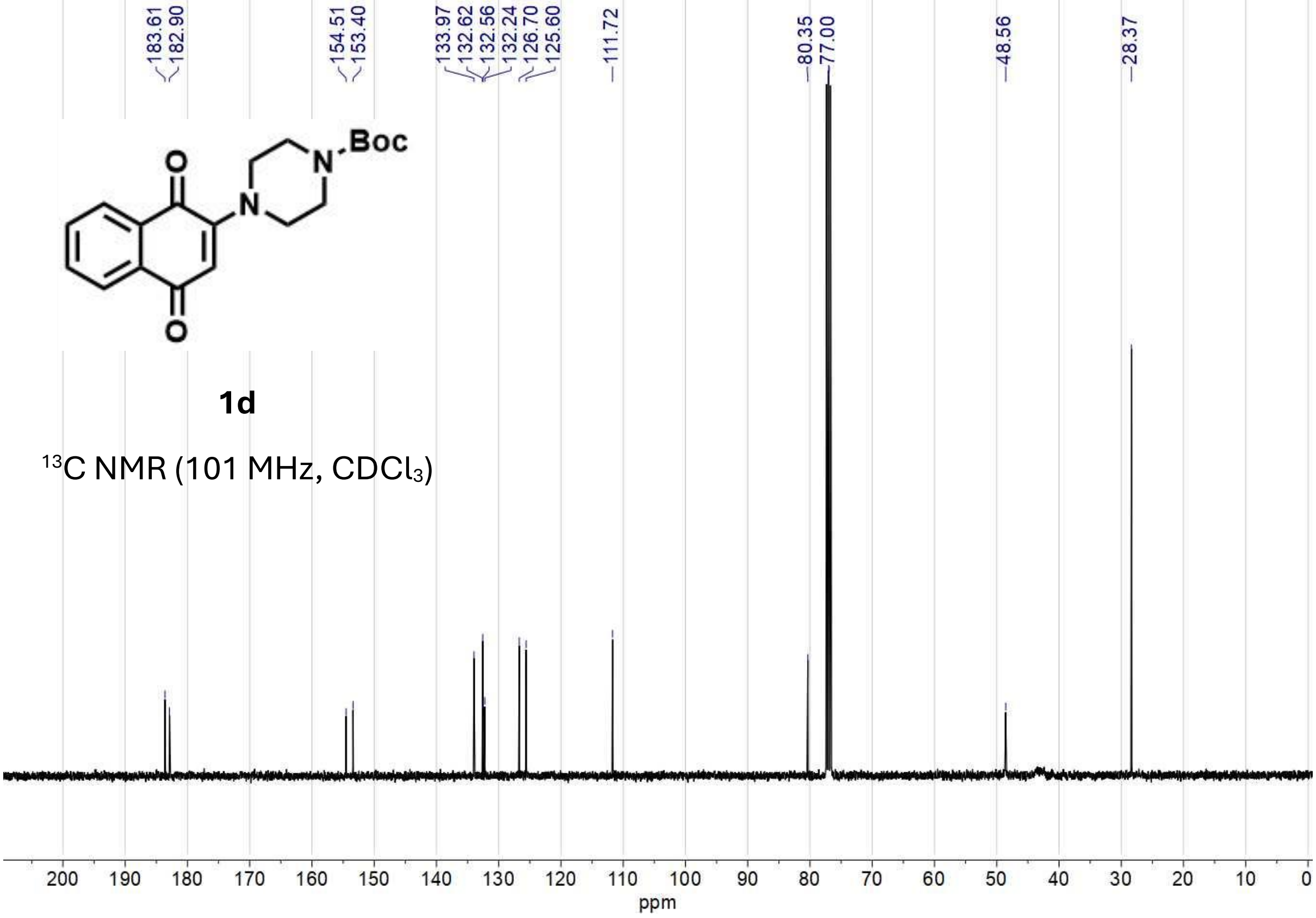

Boc
1d
13C NMR (101 MHz, CDCl3)
ppm

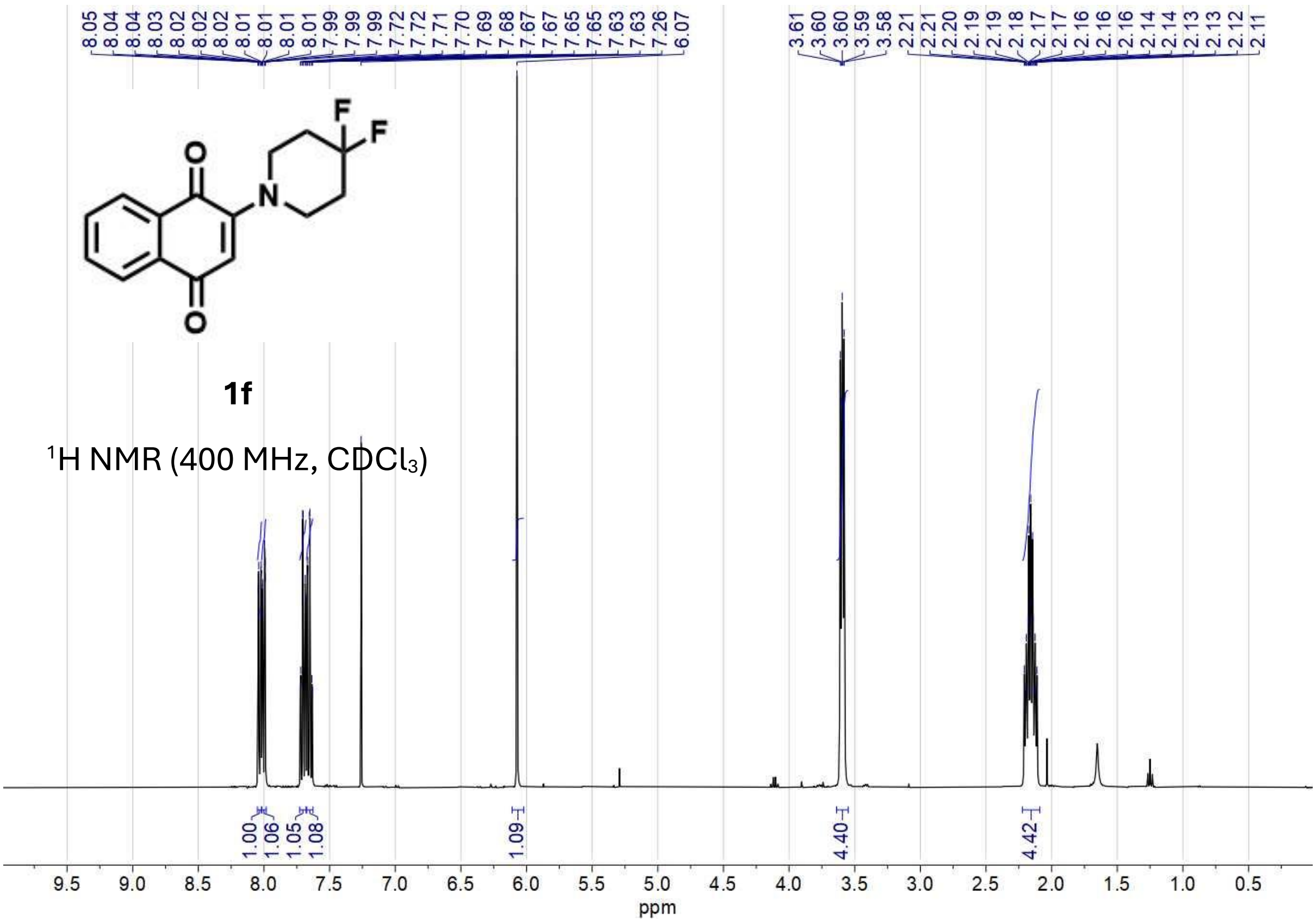
8.05 8.04 8.04 8.03 8.02 8.02 8.02 8.01 8.01 8.01 8.01 7.99 7.99 7.72 7.72 7.71 7.70 7.69 7.68 7.67 7.67 7.65 7.65 7.63 7.63 7.26 6.07
3.61 3.60 3.60 3.59 3.58 2.21 2.21 2.20 2.19 2.19 2.18 2.17 2.17 2.16 2.16 2.16 2.14 2.14 2.13 2.13 2.12 2.11
F
F
O
N
O
1f
1H NMR (400 MHz, CDCl3)
1.00 1.06 1.05 1.08 1.09 4.40 4.42
9.5 9.0 8.5 8.0 7.5 7.0 6.5 6.0 5.5 5.0 4.5 4.0 3.5 3.0 2.5 2.0 1.5 1.0 0.5
ppm

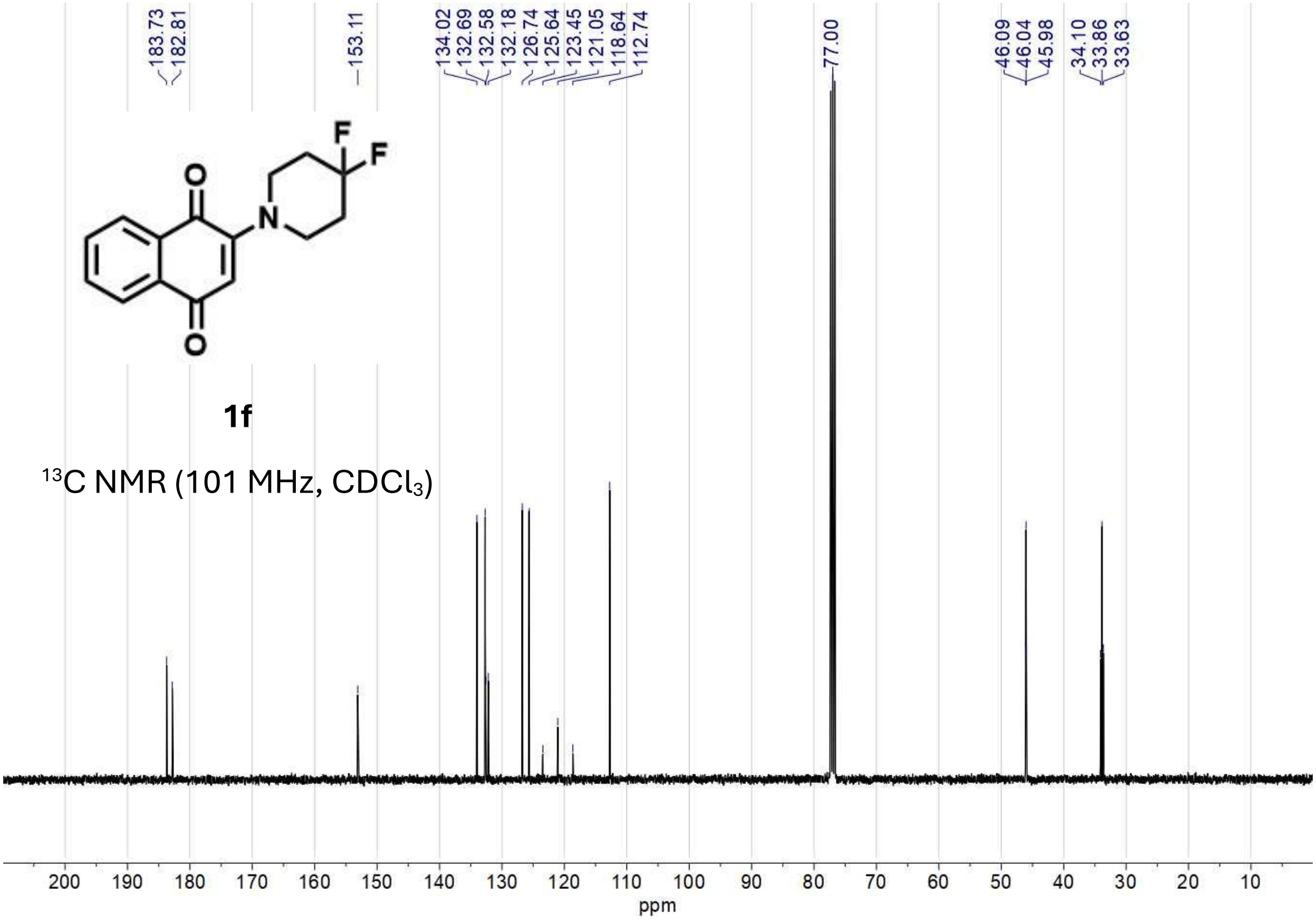
183.73 182.81 153.11 134.02 132.69 132.58 132.18 126.74 125.64 123.45 121.05 118.64 112.74 77.00 46.09 46.04 45.98 34.10 33.86 33.63
F
F
O
N
O
1f
13C NMR (101 MHz, CDCl3)
200 190 180 170 160 150 140 130 120 110 100 90 80 70 60 50 40 30 20 10
ppm

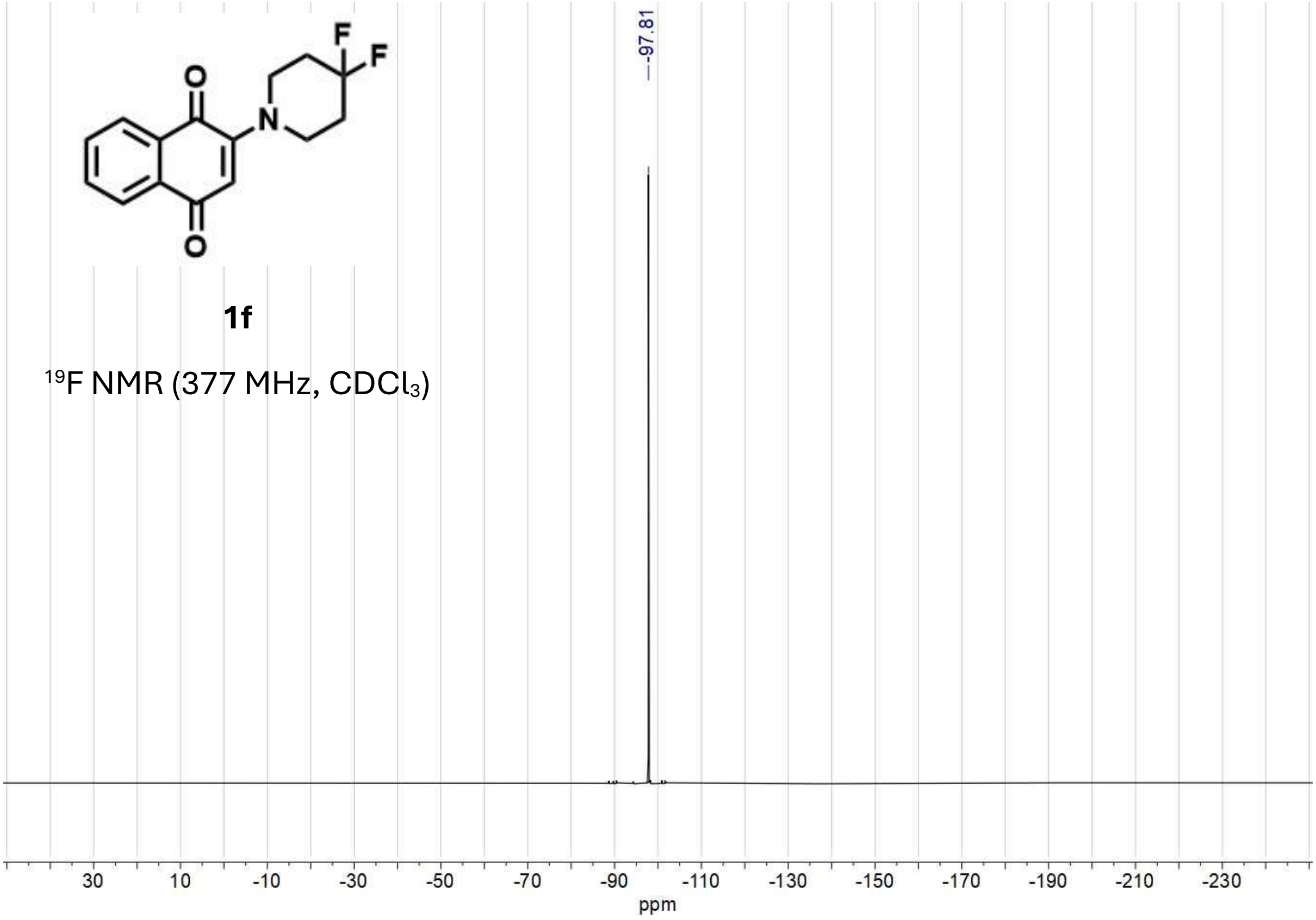

1f

^{19}F NMR (377 MHz, $CDCl_3$)

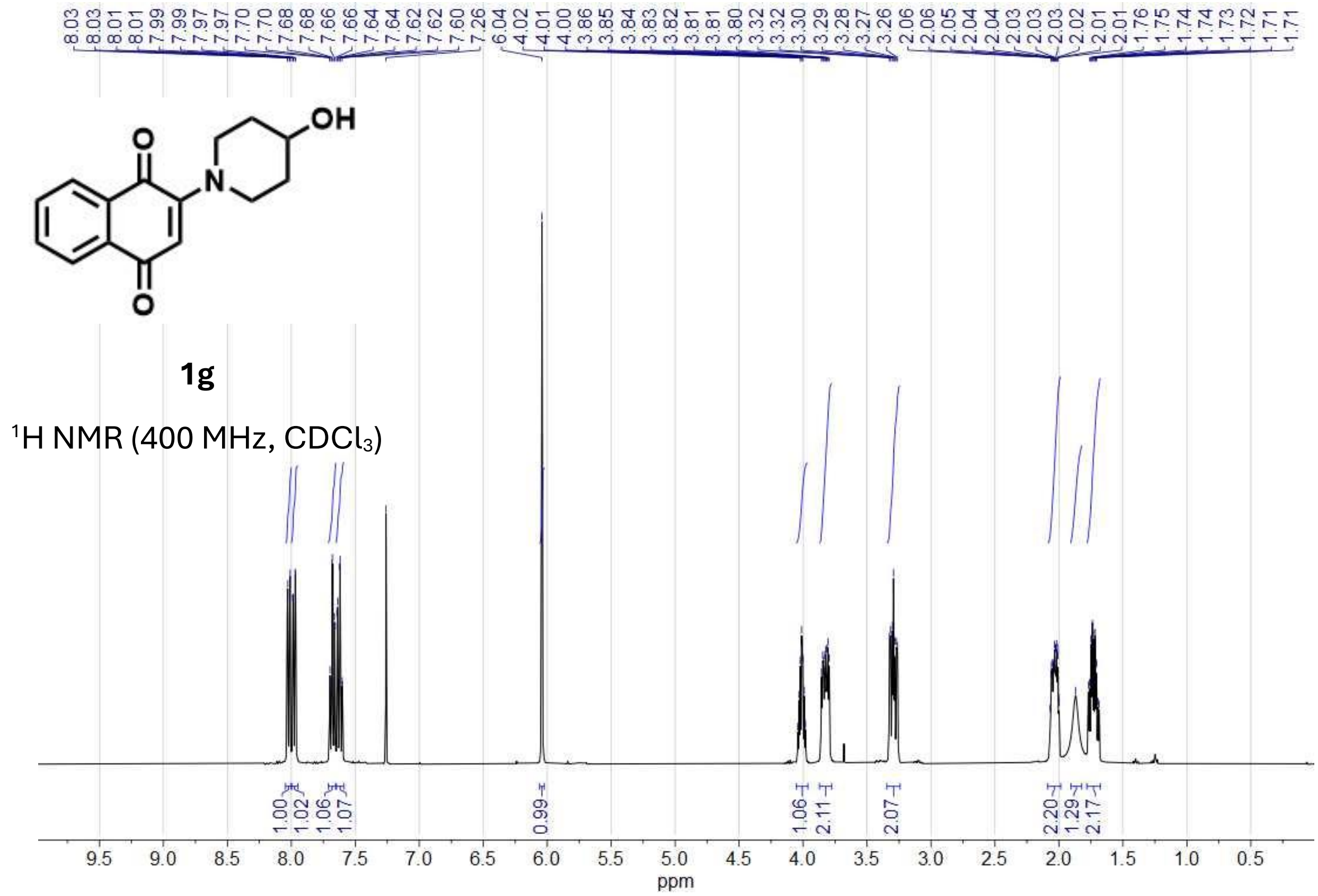

1g

^{1}H NMR (400 MHz, $CDCl_3$)

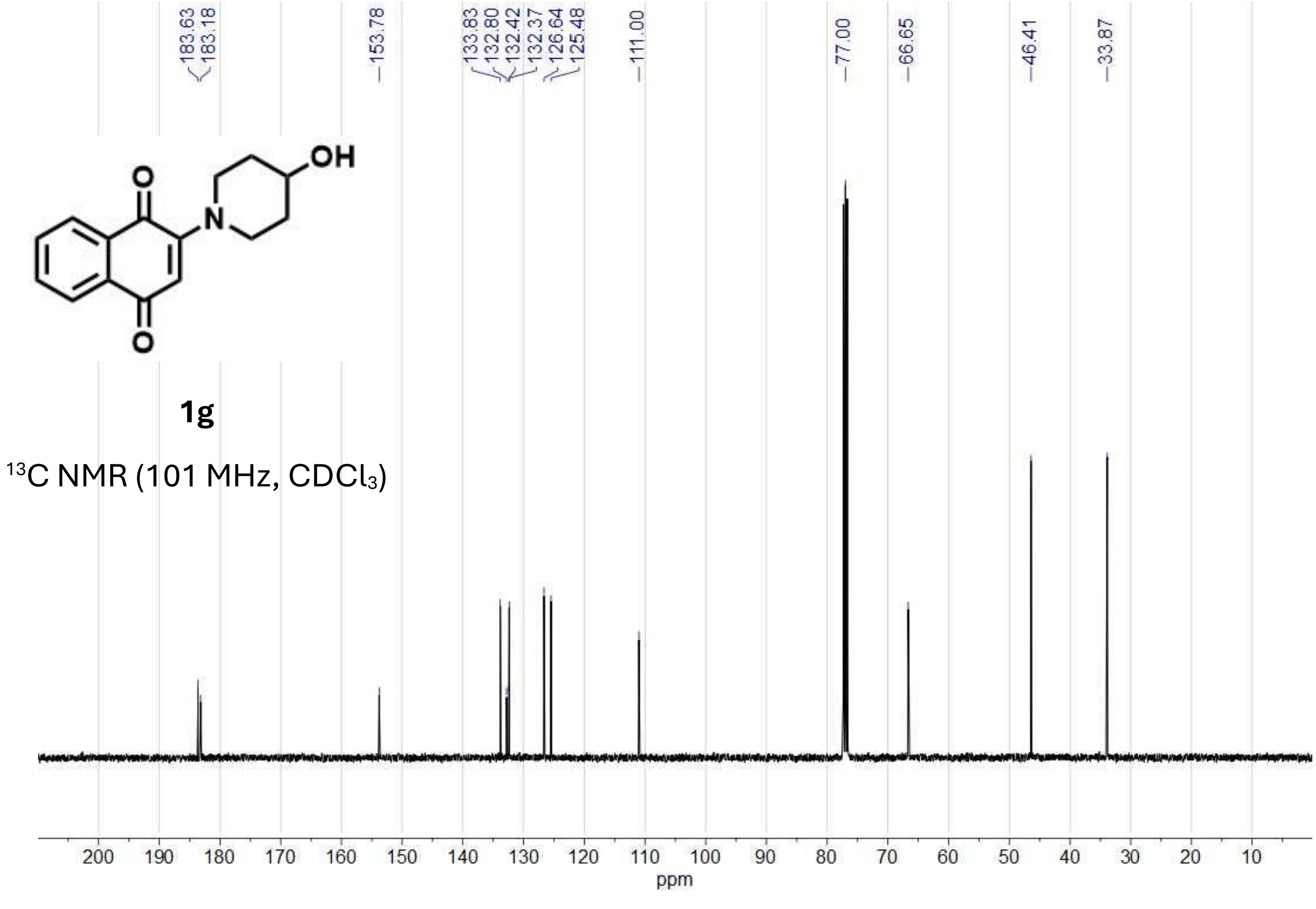

1g

^{13}C NMR (101 MHz, $CDCl_3$)

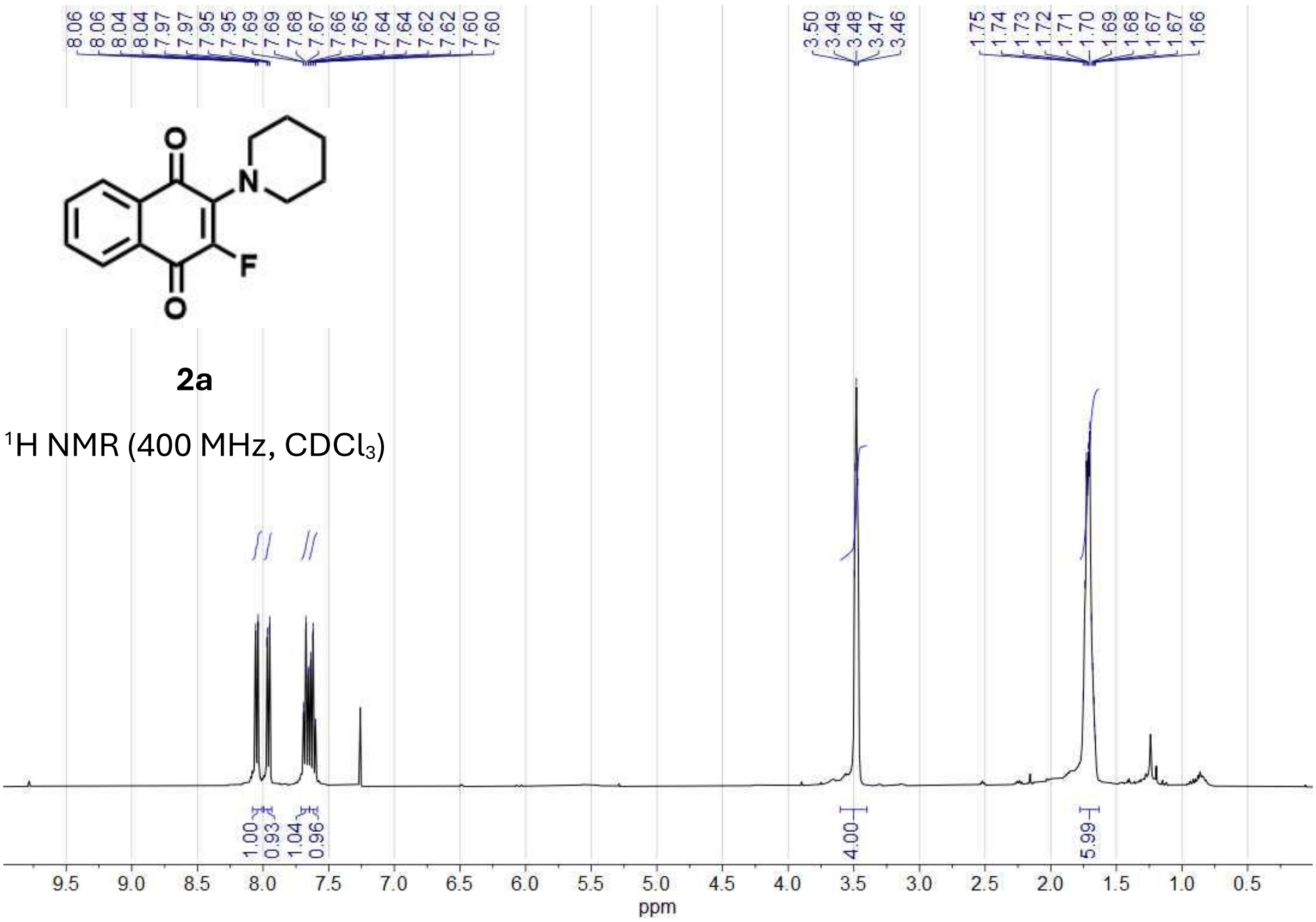
8.06 8.06 8.04 8.04 7.97 7.97 7.95 7.95 7.69 7.69 7.68 7.67 7.66 7.65 7.64 7.64 7.62 7.62 7.60 7.60
3.50 3.49 3.48 3.47 3.46
1.75 1.74 1.73 1.72 1.71 1.70 1.69 1.68 1.67 1.67 1.66
O
N
F
O
2a
^{1}H NMR (400 MHz, CDCl$_3$)
1.00 0.93 1.04 0.96
4.00
5.99
9.5 9.0 8.5 8.0 7.5 7.0 6.5 6.0 5.5 5.0 4.5 4.0 3.5 3.0 2.5 2.0 1.5 1.0 0.5
ppm

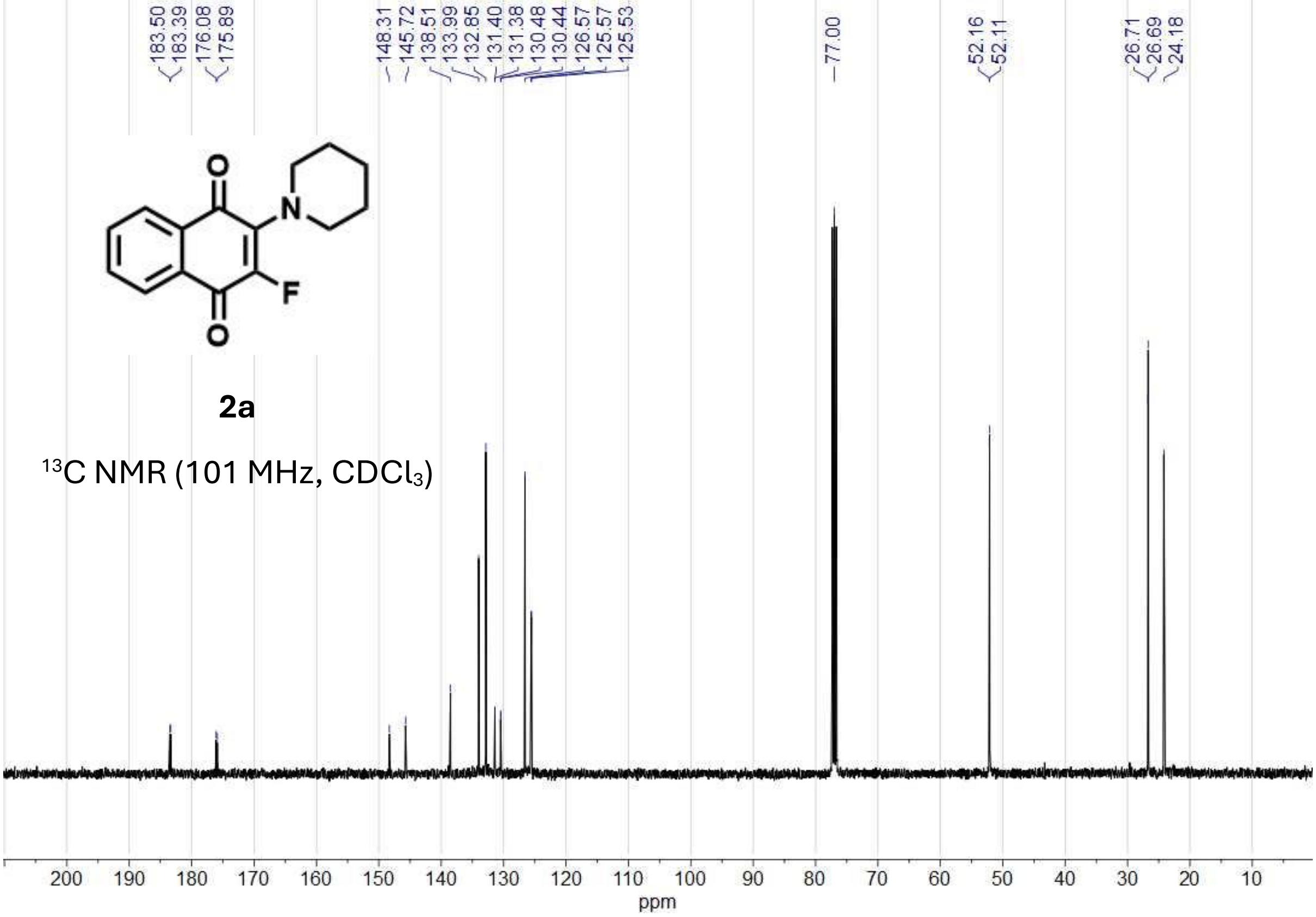
183.50 183.39 176.08 175.89
148.31 145.72 138.51 133.99 132.85 131.40 131.38 130.48 130.44 126.57 125.57 125.53
77.00
52.16 52.11
26.71 26.69 24.18
O
N
F
O
2a
^{13}C NMR (101 MHz, CDCl$_3$)
200 190 180 170 160 150 140 130 120 110 100 90 80 70 60 50 40 30 20 10
ppm

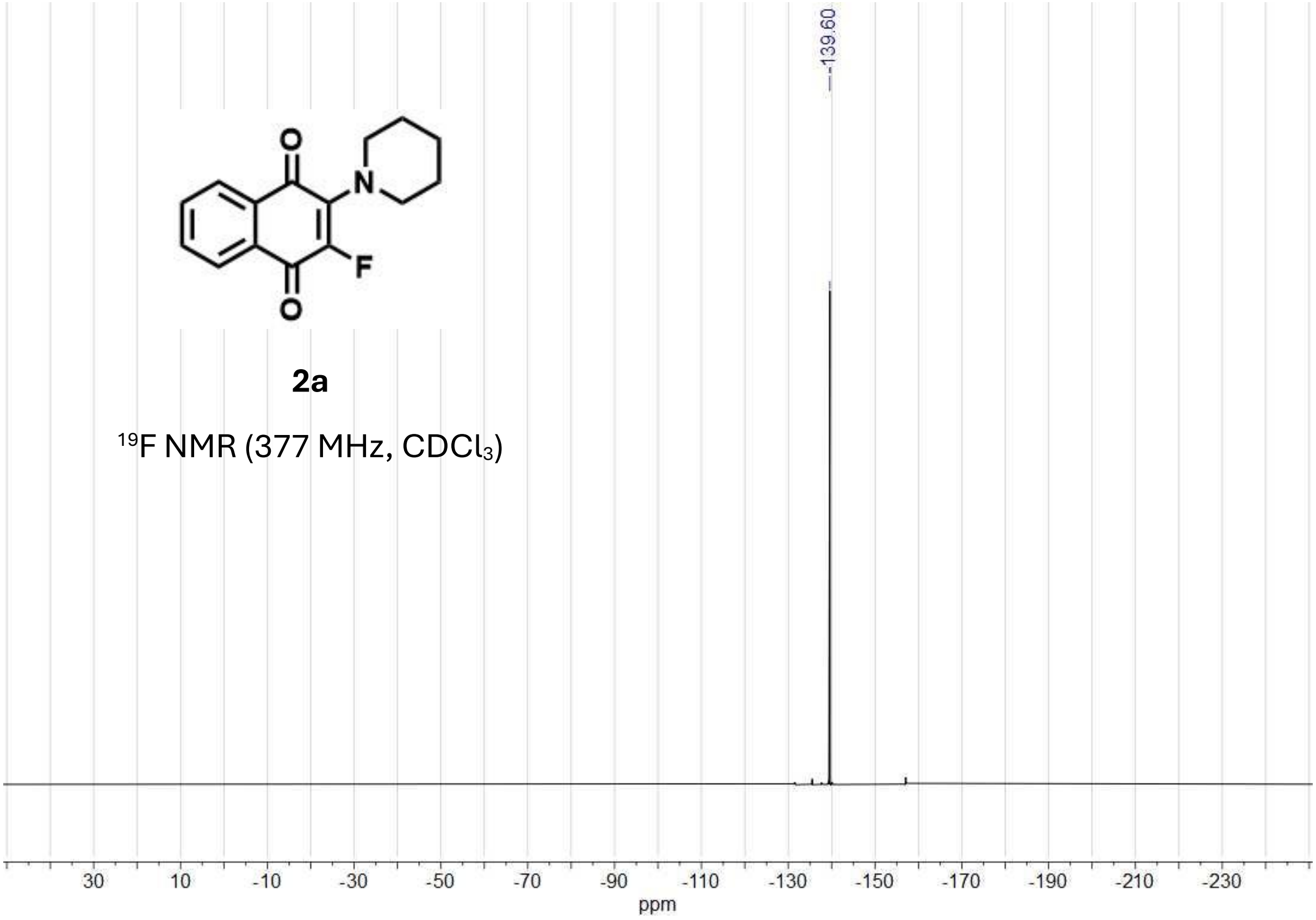

2a

^{19}F NMR (377 MHz, $CDCl_3$)

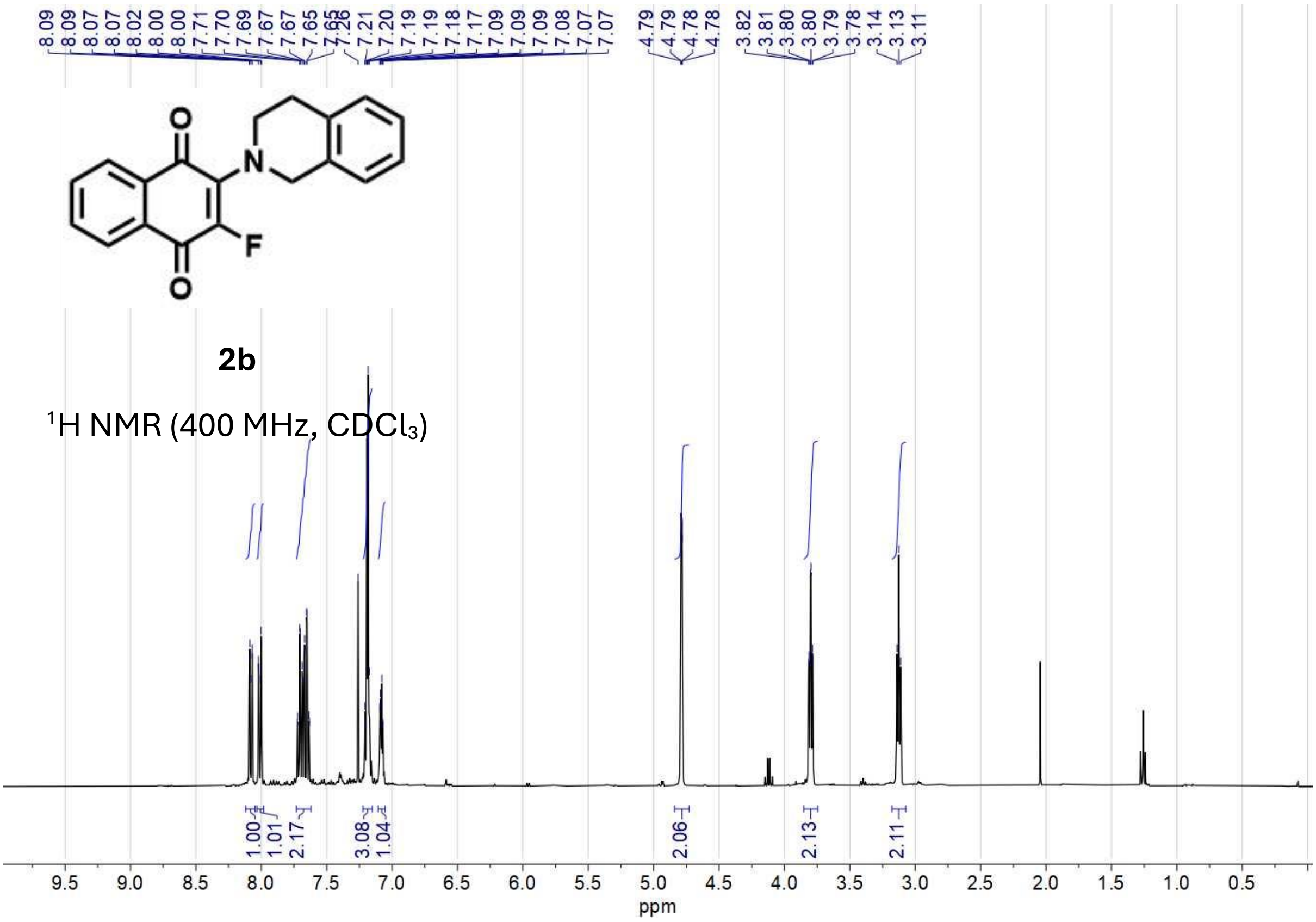

2b

^{1}H NMR (400 MHz, $CDCl_3$)

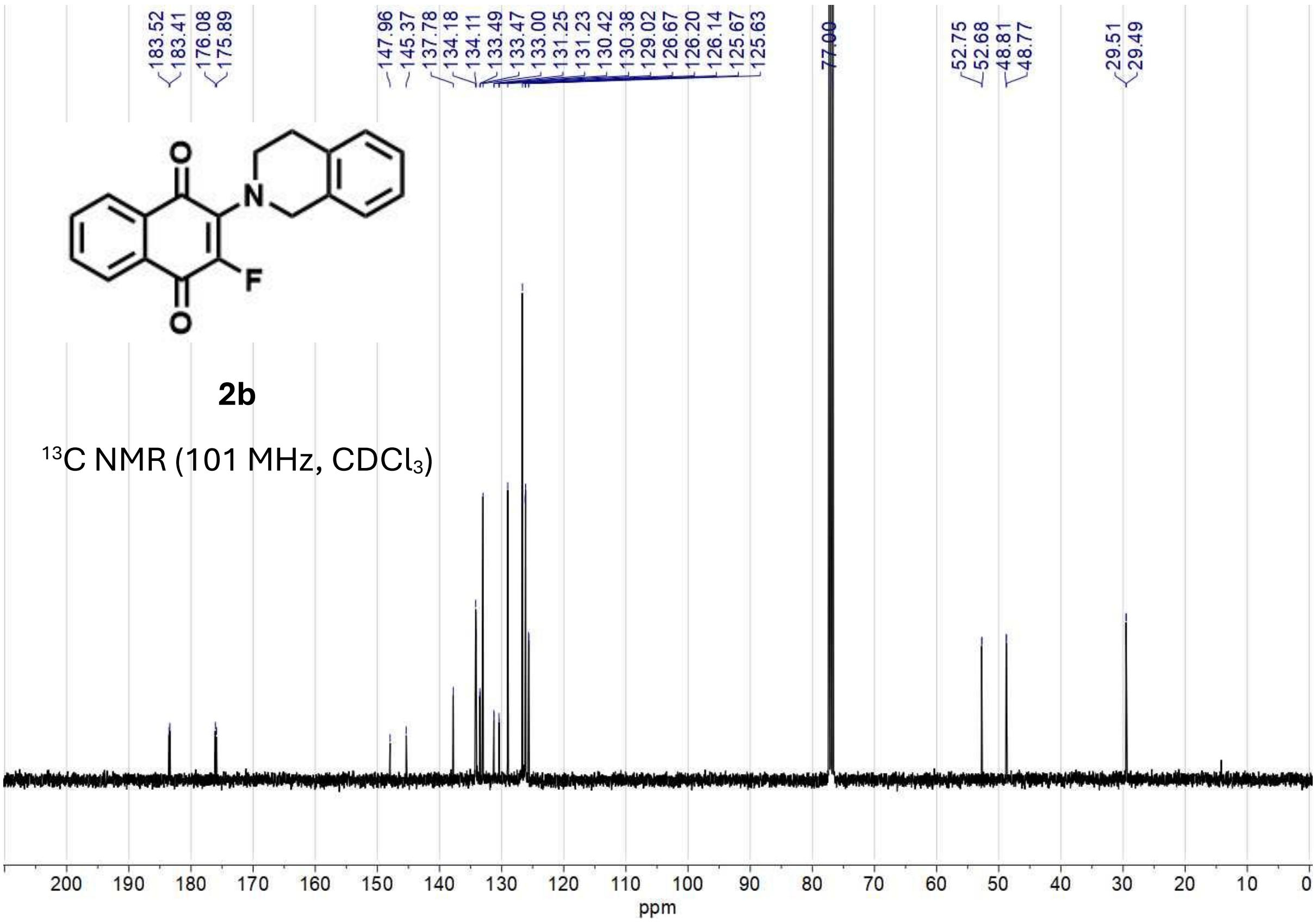

2b

^{13}C NMR (101 MHz, $CDCl_3$)

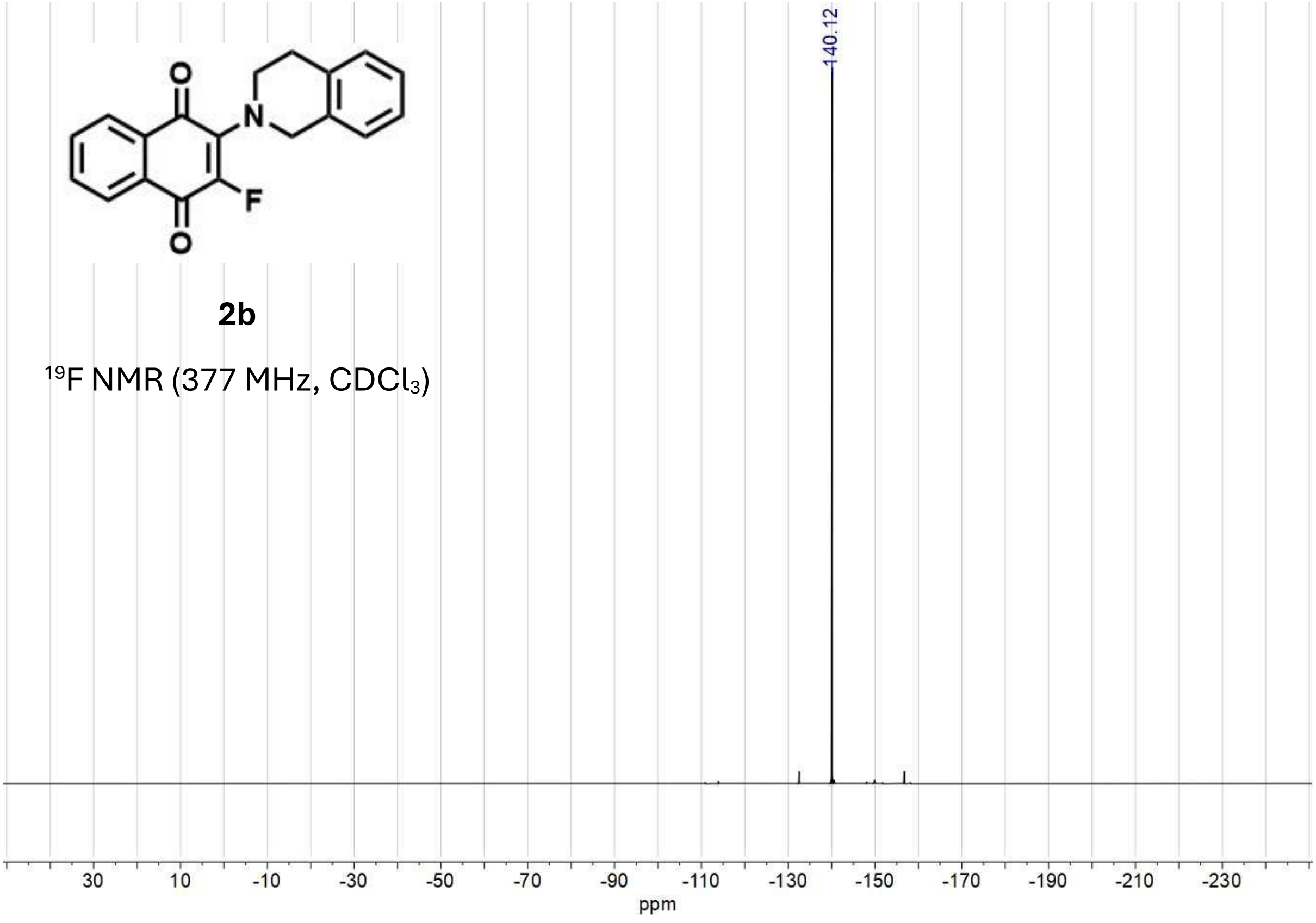

2b

^{19}F NMR (377 MHz, $CDCl_3$)

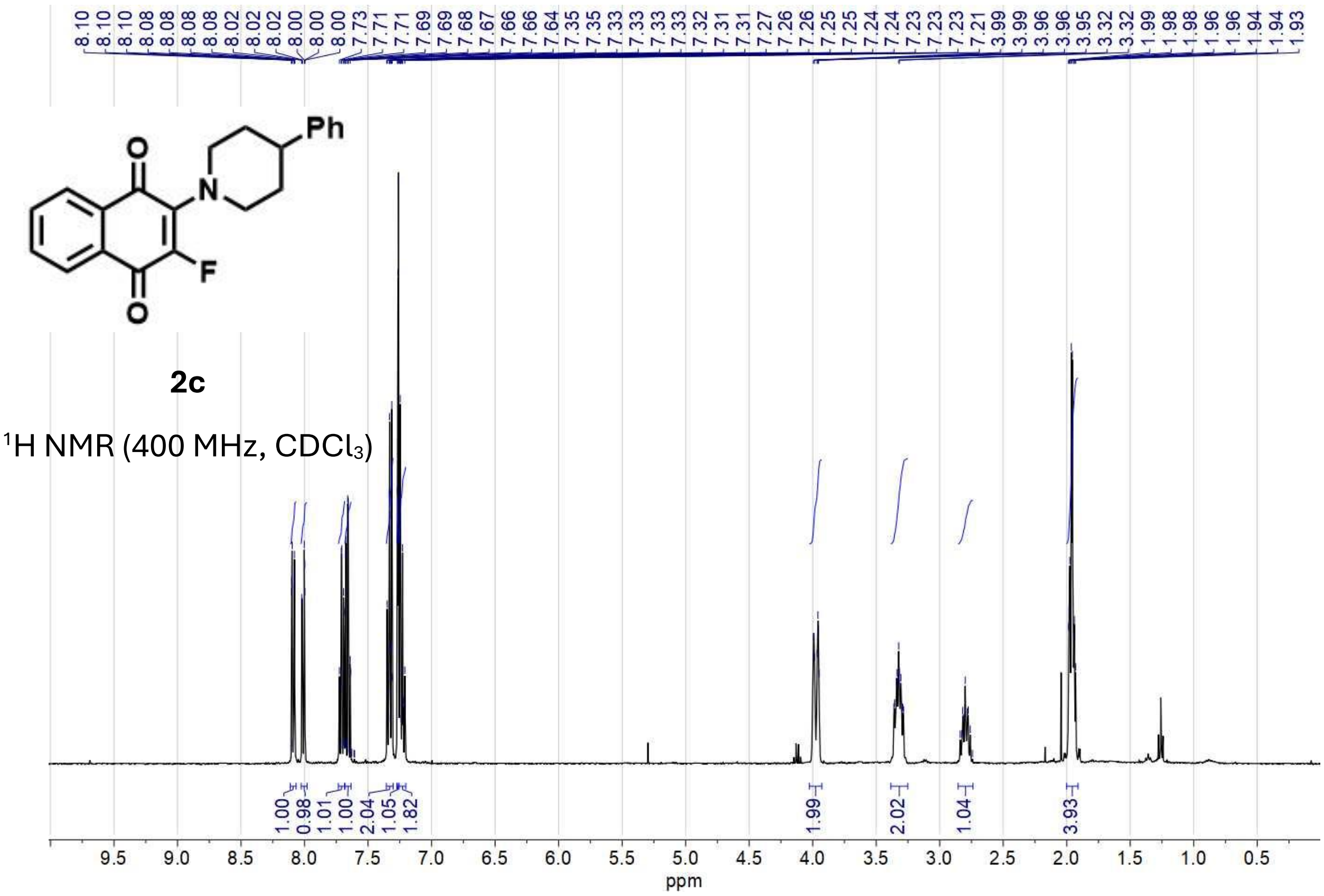

2c

^{1}H NMR (400 MHz, $CDCl_3$)

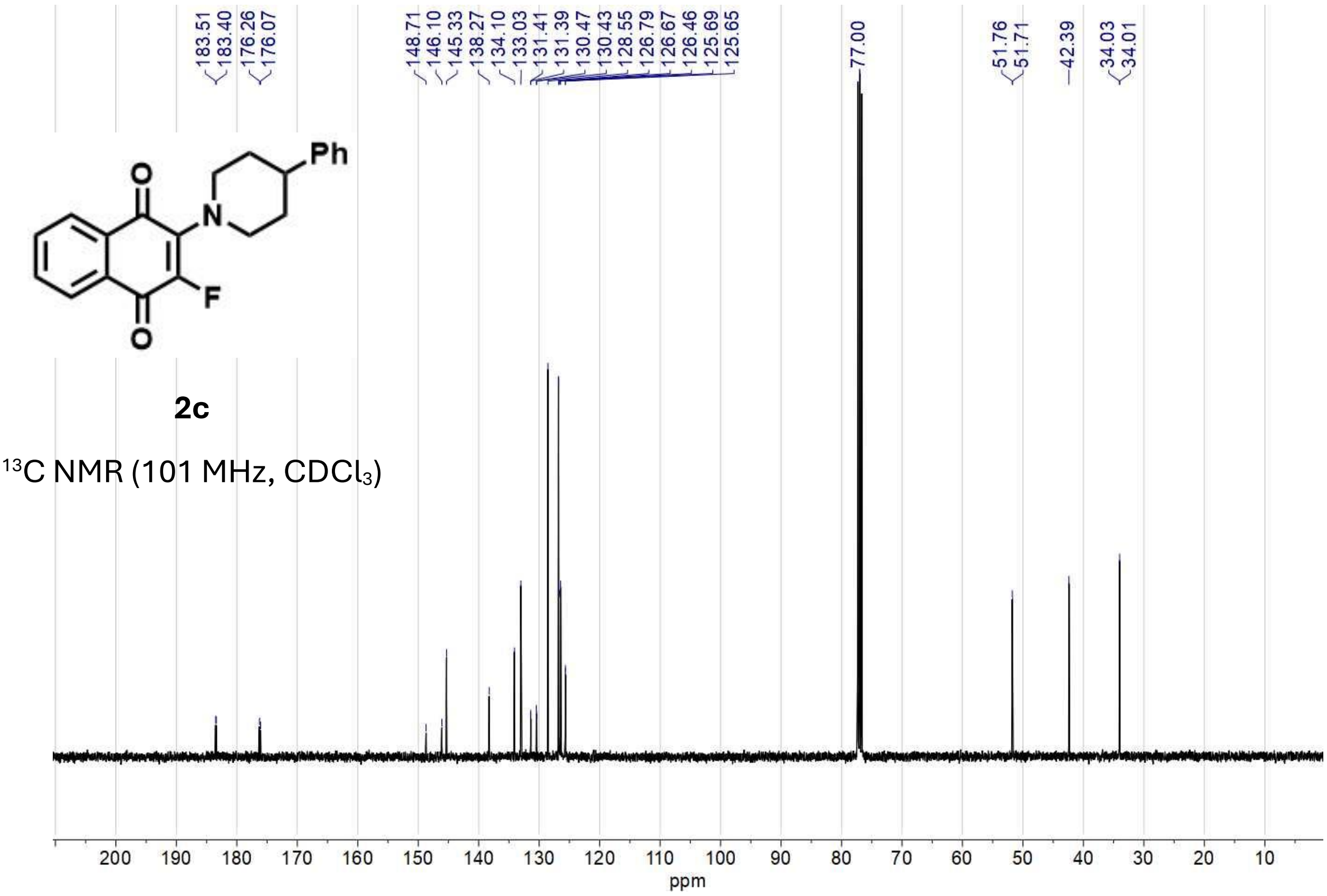

2c

^{13}C NMR (101 MHz, $CDCl_3$)

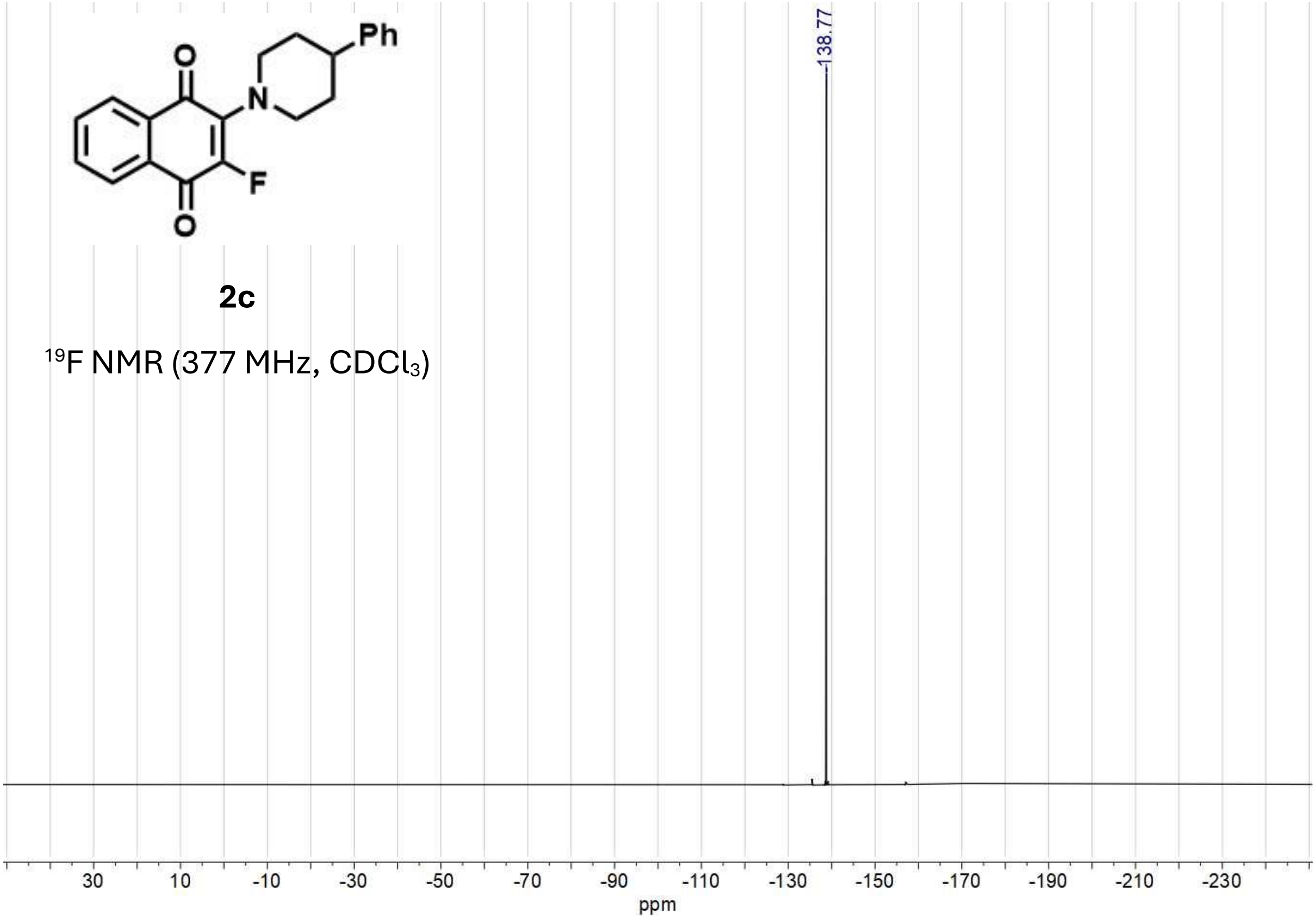

2c

^{19}F NMR (377 MHz, $CDCl_3$)

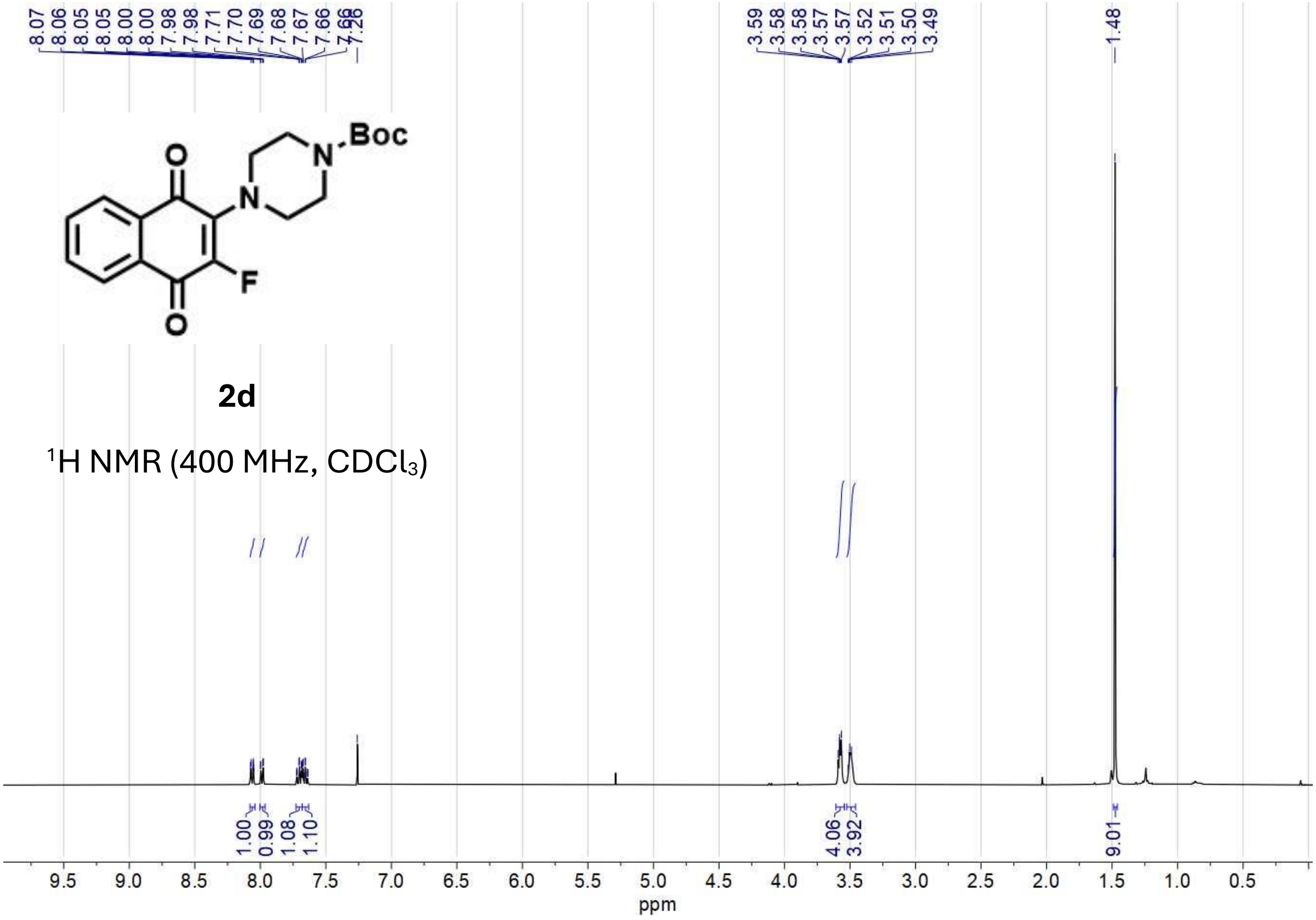

2d

^{1}H NMR (400 MHz, $CDCl_3$)

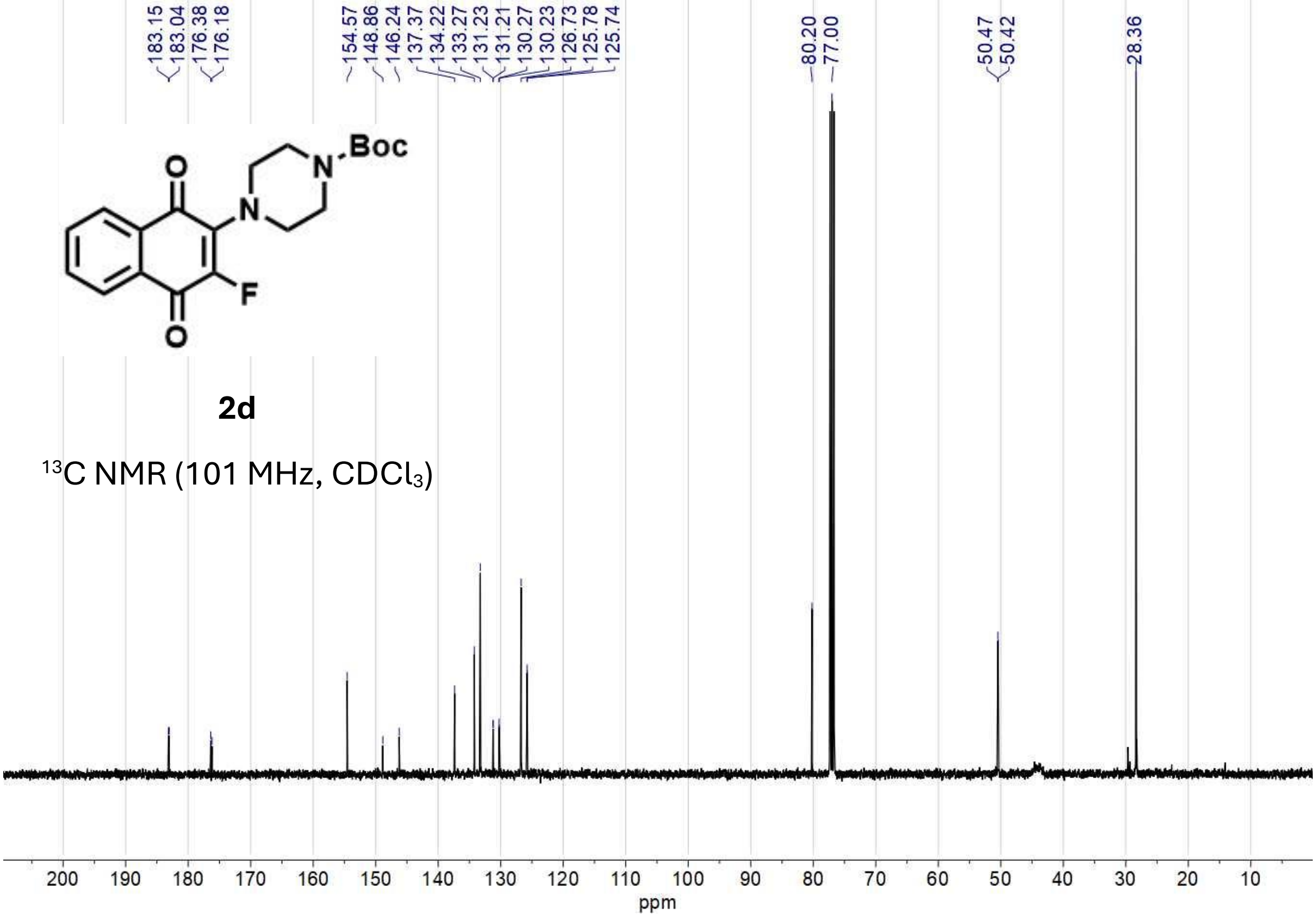

2d

^{13}C NMR (101 MHz, $CDCl_3$)

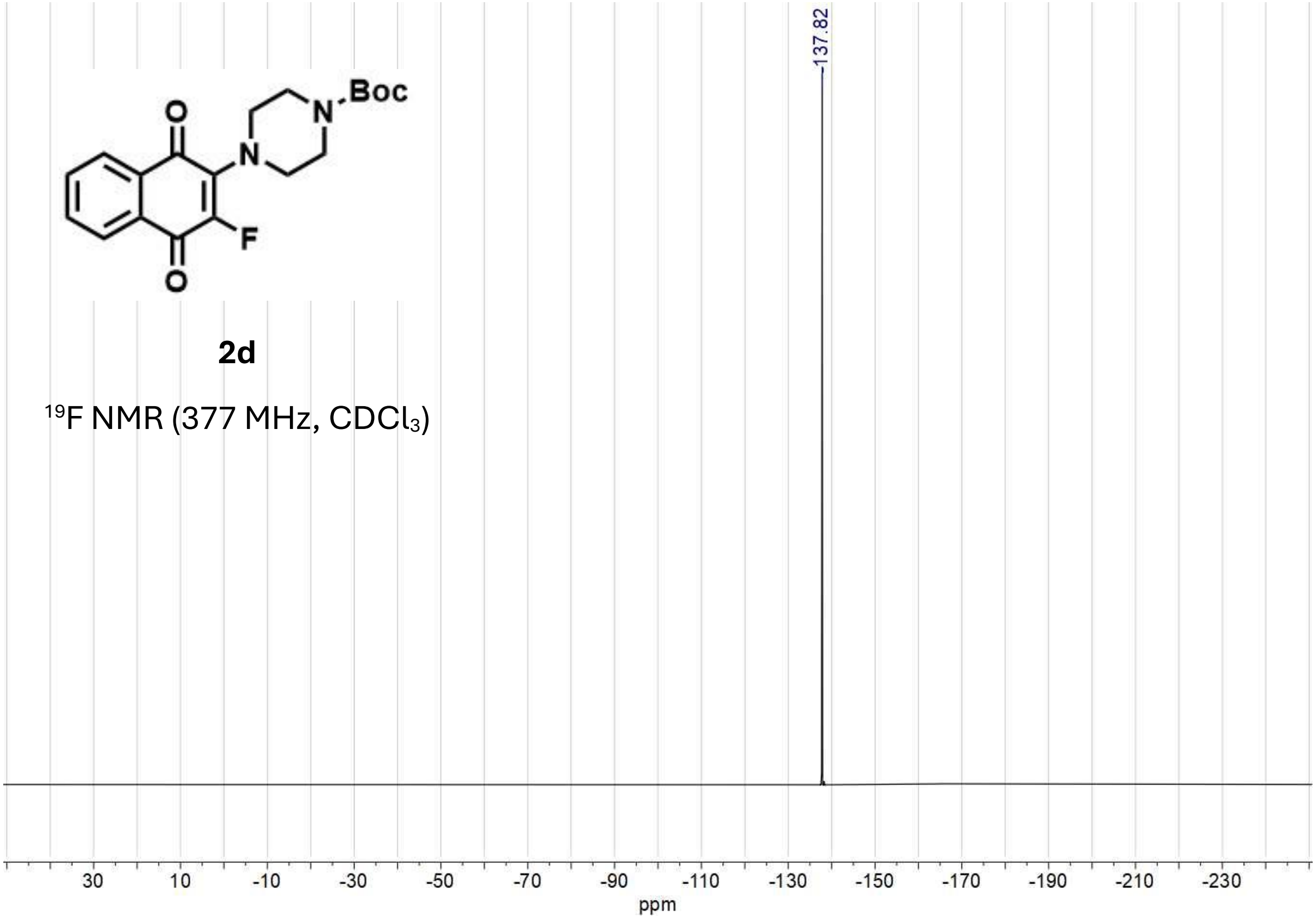

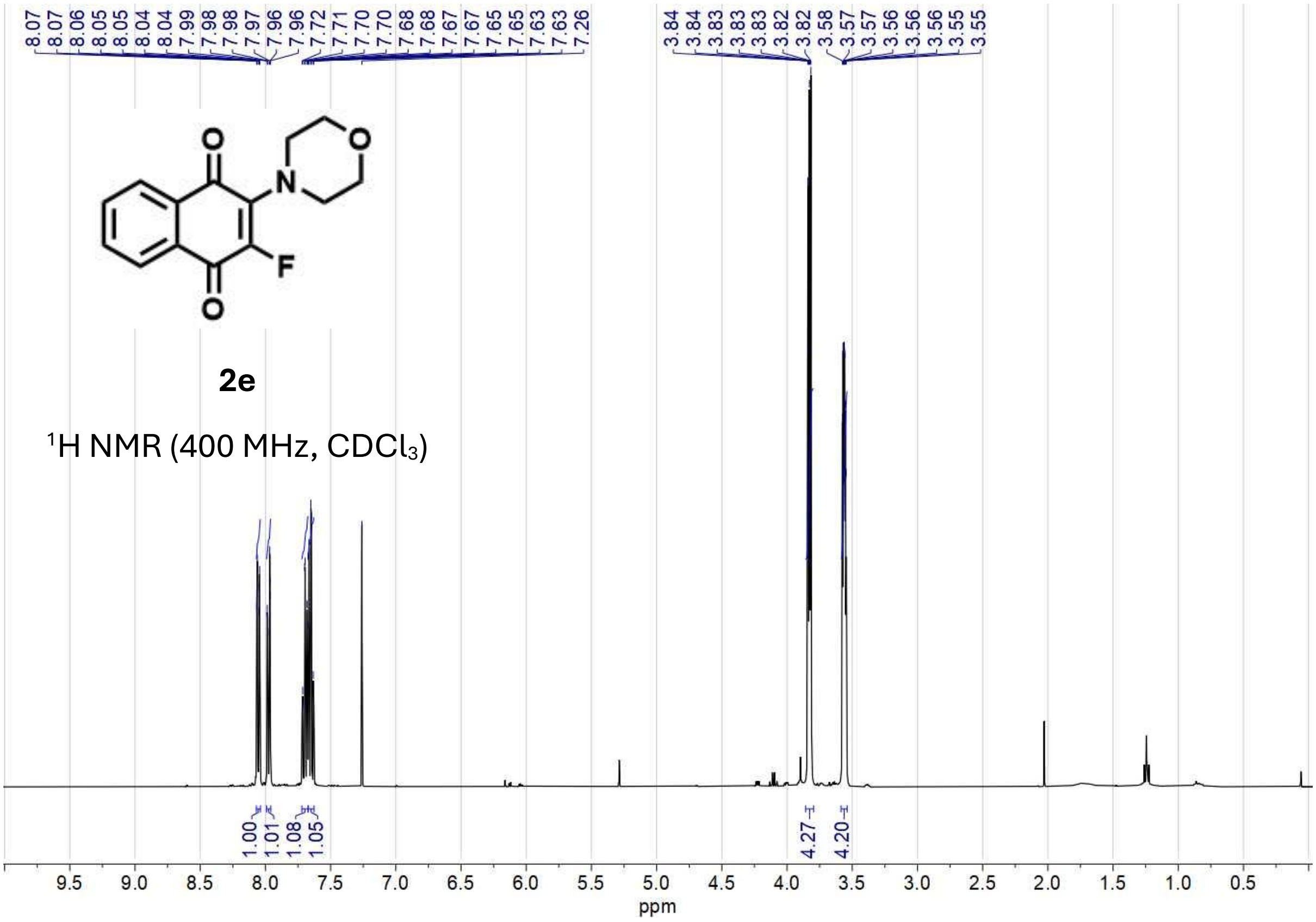
8.07 8.07 8.06 8.05 8.05 8.04 8.04 7.99 7.98 7.98 7.97 7.96 7.96 7.72 7.71 7.70 7.70 7.68 7.68 7.67 7.65 7.65 7.63 7.63 7.26
3.84 3.84 3.83 3.83 3.83 3.82 3.82 3.58 3.57 3.57 3.56 3.56 3.55 3.55
O
N
O
F
O
2e
^{1}H NMR (400 MHz, $CDCl_3$)
1.00 1.01 1.08 1.05
4.27 4.20
9.5 9.0 8.5 8.0 7.5 7.0 6.5 6.0 5.5 5.0 4.5 4.0 3.5 3.0 2.5 2.0 1.5 1.0 0.5
ppm

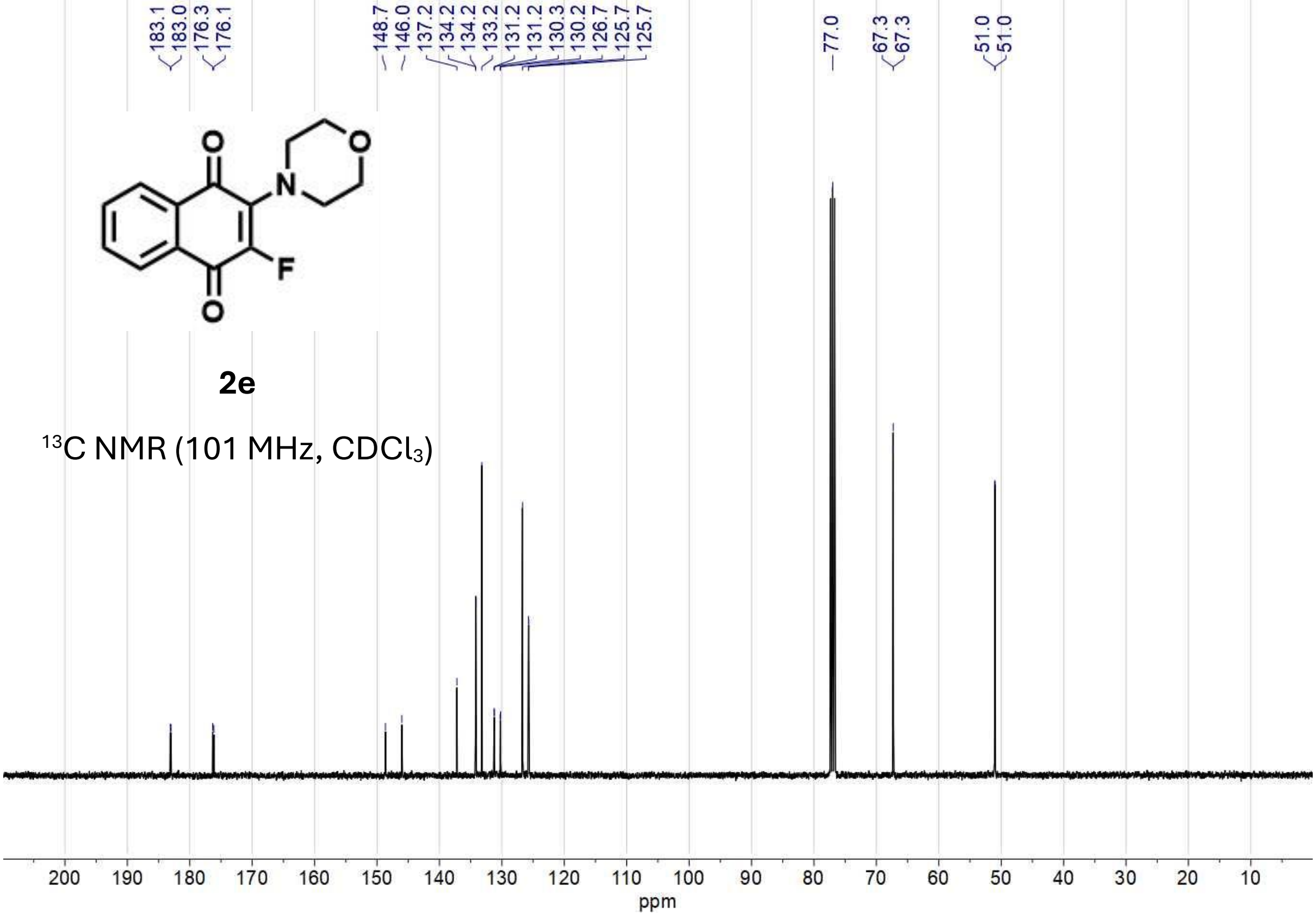
183.1 183.0 176.3 176.1
148.7 146.0 137.2 134.2 134.2 133.2 131.2 131.2 130.3 130.2 126.7 125.7
77.0
67.3 67.3
51.0 51.0
O
N
O
F
O
2e
^{13}C NMR (101 MHz, $CDCl_3$)
200 190 180 170 160 150 140 130 120 110 100 90 80 70 60 50 40 30 20 10
ppm

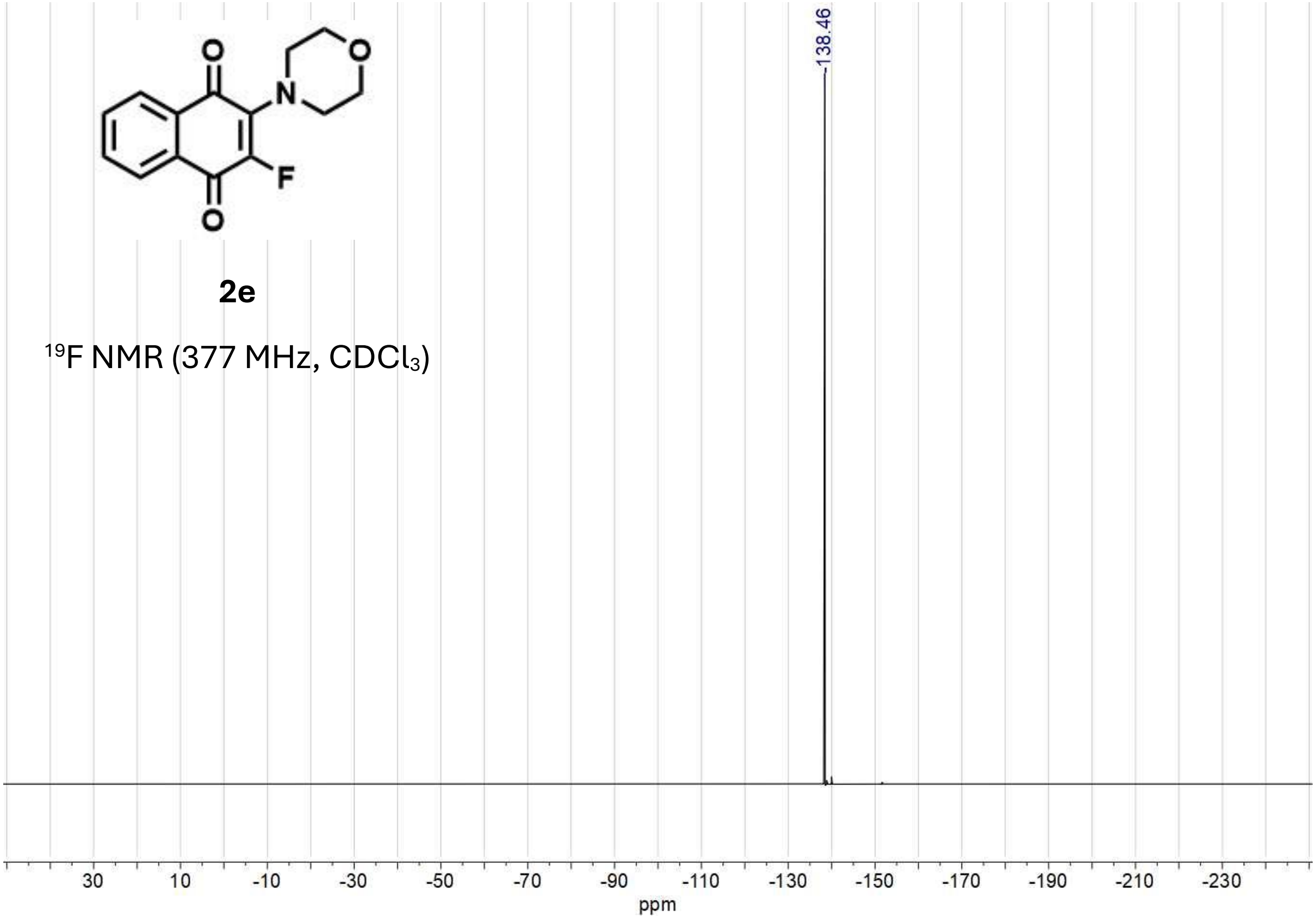

-138.46
O
N
O
F
O
2e
^{19}F NMR (377 MHz, $CDCl_3$)
30
10
-10
-30
-50
-70
-90
-110
-130
-150
-170
-190
-210
-230
ppm

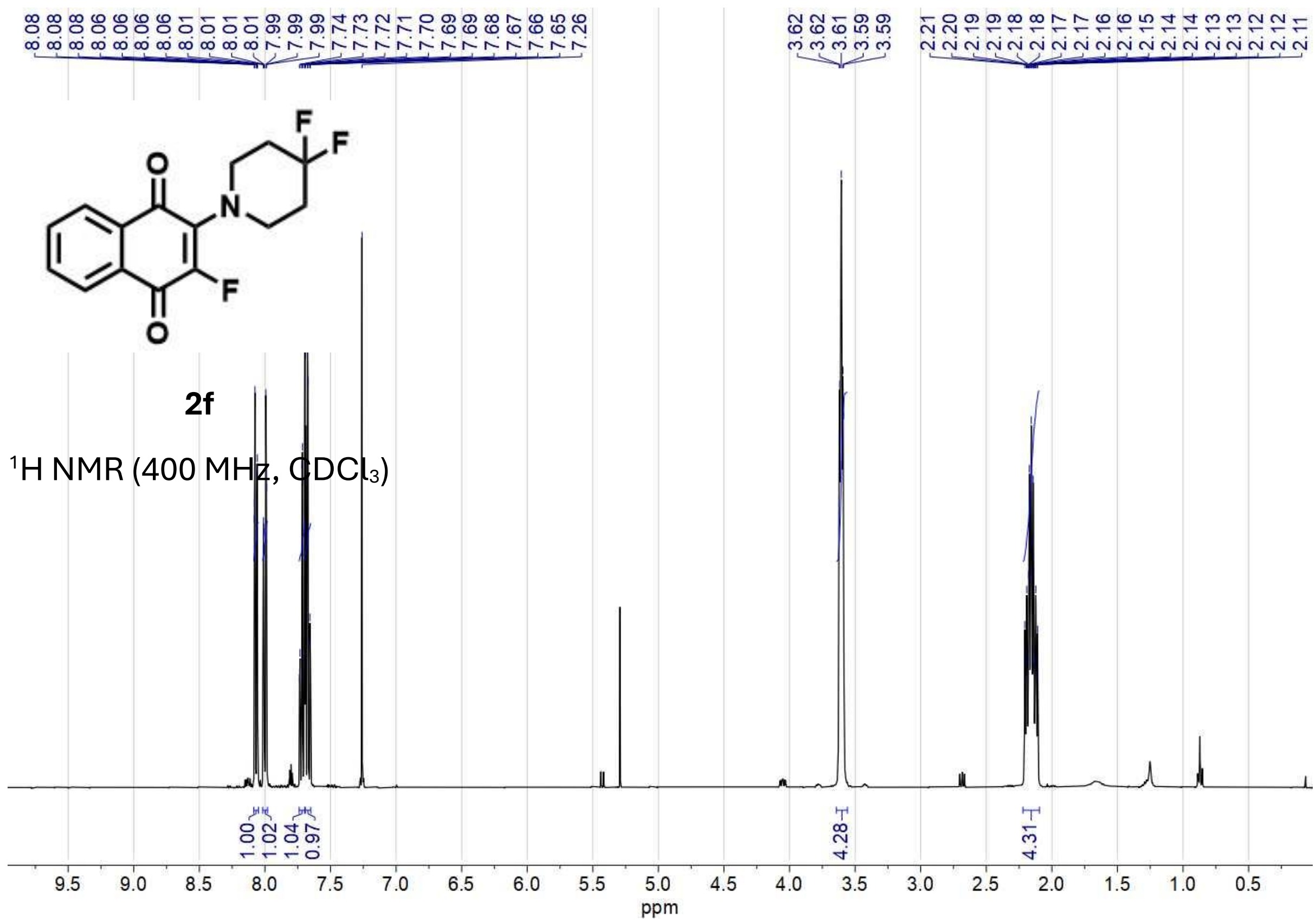
8.08 8.08 8.08 8.06 8.06 8.06 8.01 8.01 8.01 8.01 7.99 7.99 7.74 7.73 7.72 7.71 7.70 7.69 7.68 7.67 7.66 7.65 7.26
3.62 3.62 3.61 3.59 3.59
2.21 2.20 2.19 2.19 2.18 2.18 2.17 2.17 2.16 2.16 2.15 2.14 2.14 2.13 2.13 2.12 2.12 2.11
2f
^{1}H NMR (400 MHz, $CDCl_3$)
1.00 1.02 1.04 0.97
4.28
4.31
ppm

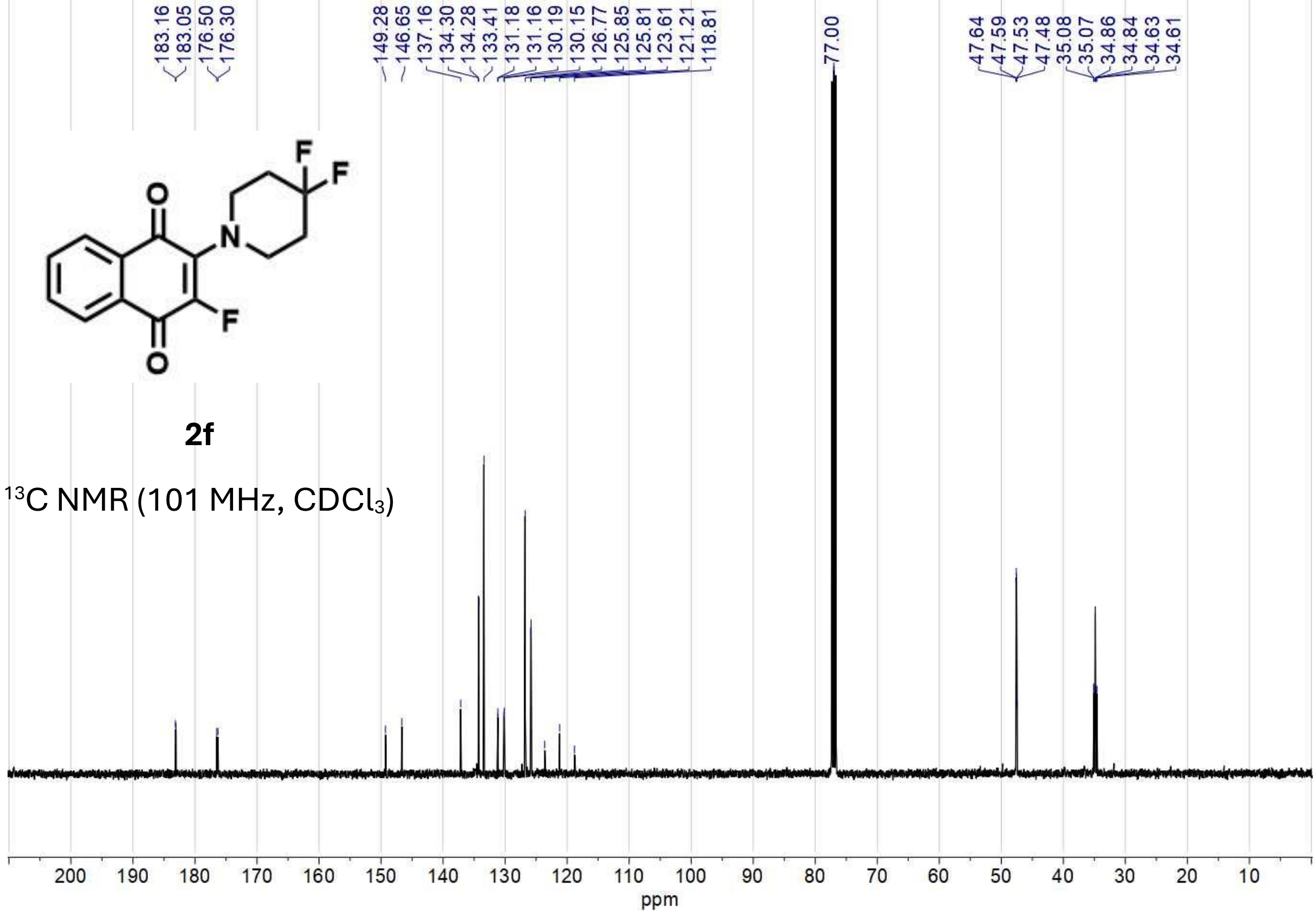
183.16 183.05 176.50 176.30
149.28 146.65 137.16 134.30 134.28 133.41 131.18 131.16 130.19 130.15 126.77 125.85 125.81 123.61 121.21 118.81
77.00
47.64 47.59 47.53 47.48 35.08 35.07 34.86 34.84 34.63 34.61
2f
^{13}C NMR (101 MHz, $CDCl_3$)
ppm

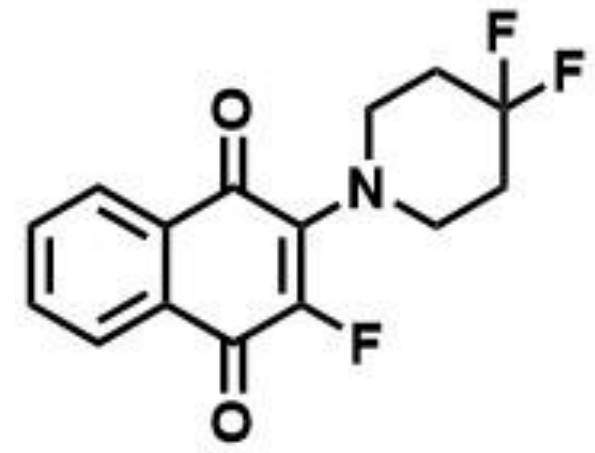

2f

^{19}F NMR (377 MHz, $CDCl_3$)

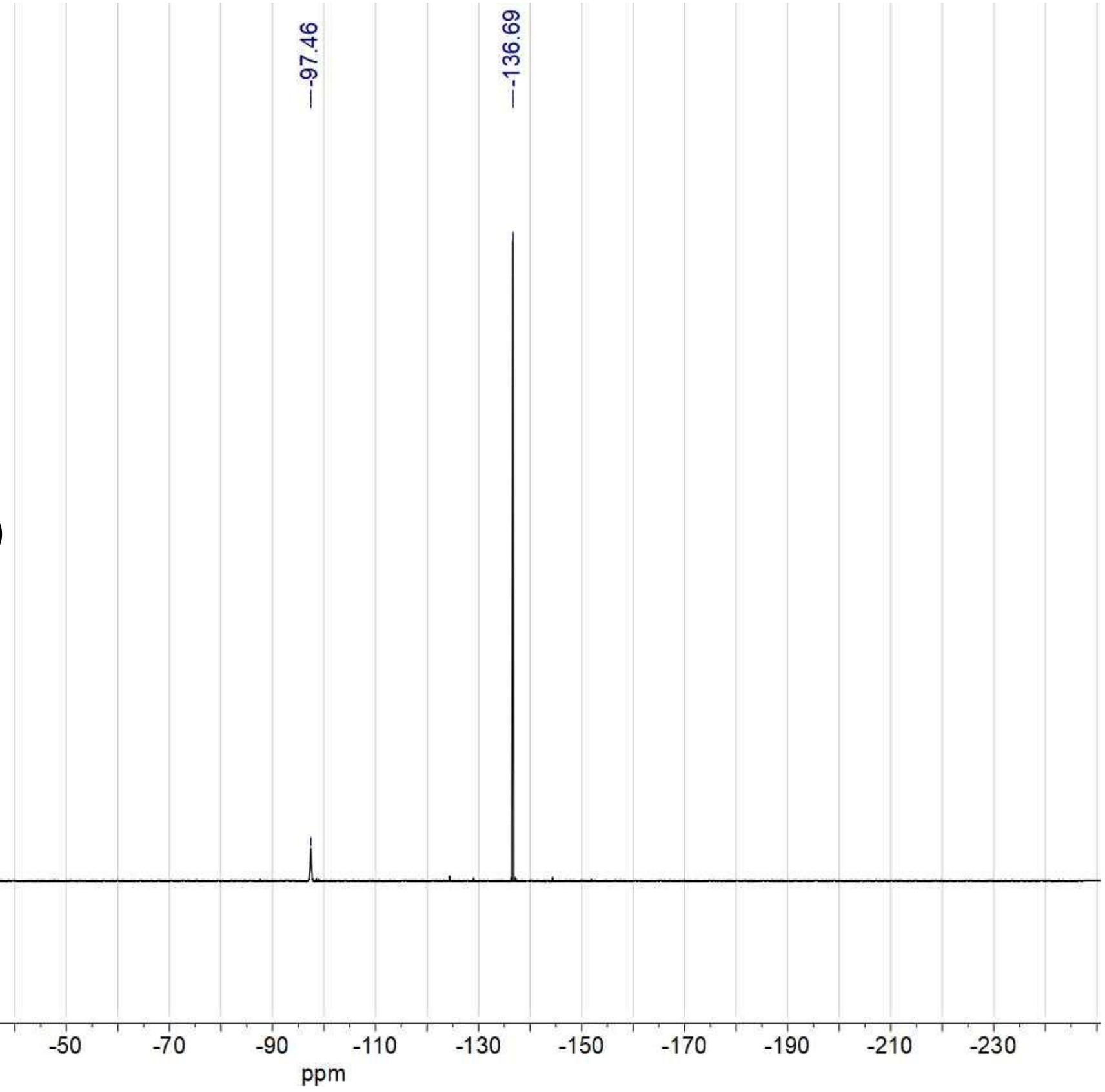

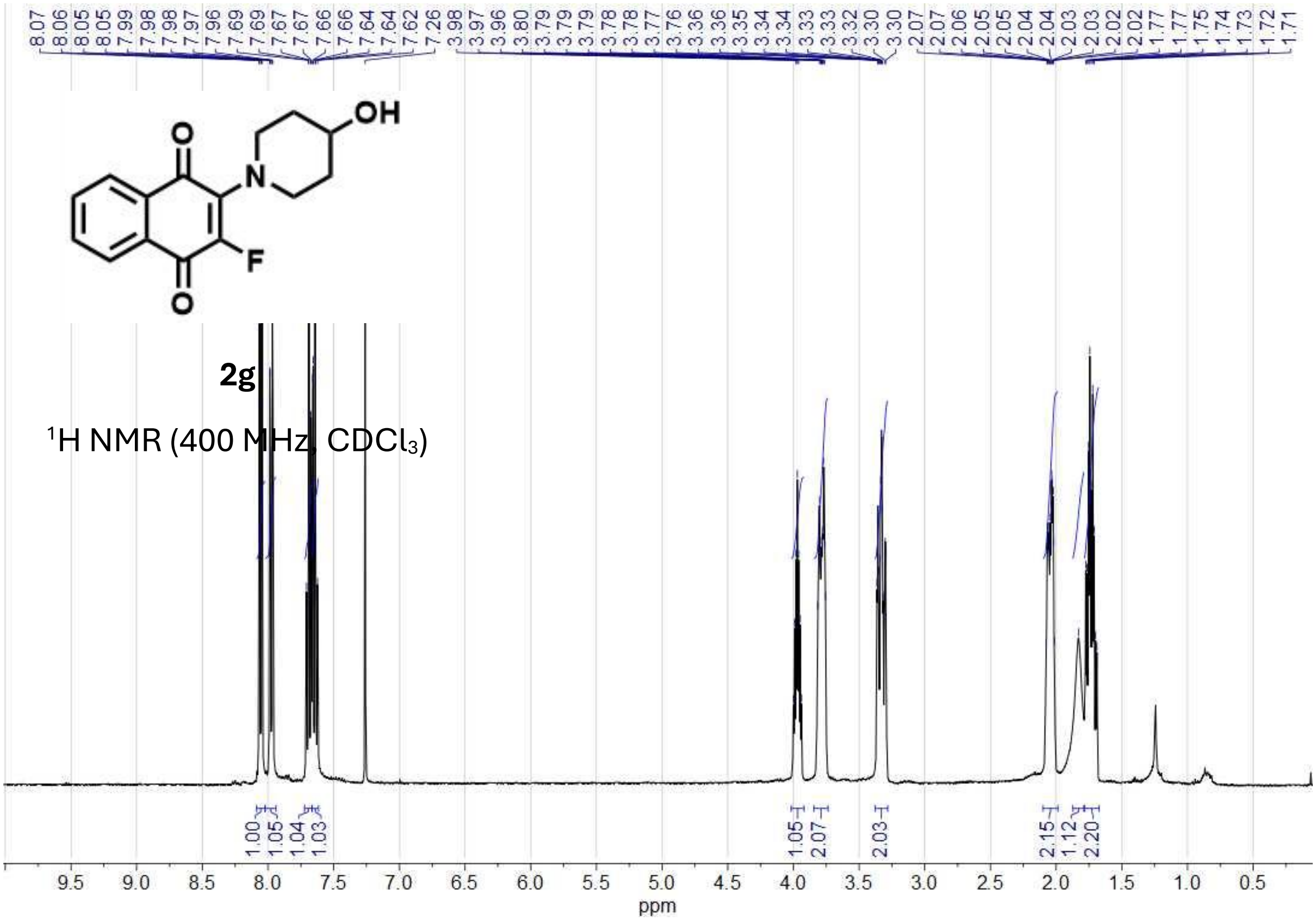

2g

^{1}H NMR (400 MHz, $CDCl_3$)

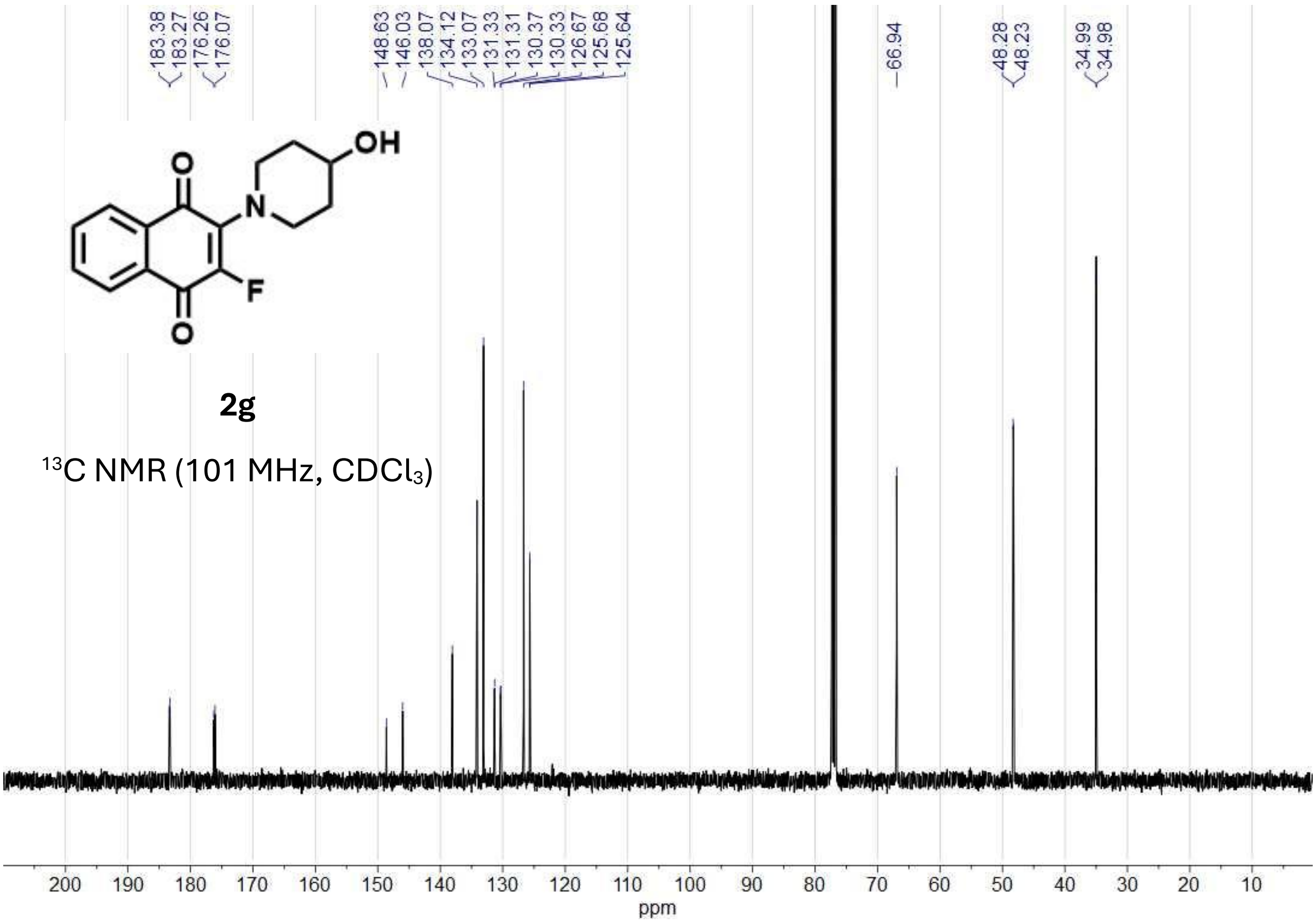

2g

^{13}C NMR (101 MHz, $CDCl_3$)

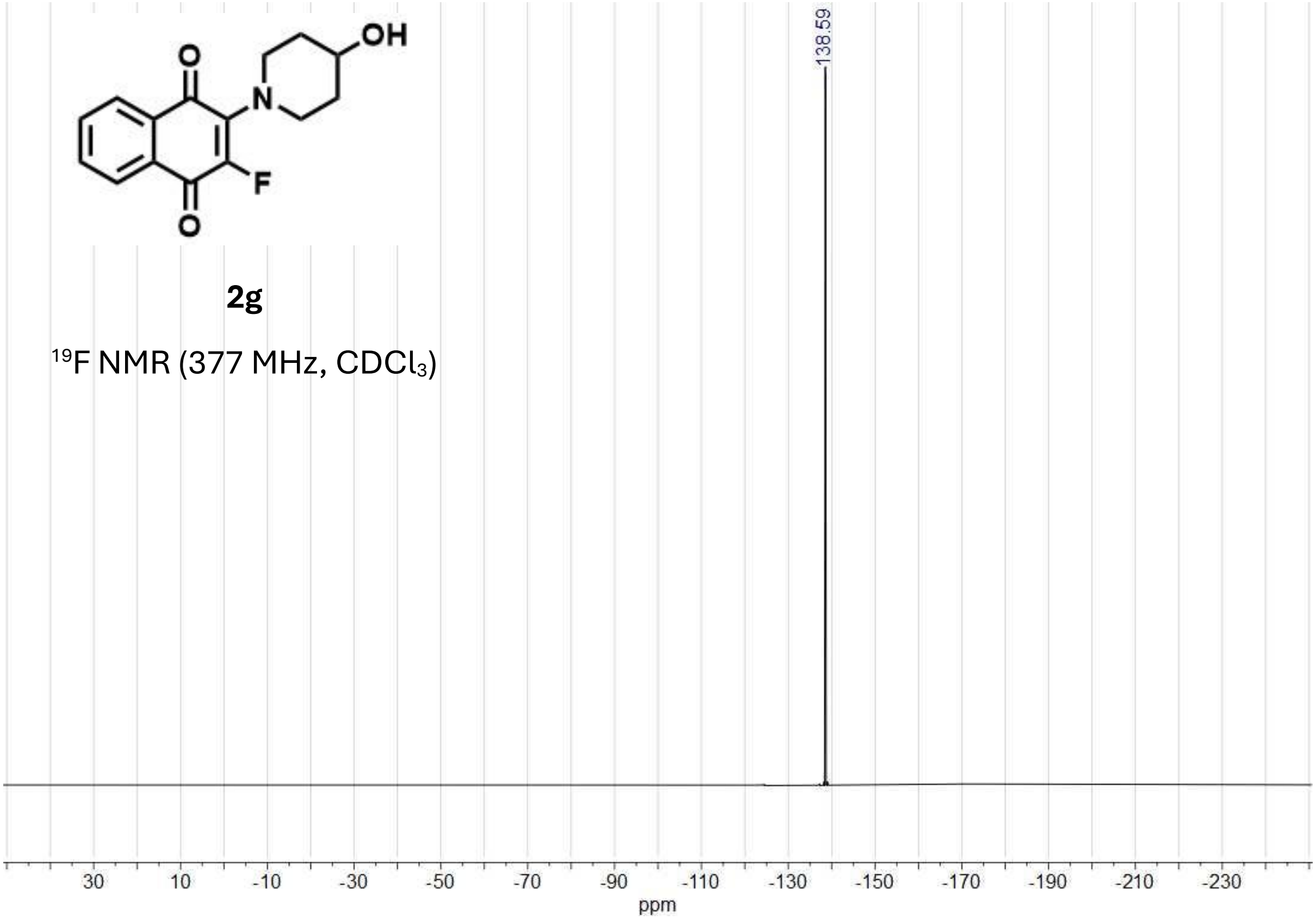

138.59
OH
O
N
F
O
2g
^{19}F NMR (377 MHz, $CDCl_3$)
30
10
-10
-30
-50
-70
-90
-110
-130
-150
-170
-190
-210
-230
ppm

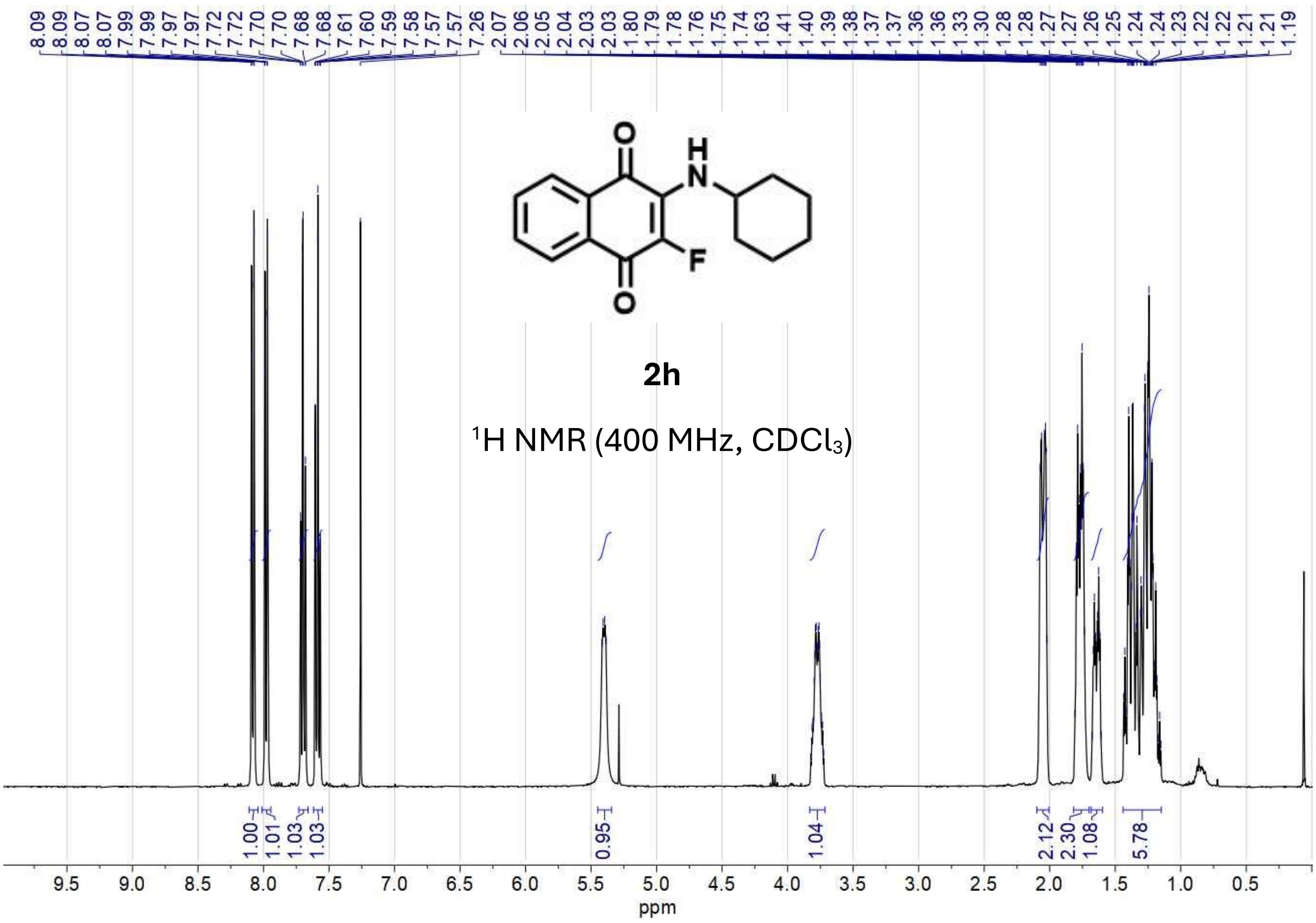

2h

^1H NMR (400 MHz, $CDCl_3$)

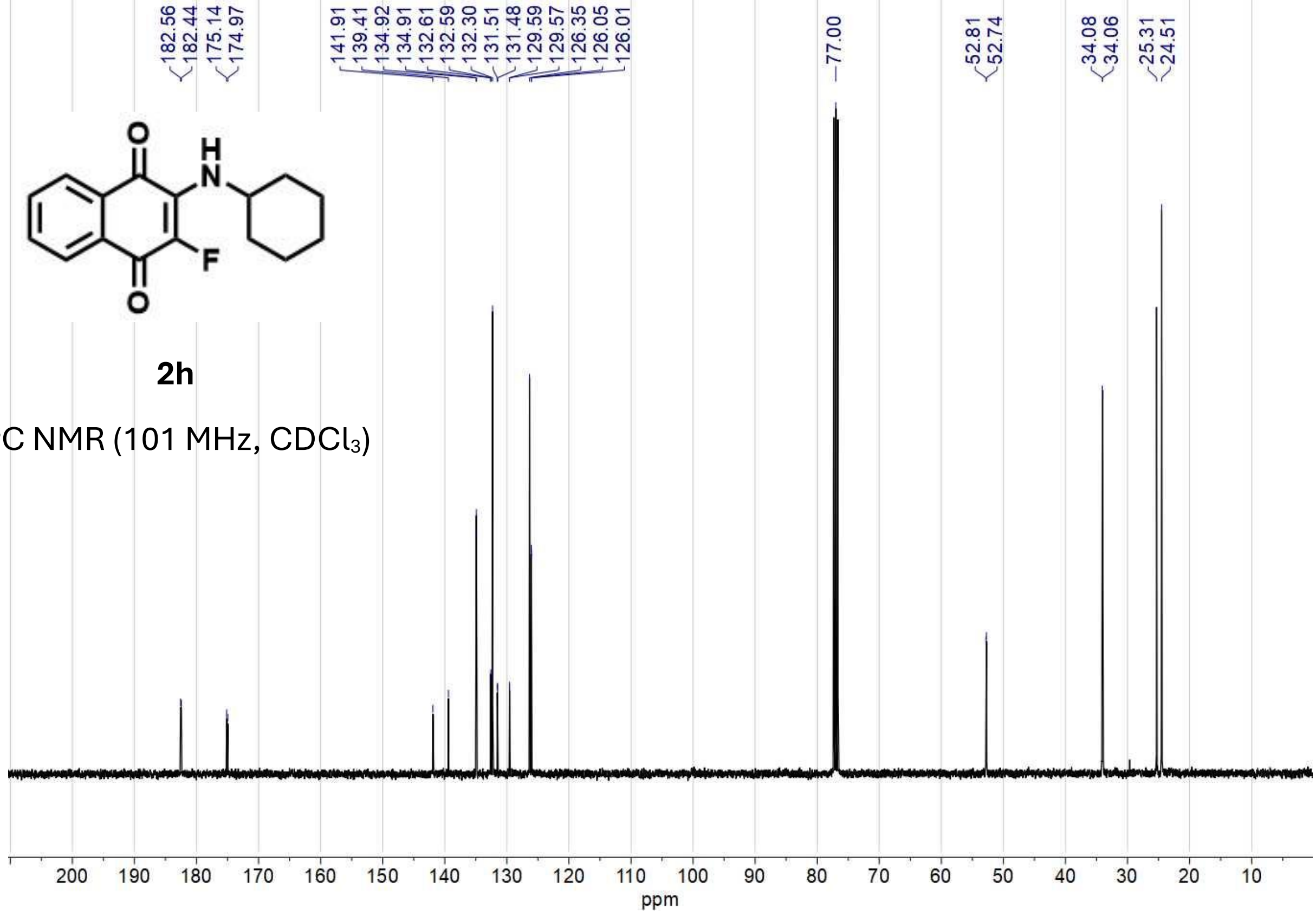

2h

^{13}C NMR (101 MHz, $CDCl_3$)

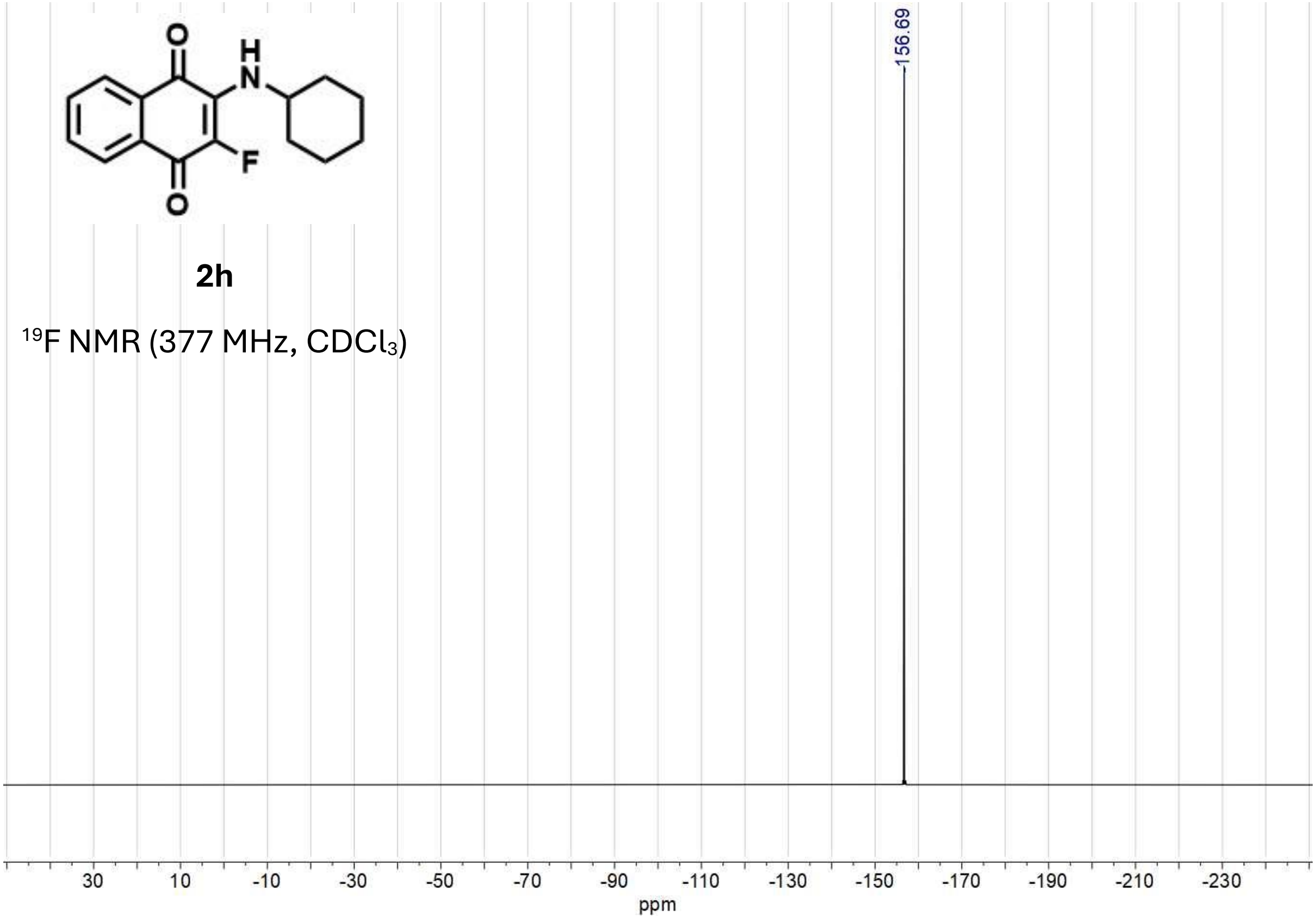

2h

^{19}F NMR (377 MHz, $CDCl_3$)

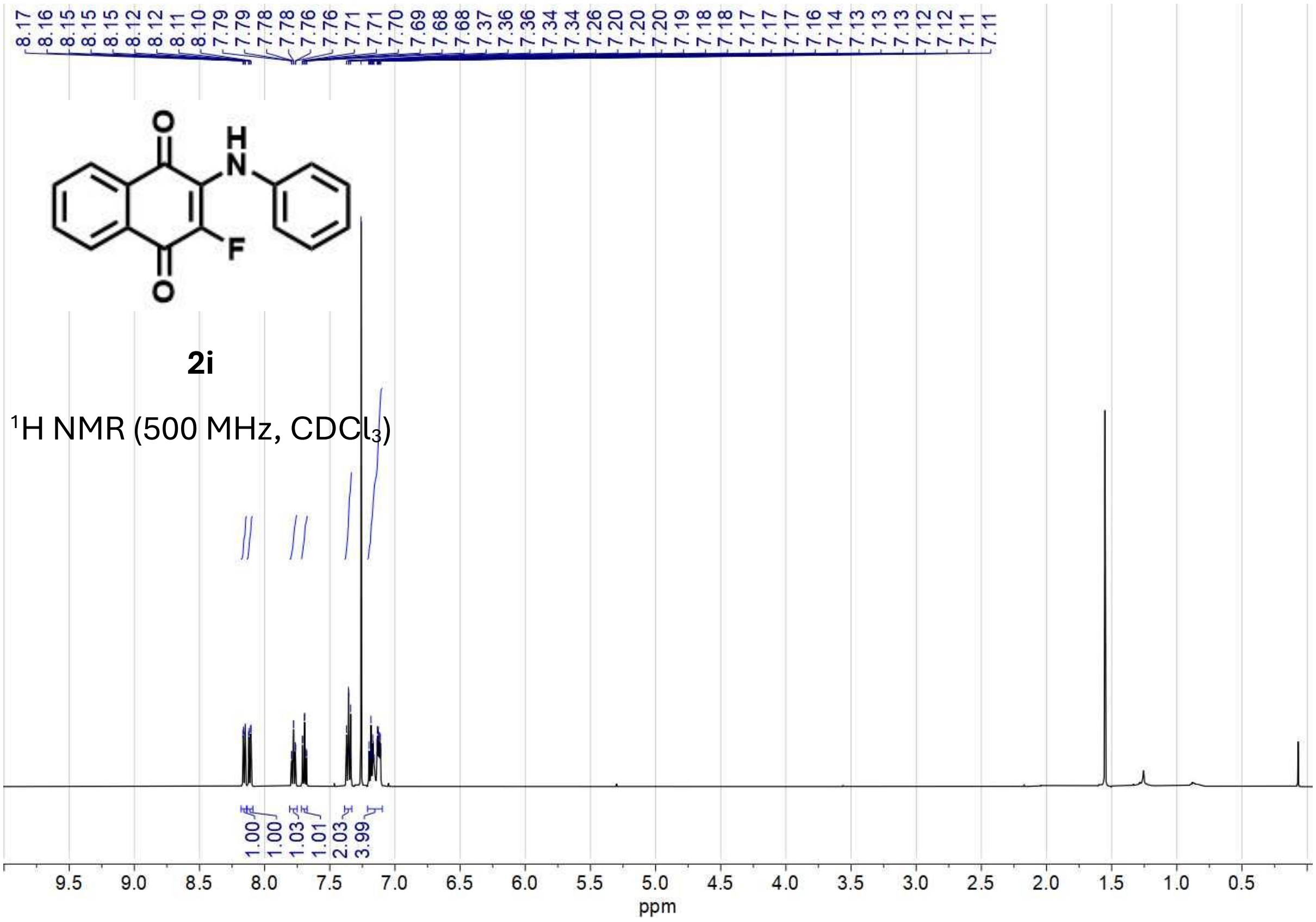

2i

^{1}H NMR (500 MHz, $CDCl_3$)

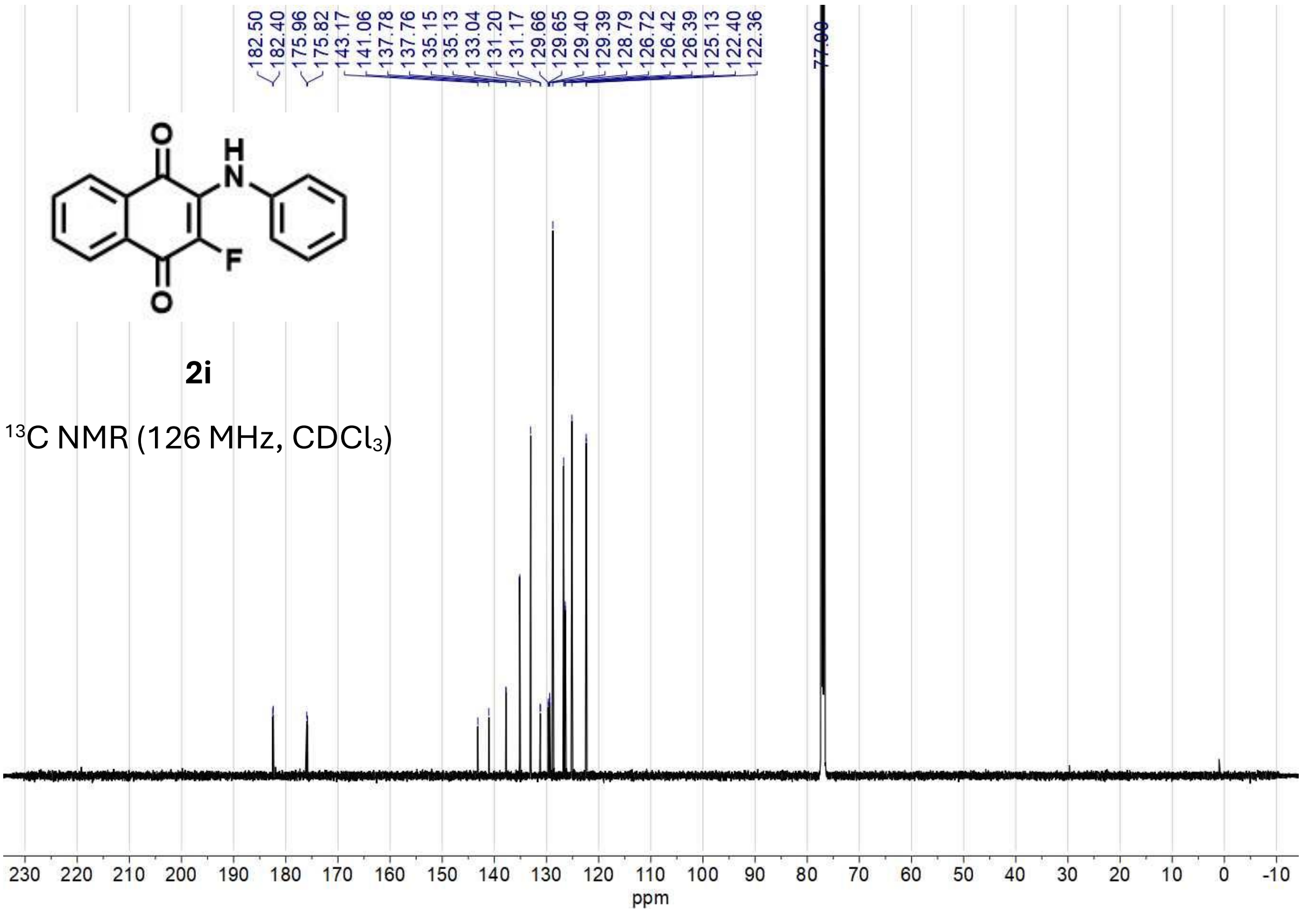

2i

^{13}C NMR (126 MHz, $CDCl_3$)

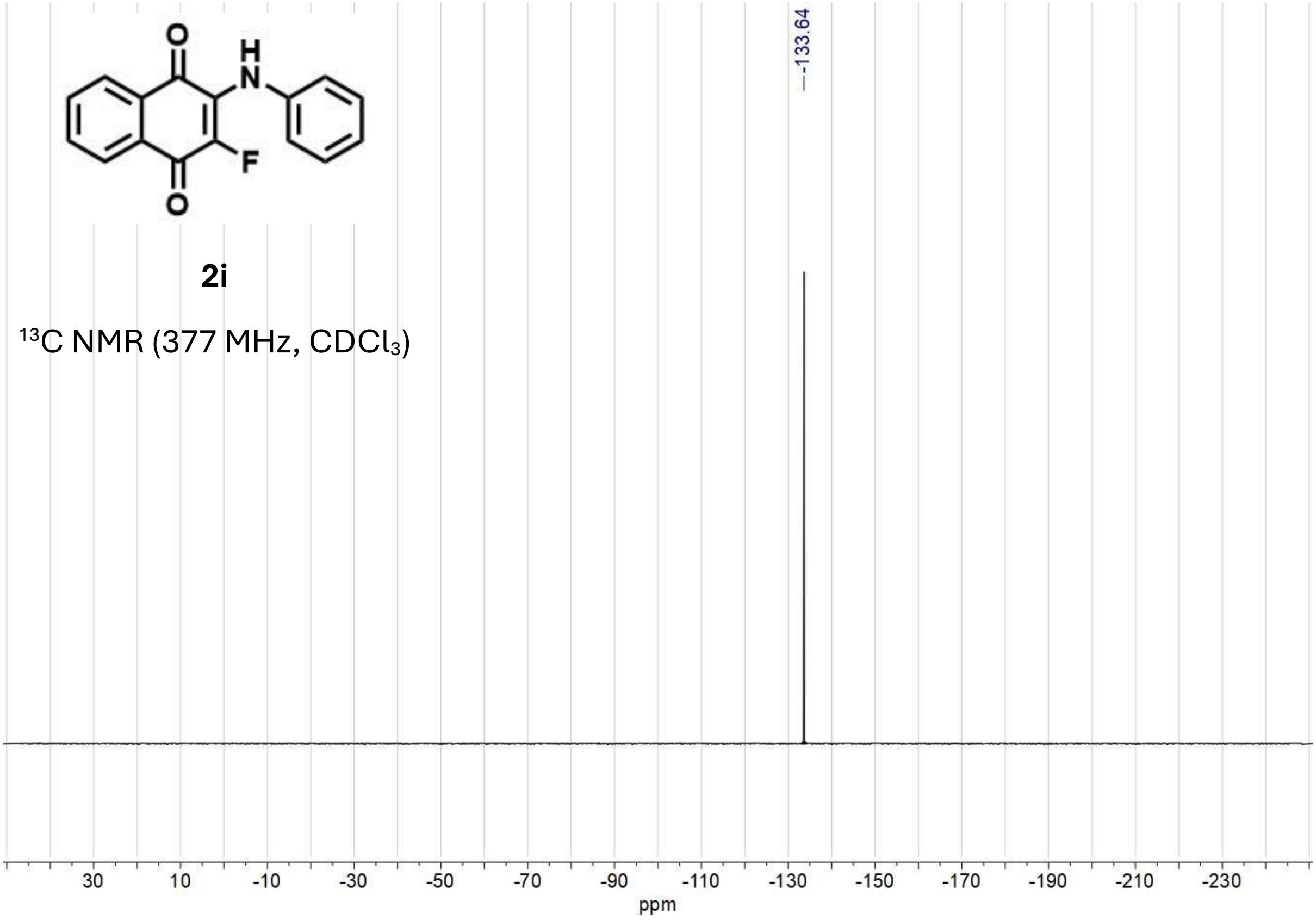
-133.64
2i
13C NMR (377 MHz, CDCl3)
30 10 -10 -30 -50 -70 -90 -110 -130 -150 -170 -190 -210 -230
ppm

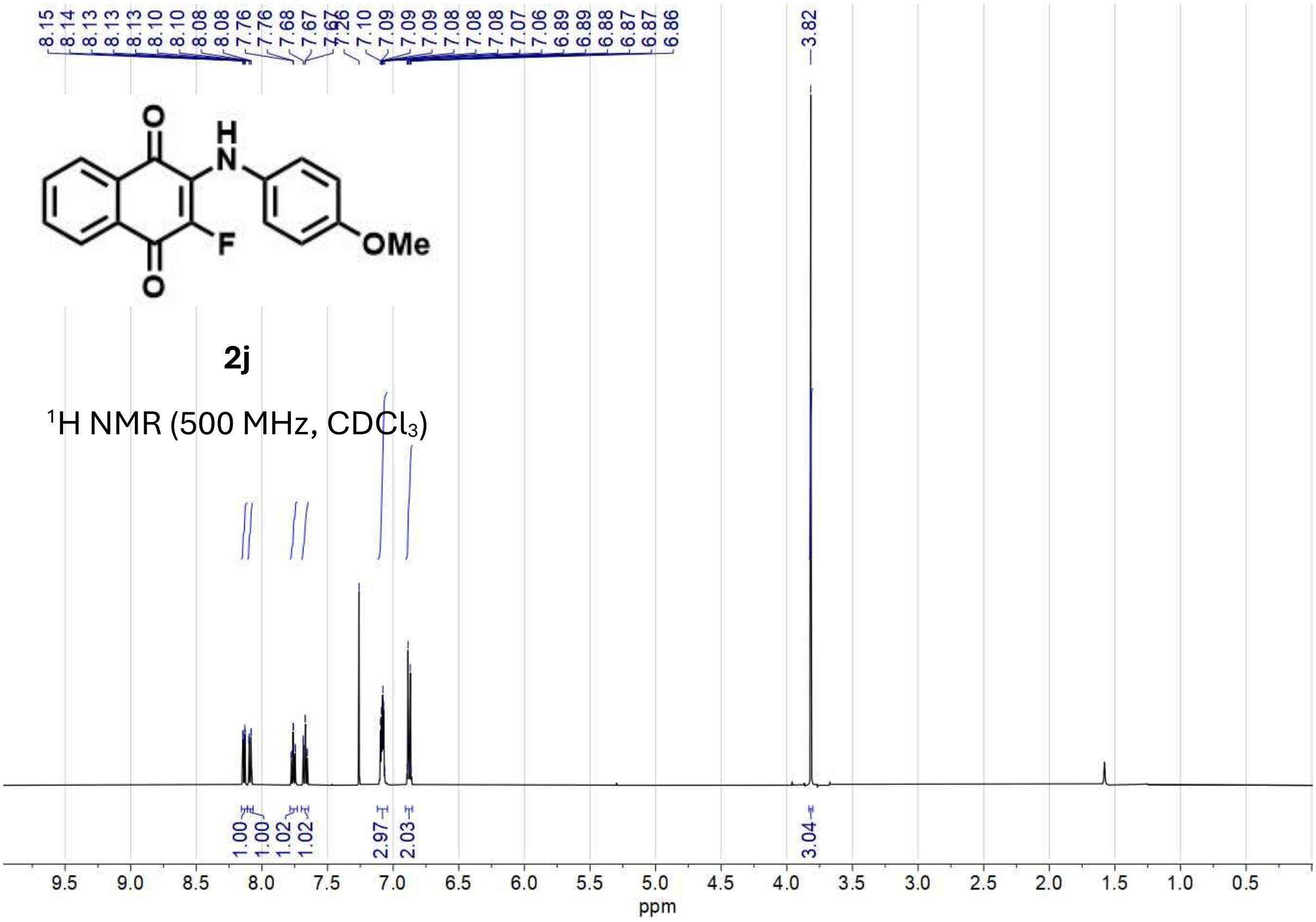
8.15 8.14 8.13 8.13 8.13 8.10 8.10 8.08 8.08 7.76 7.76 7.68 7.67 7.67 7.66 7.10 7.09 7.09 7.08 7.08 7.08 7.07 7.06 6.89 6.89 6.88 6.87 6.86
3.82
O
H
N
F
OMe
O
2j
1H NMR (500 MHz, CDCl3)
1.00 1.00 1.02 1.02 2.97 2.03 3.04
9.5 9.0 8.5 8.0 7.5 7.0 6.5 6.0 5.5 5.0 4.5 4.0 3.5 3.0 2.5 2.0 1.5 1.0 0.5
ppm

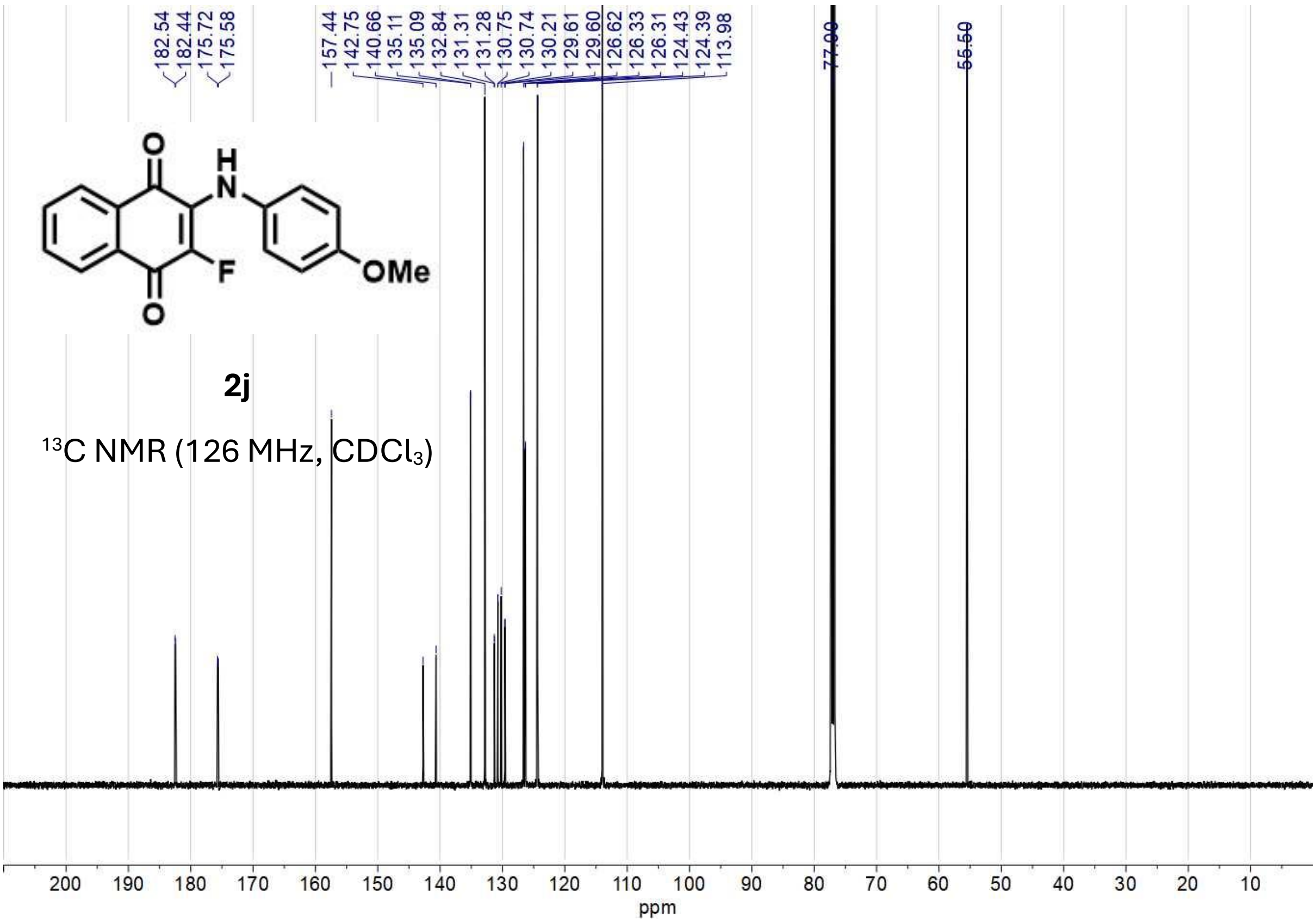
182.54 182.44 175.72 175.58
157.44 142.75 140.66 135.11 135.09 132.84 131.31 131.28 130.75 130.74 130.21 129.61 129.60 126.62 126.33 126.31 124.43 124.39 113.98
77.00
55.50
O
H
N
F
OMe
O
2j
13C NMR (126 MHz, CDCl3)
200 190 180 170 160 150 140 130 120 110 100 90 80 70 60 50 40 30 20 10
ppm

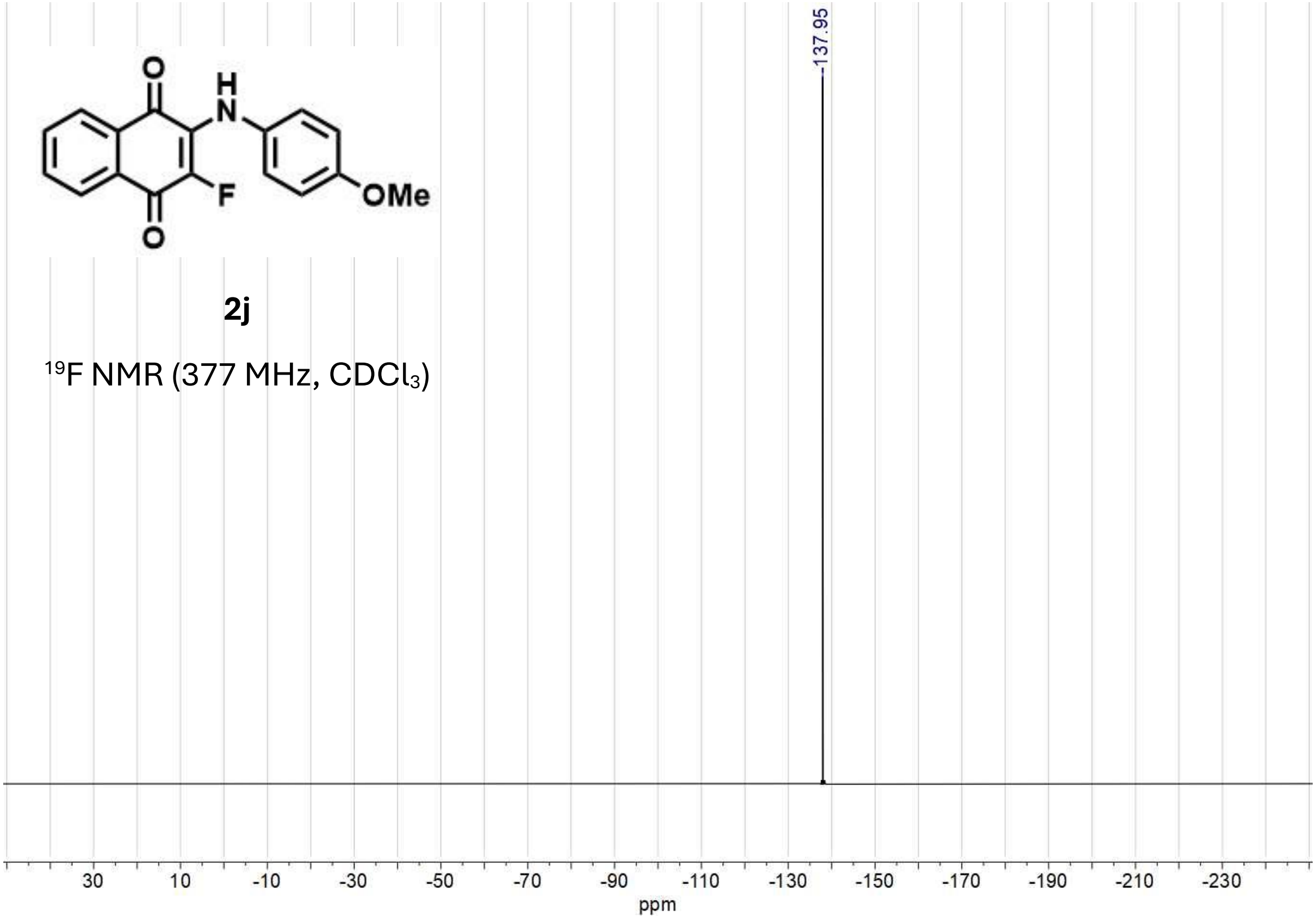

2j

^{19}F NMR (377 MHz, $CDCl_3$)

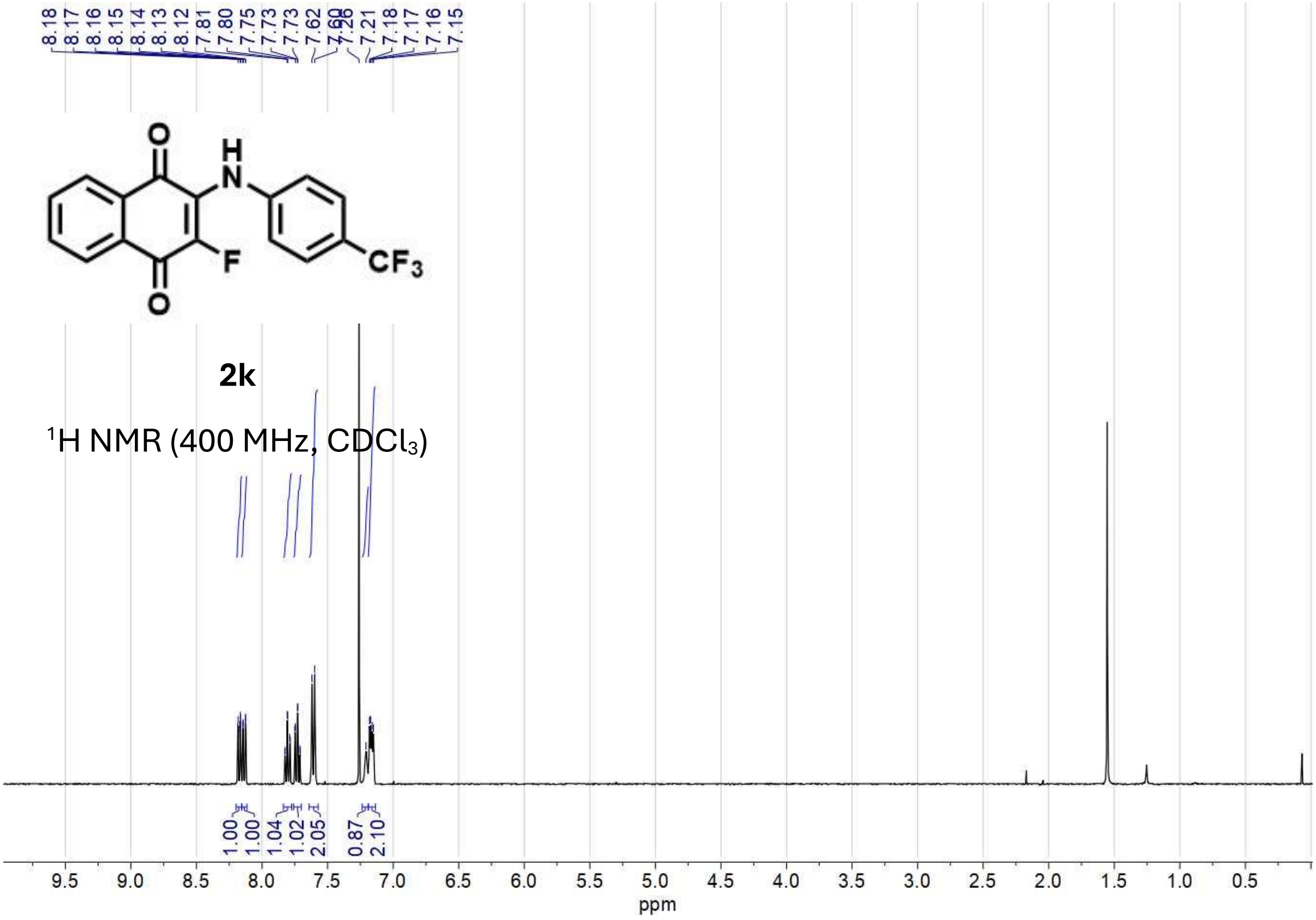
8.18
8.17
8.16
8.15
8.14
8.13
8.12
7.81
7.80
7.75
7.73
7.73
7.62
7.60
7.26
7.21
7.18
7.17
7.16
7.15
H
N
O
O
F
CF3
2k
1H NMR (400 MHz, CDCl3)
1.00
1.00
1.04
1.02
2.05
0.87
2.10
ppm

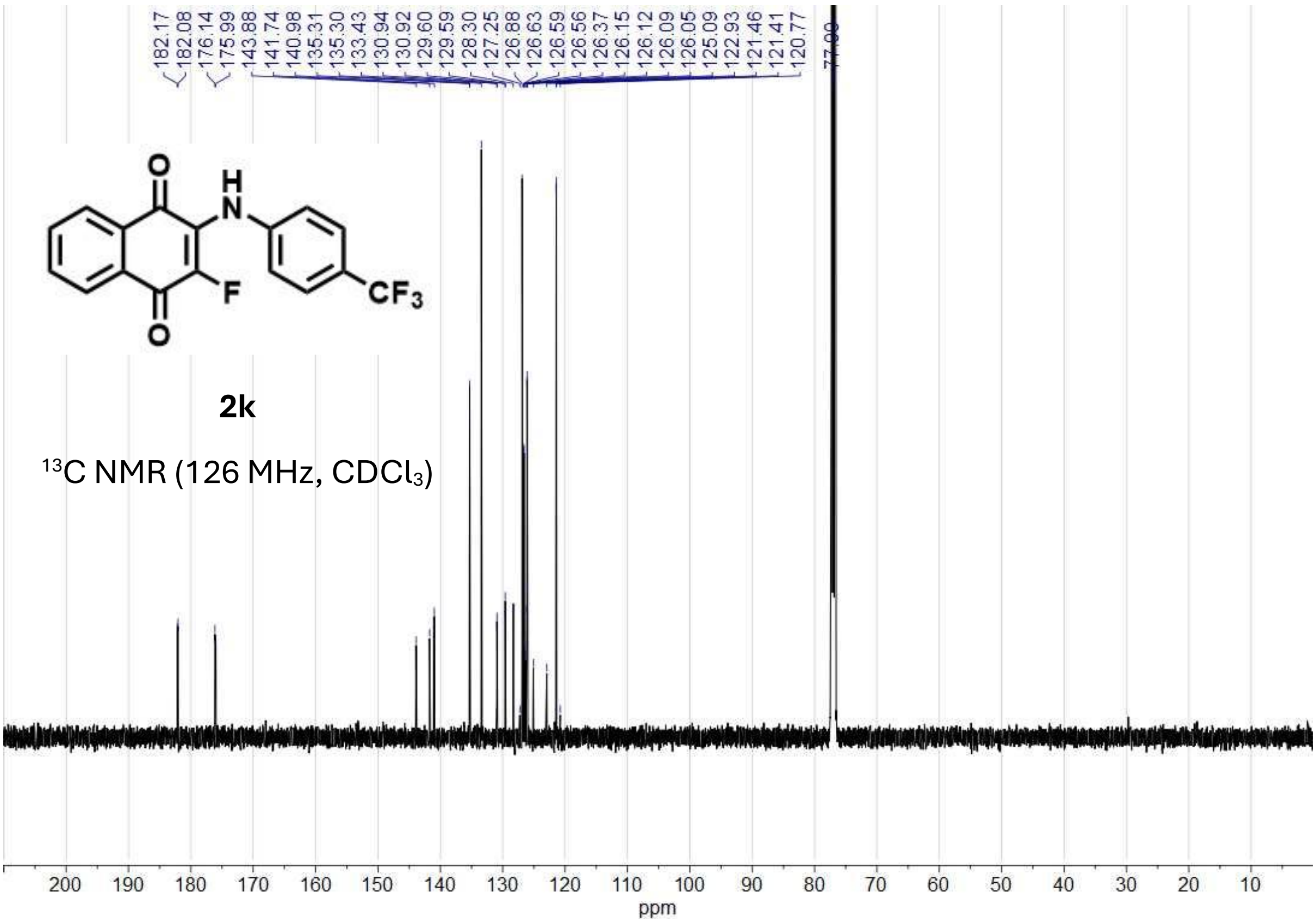
182.17
182.08
176.14
175.99
143.88
141.74
140.98
135.31
135.30
133.43
130.94
130.92
129.60
129.59
128.30
127.25
126.88
126.63
126.59
126.56
126.37
126.15
126.12
126.09
126.05
125.09
122.93
121.46
121.41
120.77
77.00
H
N
O
O
F
CF3
2k
13C NMR (126 MHz, CDCl3)
ppm

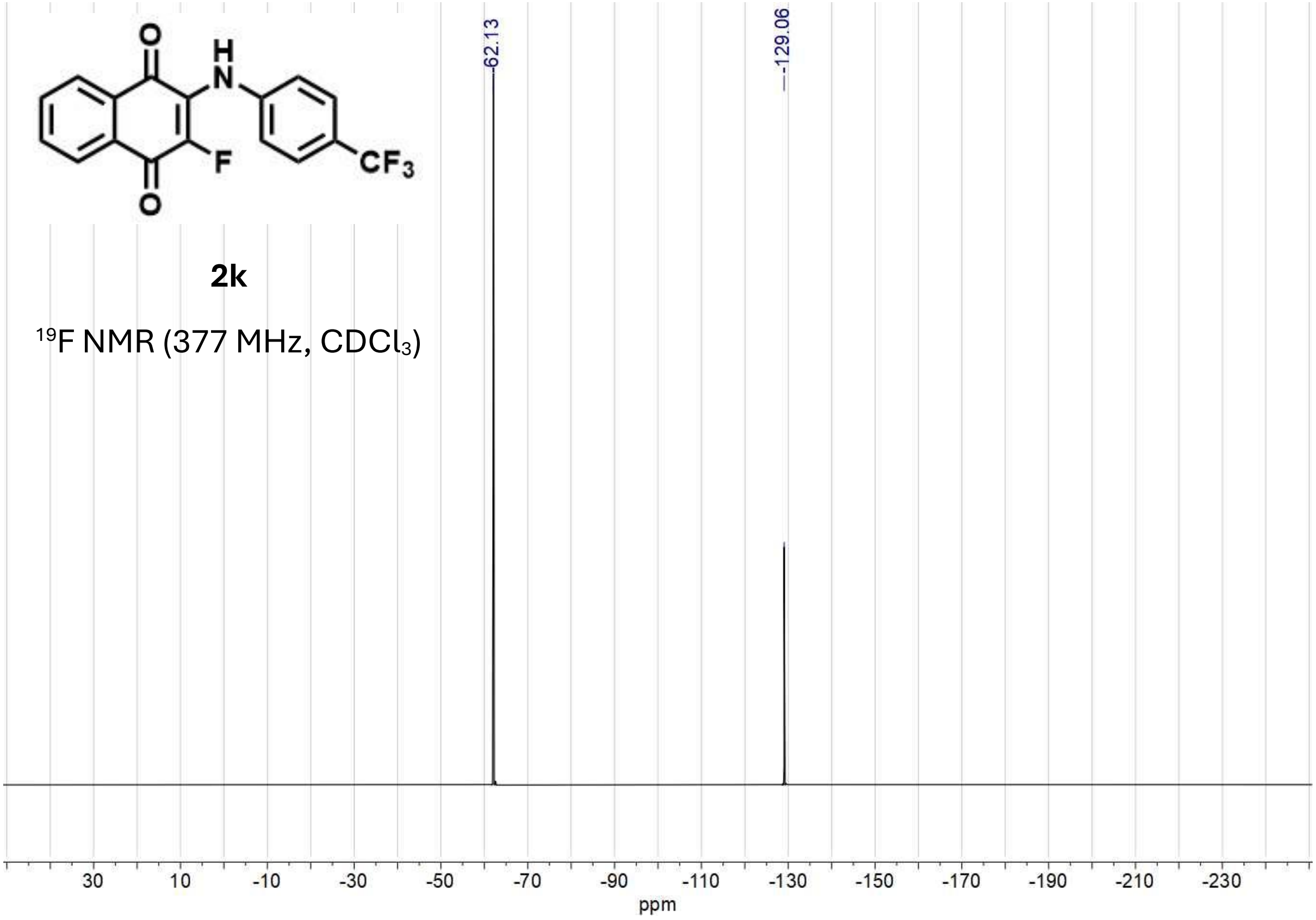

H
N
O
O
F
CF3
2k
19F NMR (377 MHz, CDCl3)
-62.13
-129.06
30
10
-10
-30
-50
-70
-90
-110
-130
-150
-170
-190
-210
-230
ppm

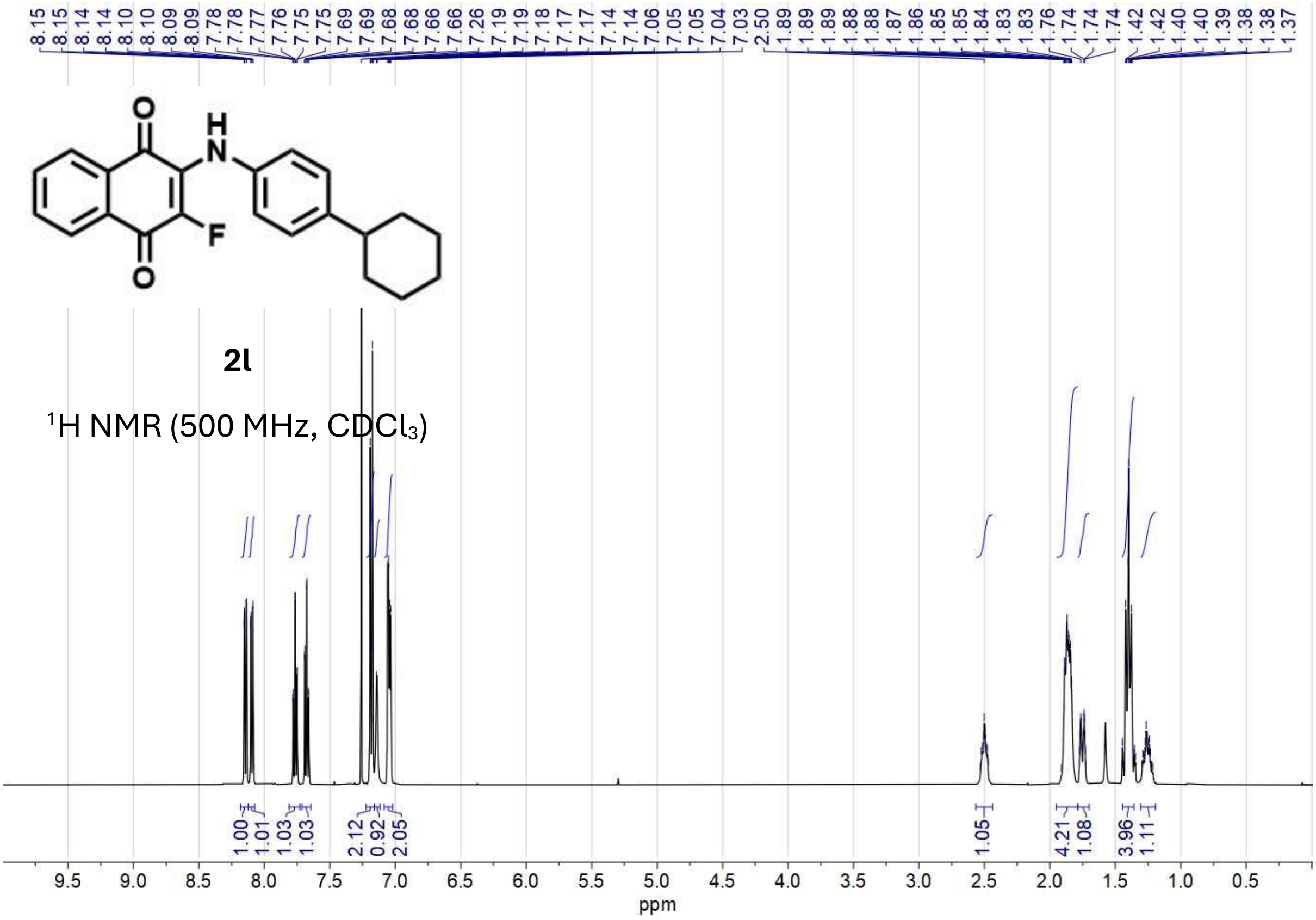
8.15 8.15 8.14 8.14 8.10 8.10 8.09 8.09 7.78 7.78 7.77 7.76 7.75 7.75 7.69 7.69 7.68 7.66 7.66 7.26 7.19 7.19 7.18 7.17 7.17 7.14 7.14 7.06 7.05 7.05 7.04 7.03 2.50 1.89 1.89 1.88 1.88 1.87 1.86 1.85 1.85 1.84 1.83 1.83 1.76 1.74 1.74 1.74 1.42 1.42 1.40 1.40 1.39 1.38 1.38 1.37
O
H
N
F
O
2l
1H NMR (500 MHz, CDCl3)
1.00 1.01 1.03 1.03 2.12 0.92 2.05 1.05 4.21 1.08 3.96 1.11
9.5 9.0 8.5 8.0 7.5 7.0 6.5 6.0 5.5 5.0 4.5 4.0 3.5 3.0 2.5 2.0 1.5 1.0 0.5
ppm

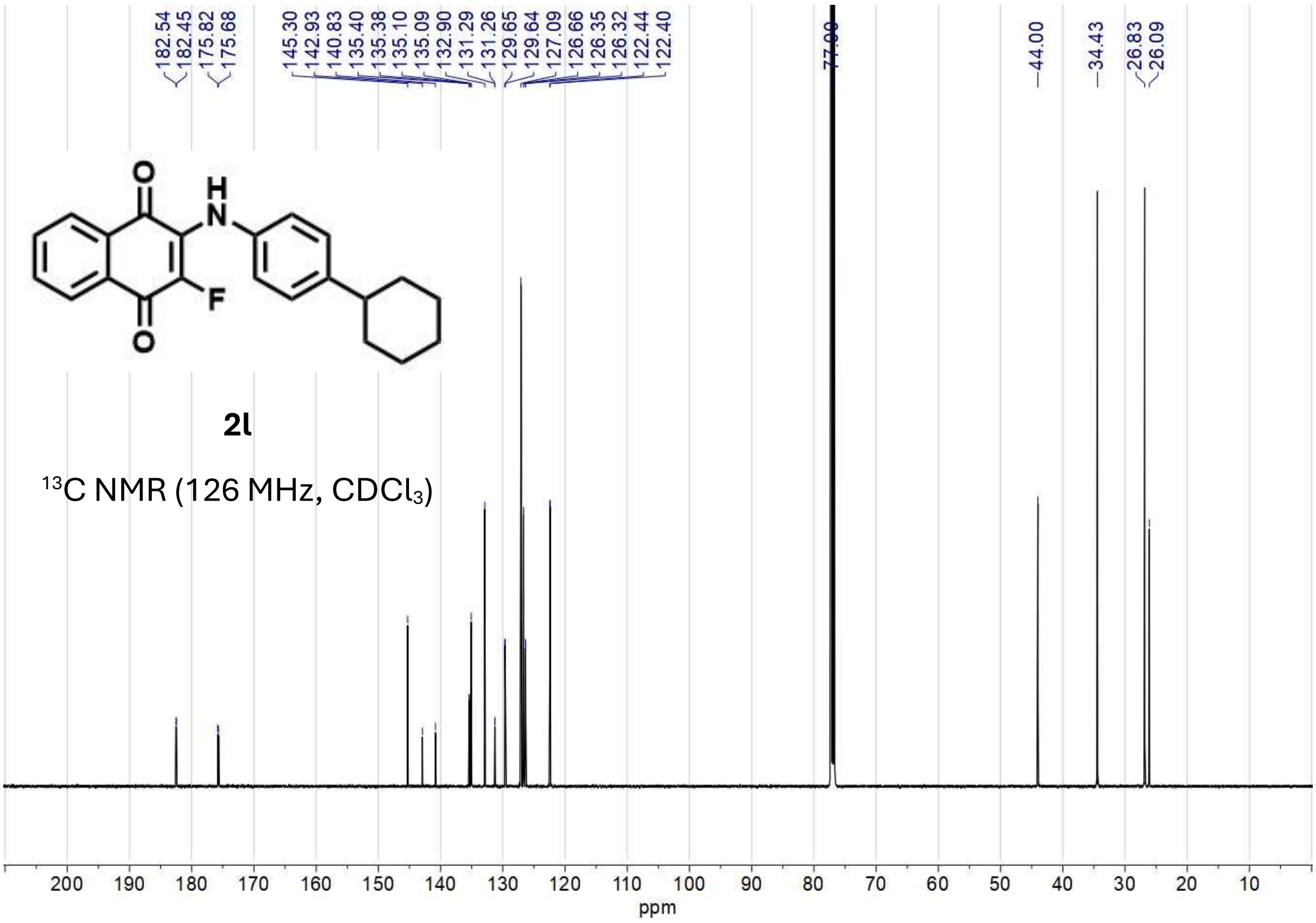
182.54 182.45 175.82 175.68 145.30 142.93 140.83 135.40 135.38 135.10 135.09 132.90 131.29 131.26 129.65 129.64 127.09 126.66 126.35 126.32 122.44 122.40 77.00 44.00 34.43 26.83 26.09
O
H
N
F
O
2l
13C NMR (126 MHz, CDCl3)
200 190 180 170 160 150 140 130 120 110 100 90 80 70 60 50 40 30 20 10
ppm

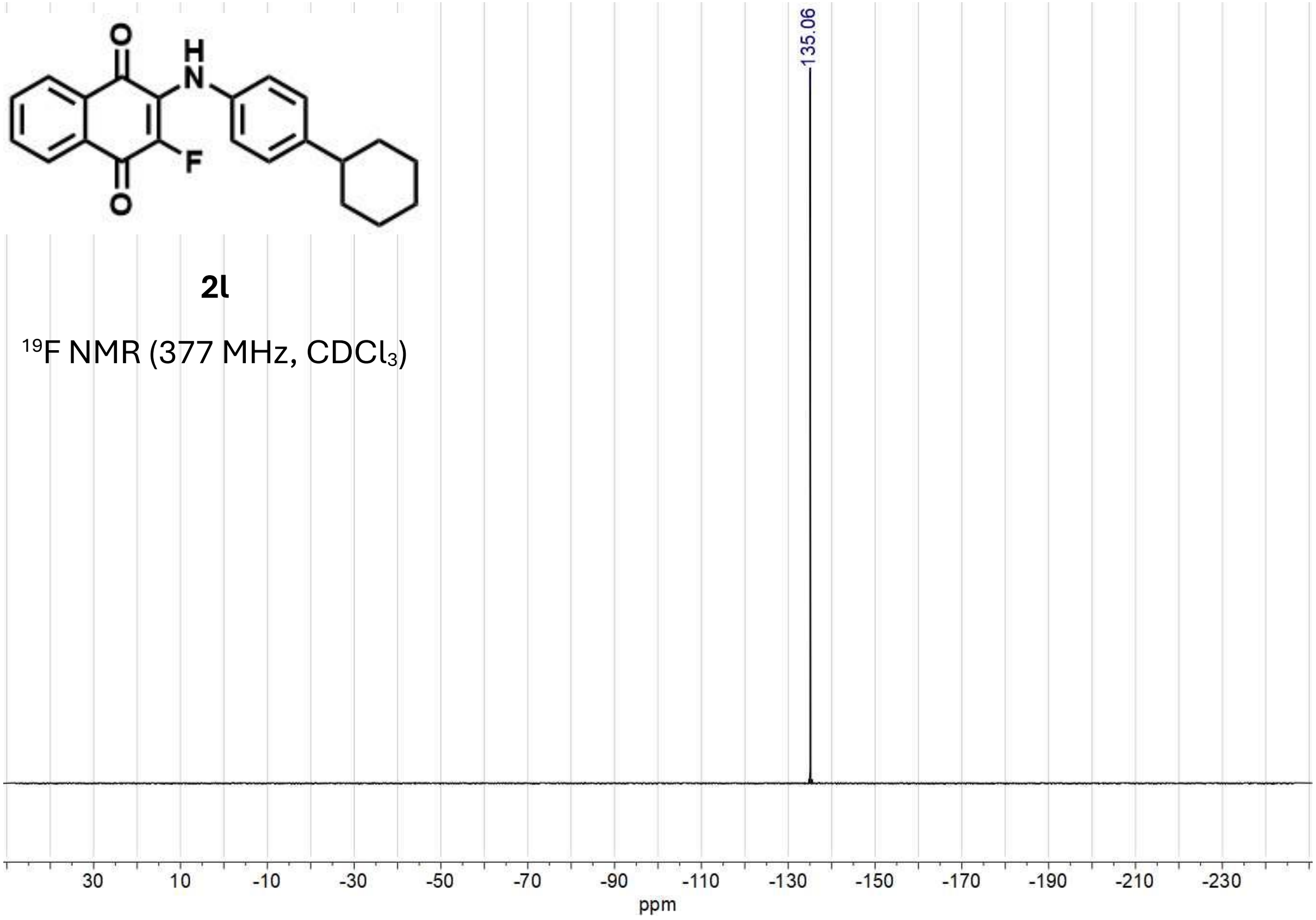

2l

^{19}F NMR (377 MHz, $CDCl_3$)

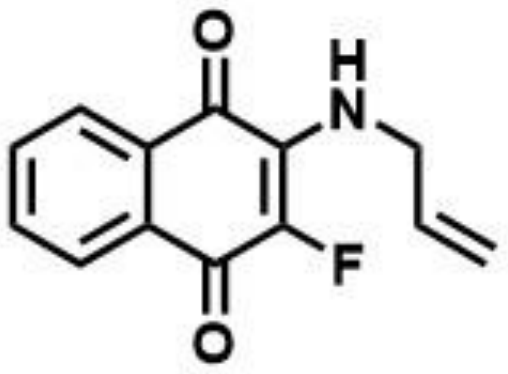

2m

^1H NMR (400 MHz, $CDCl_3$)

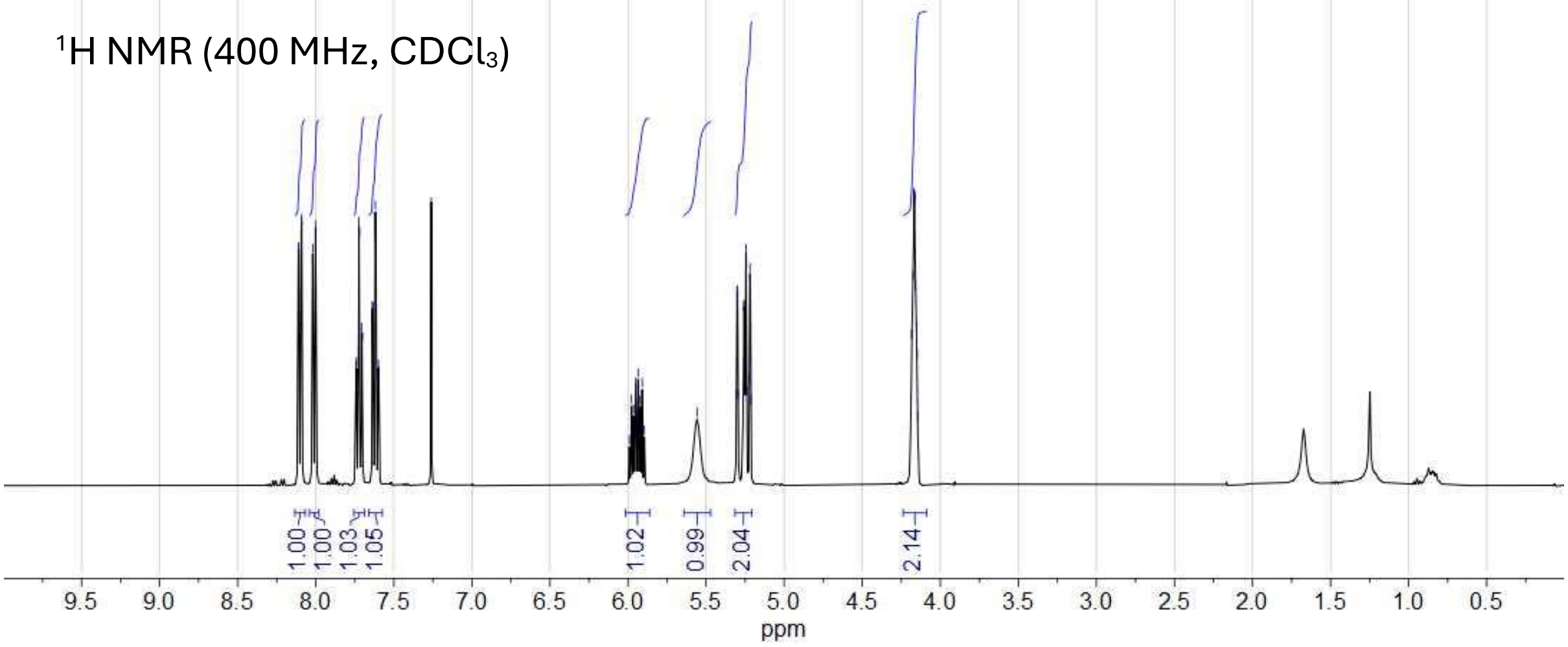

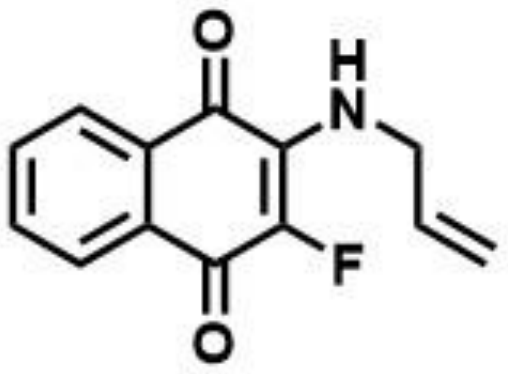

2m

^{13}C NMR (101 MHz, $CDCl_3$)

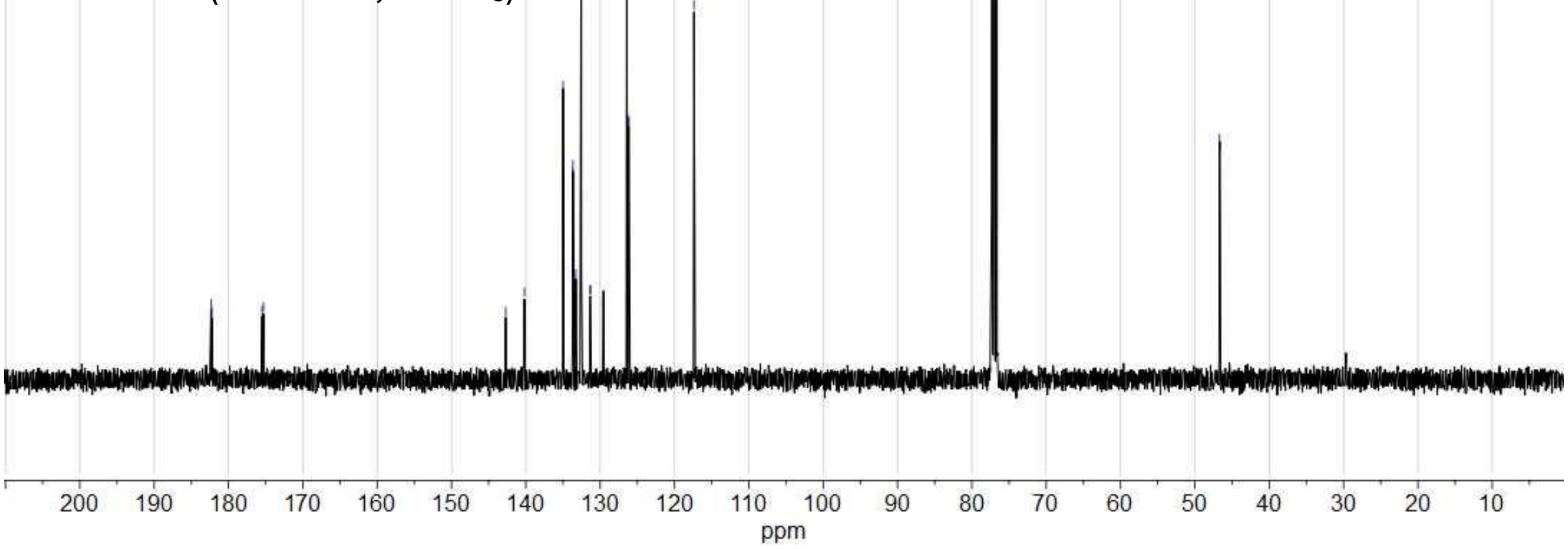

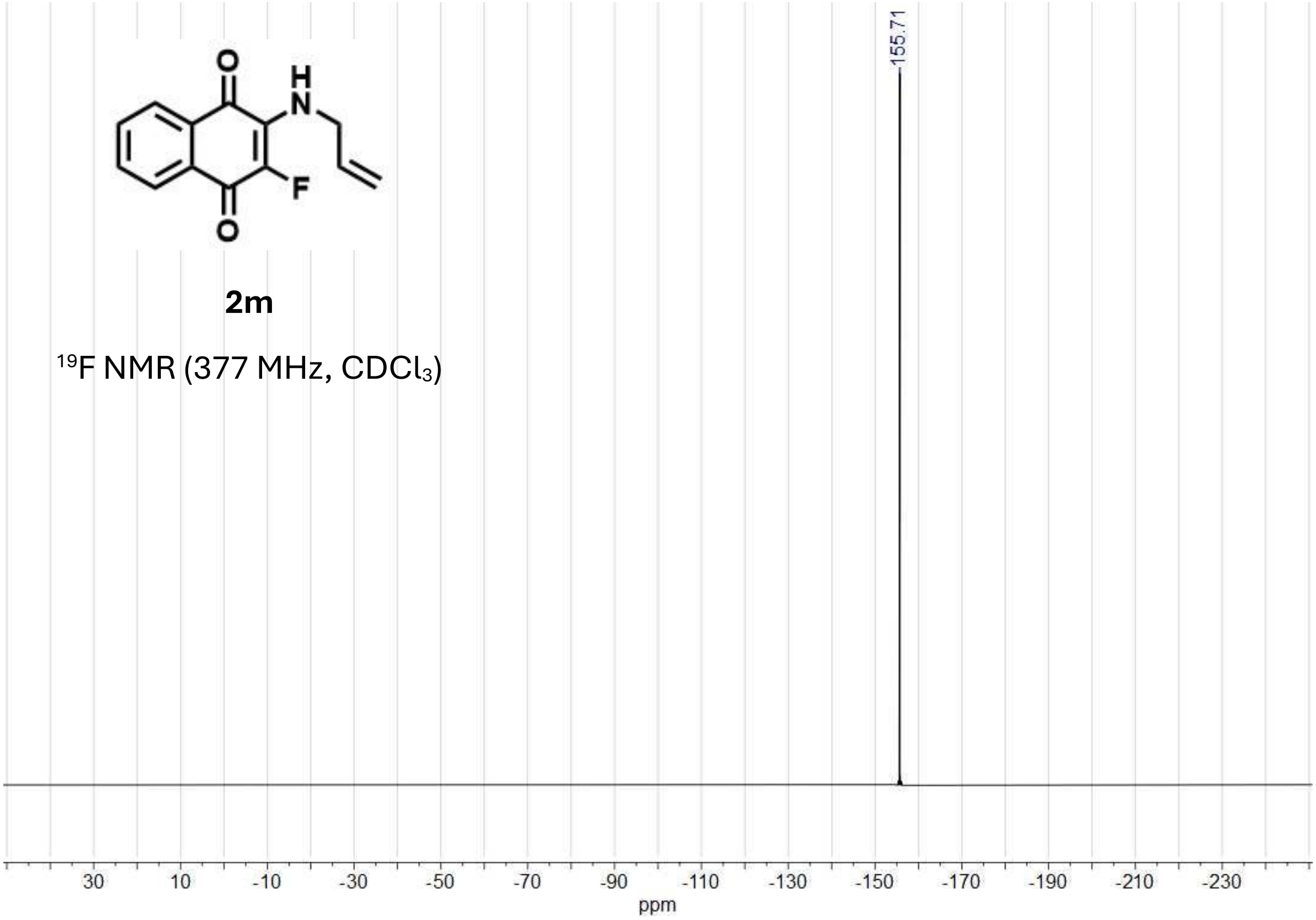

2m

^{19}F NMR (377 MHz, $CDCl_3$)

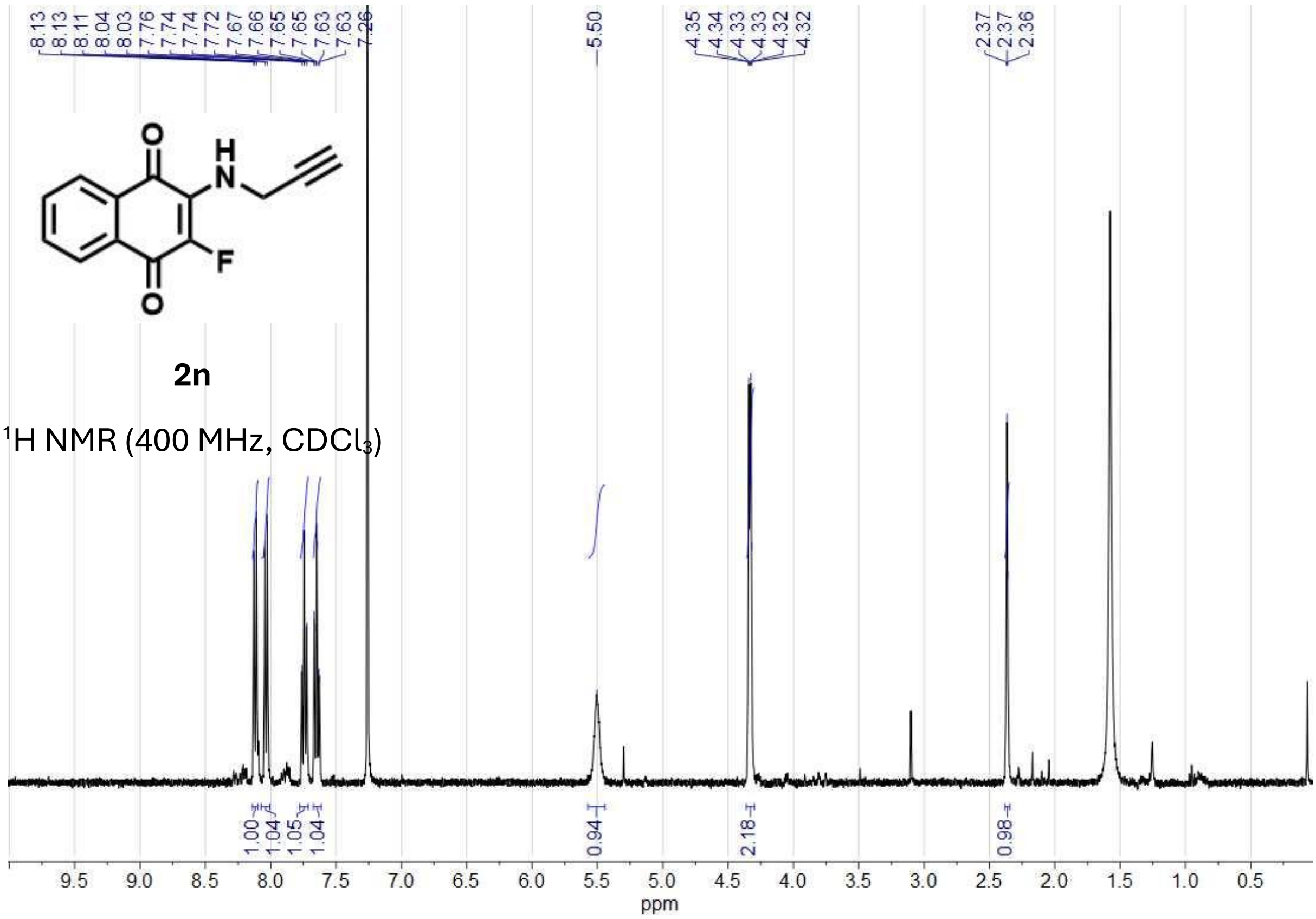
2n
^{1}H NMR (400 MHz, $CDCl_3$)
ppm

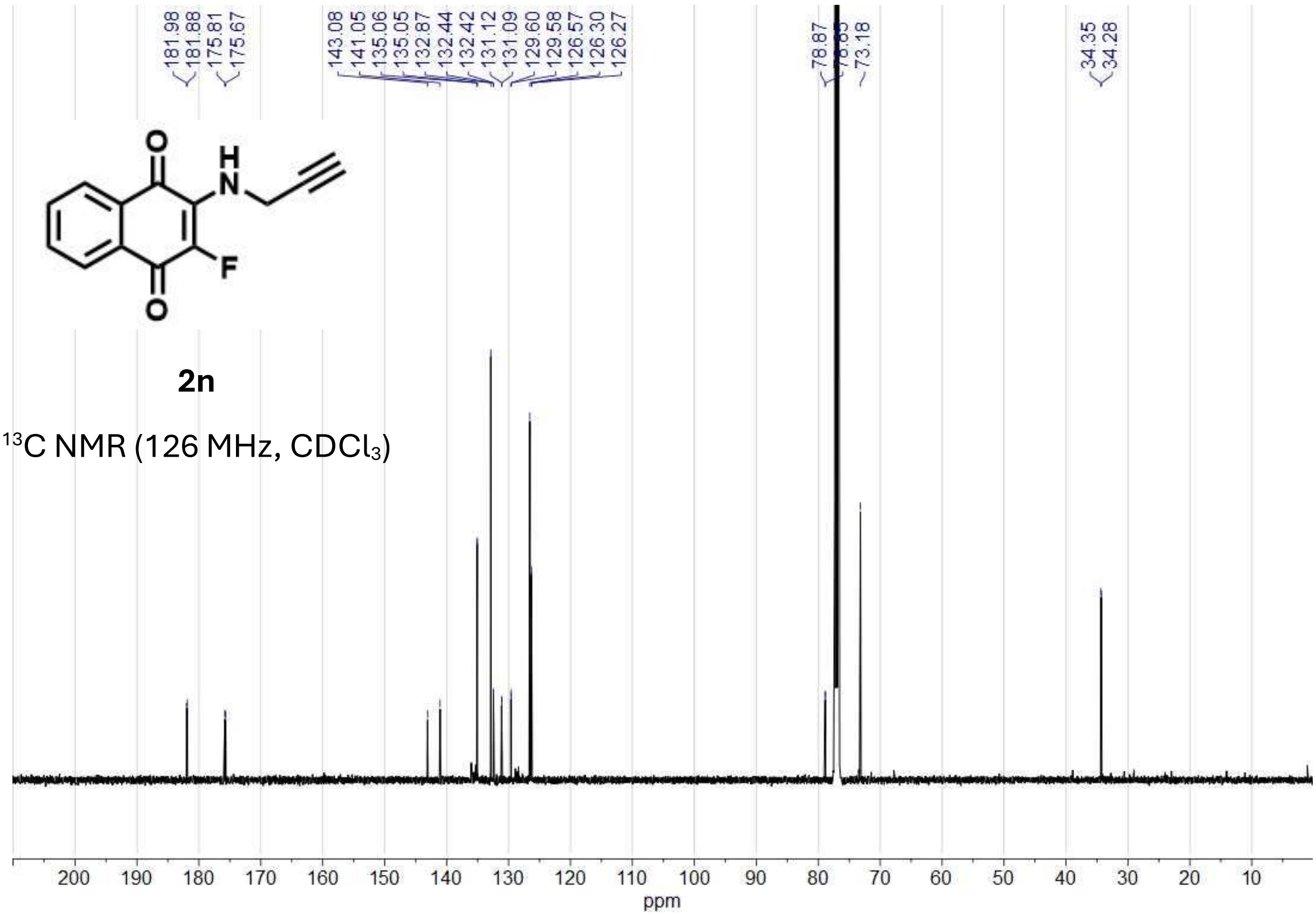
2n
^{13}C NMR (126 MHz, $CDCl_3$)
ppm

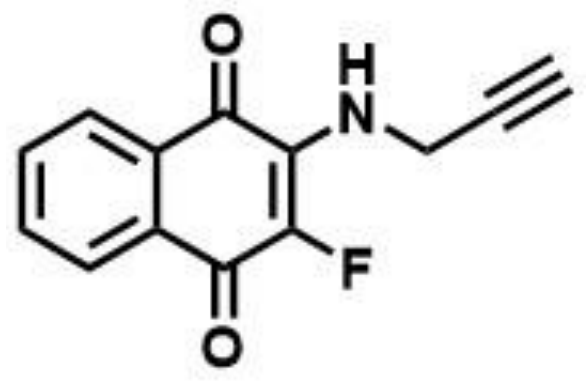

2n

^{19}F NMR (377 MHz, $CDCl_3$)

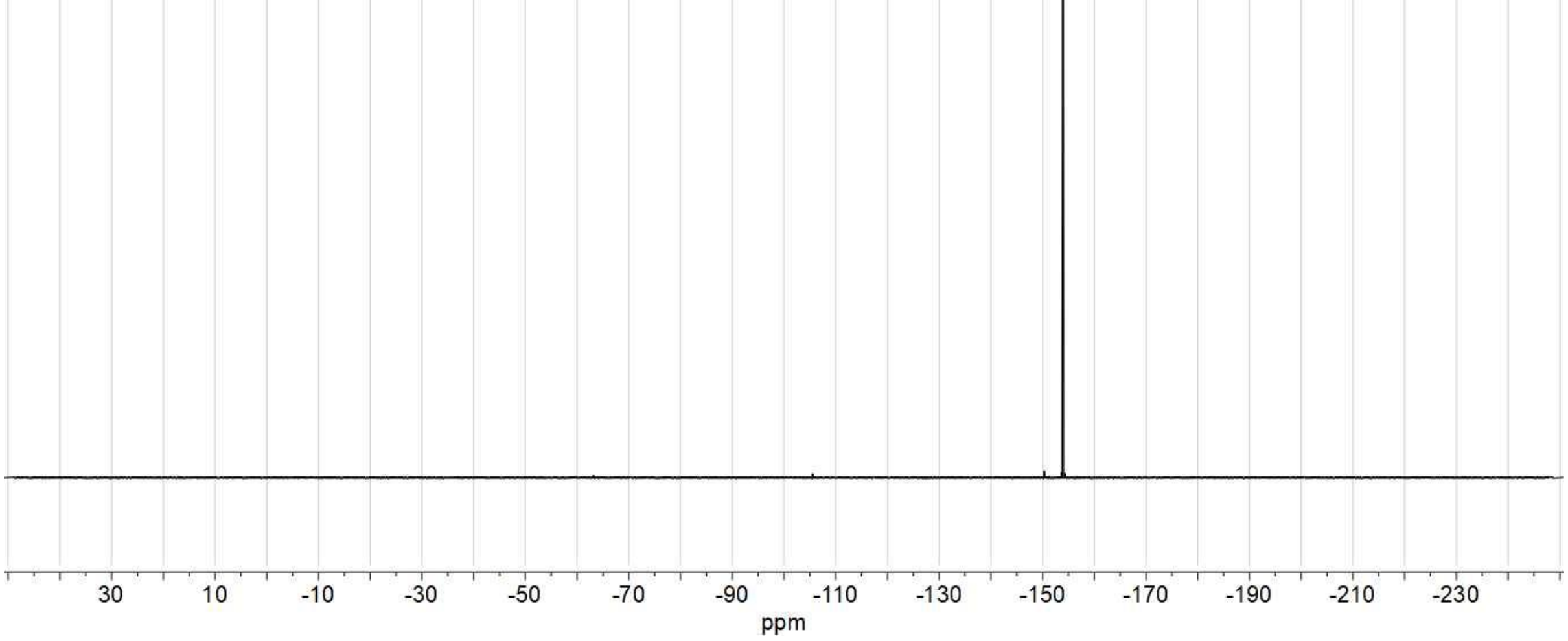

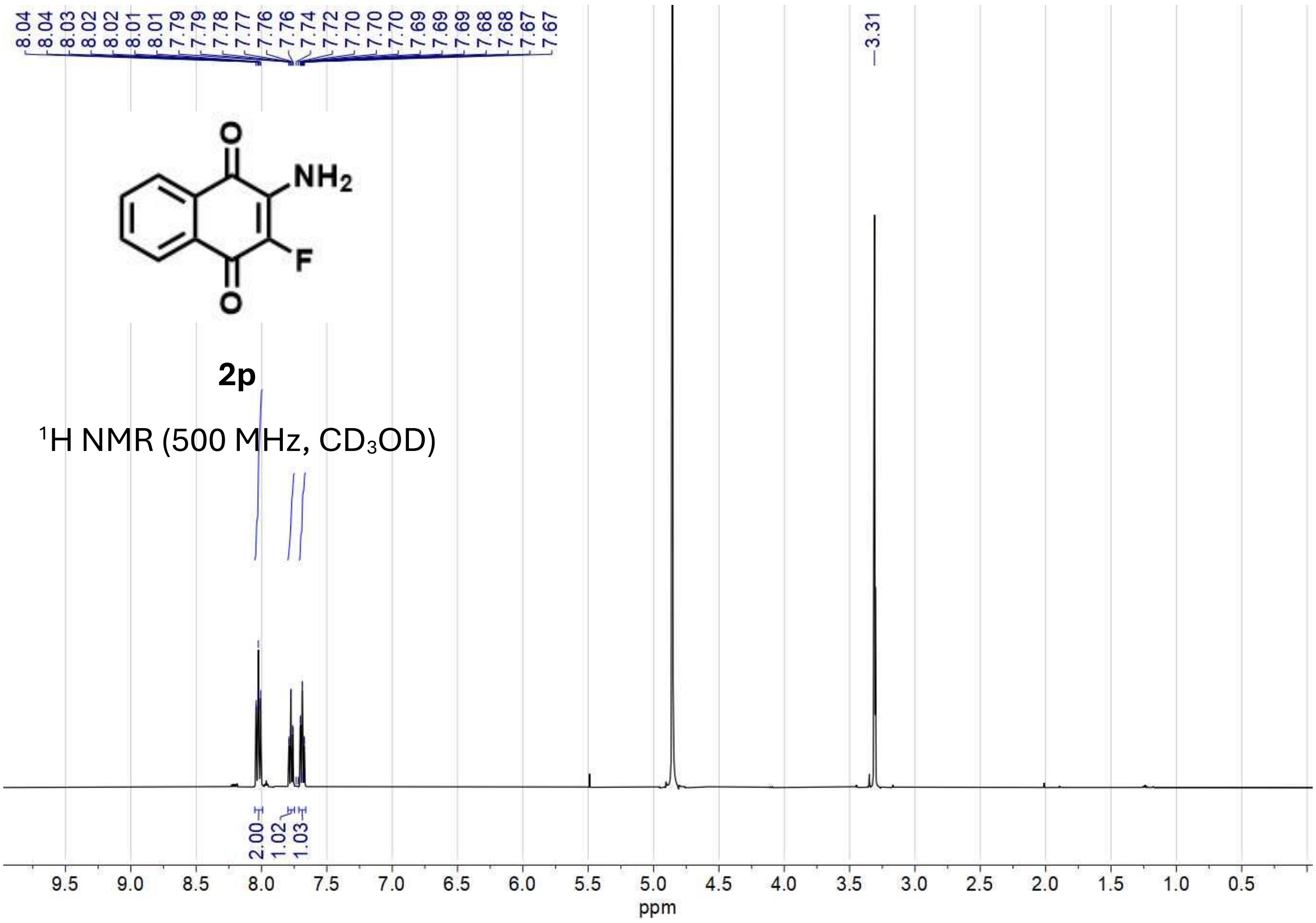

2p

^{1}H NMR (500 MHz, CD_3OD)

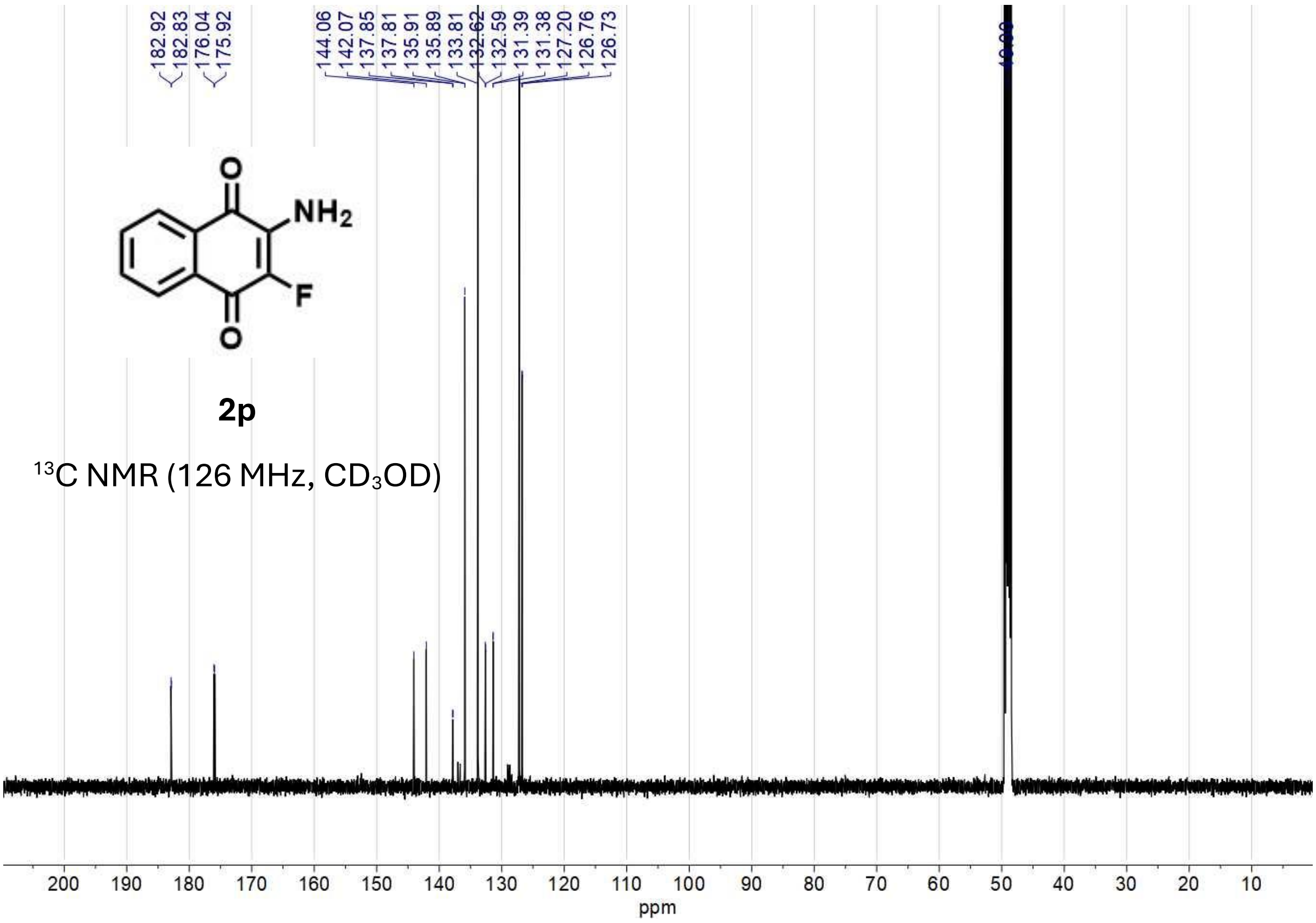

2p

^{13}C NMR (126 MHz, CD_3OD)

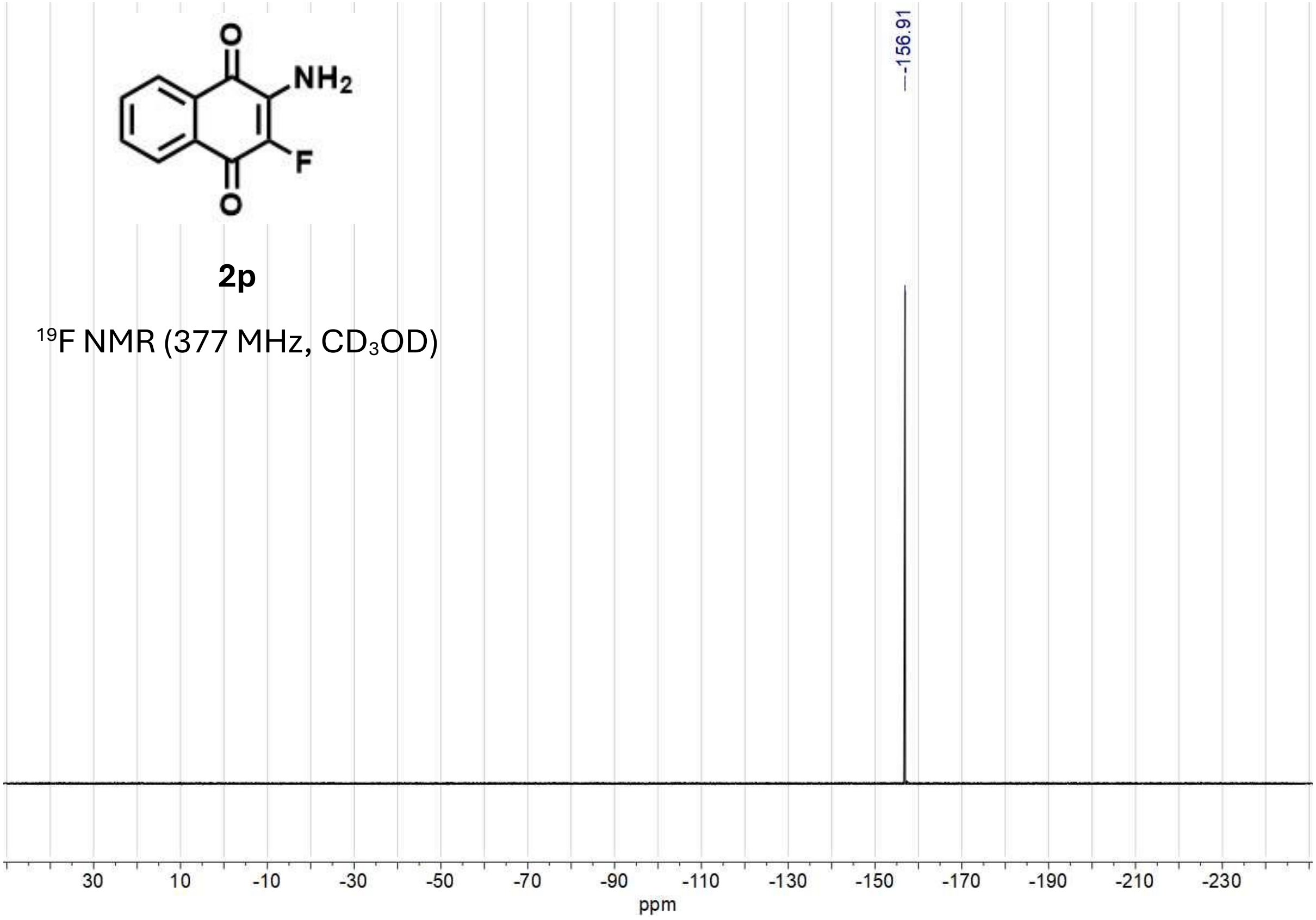
NH2
F
2p
19F NMR (377 MHz, CD3OD)
-156.91
30 10 -10 -30 -50 -70 -90 -110 -130 -150 -170 -190 -210 -230
ppm
